# Bifunctional CYP81AA proteins catalyse identical hydroxylations but alternative regioselective phenol couplings in plant xanthone biosynthesis

**DOI:** 10.1038/ncomms11472

**Published:** 2016-05-05

**Authors:** Islam El-Awaad, Marco Bocola, Till Beuerle, Benye Liu, Ludger Beerhues

**Affiliations:** 1 Institute of Pharmaceutical Biology, Technische Universität Braunschweig, Mendelssohnstraße 1, Braunschweig 38106, Germany; 2 Center of Pharmaceutical Engineering (PVZ), Technische Universität Braunschweig, Franz-Liszt-Straße 35A, Braunschweig 38106, Germany; 3 Institute of Biotechnology, RWTH Aachen University, Worringerweg 1, Aachen 52074, Germany

## Abstract

Xanthones are natural products present in plants and microorganisms. In plants, their biosynthesis starts with regioselective cyclization of 2,3′,4,6-tetrahydroxybenzophenone to either 1,3,5- or 1,3,7-trihydroxyxanthones, catalysed by cytochrome P450 (CYP) enzymes. Here we isolate and express CYP81AA-coding sequences from *Hypericum calycinum* and *H. perforatum* in yeast. Microsomes catalyse two consecutive reactions, that is, 3′-hydroxylation of 2,4,6-trihydroxybenzophenone and C–O phenol coupling of the resulting 2,3′,4,6-tetrahydroxybenzophenone. Relative to the inserted 3′-hydroxyl, the orthologues Hc/HpCYP81AA1 cyclize via the *para* position to form 1,3,7-trihydroxyxanthone, whereas the paralogue HpCYP81AA2 directs cyclization to the *ortho* position, yielding the isomeric 1,3,5-trihydroxyxanthone. Homology modelling and reciprocal mutagenesis reveal the impact of S375, L378 and A483 on controlling the regioselectivity of HpCYP81AA2, which is converted into HpCYP81AA1 by sextuple mutation. However, the reciprocal mutations in HpCYP81AA1 barely affect its regiospecificity. Product docking rationalizes the alternative C–O phenol coupling reactions. Our results help understand the machinery of bifunctional CYPs.

## Abstract (web summary)

Xanthones are pharmacologically and biosynthetically intriguing compounds. Here, the authors identify two cytochrome P450 enzymes, which hydroxylate and cyclize the benzophenone precursor to either 1,3,7- or 1,3,5-trihydroxyxanthones, and pinpoint residues that determine the alternative regioselectivities.

## Graphical abstract

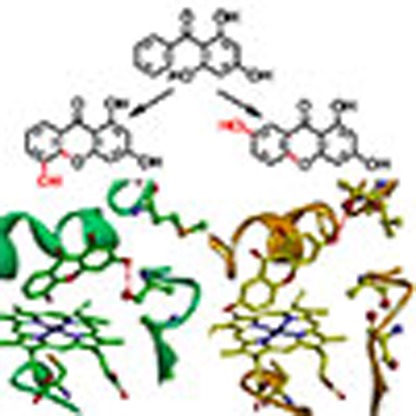

Cytochrome P450 (CYP) enzymes constitute a large superfamily of haem-thiolate proteins present in almost all living organisms^1^. They catalyse regio- and stereoselective oxidative attack of non-activated carbons at physiological conditions. In plants, CYPs are encoded by ∼1% of the protein-coding genes, the catalytic functions of the majority of the gene products remaining unknown^1^. Assigning functions to new CYPs is a challenge due to the large number of proteins, the lack of reliable sequence-function correlations, and the structural similarities between family members^1^. Commonly, CYPs in plants catalyse hydroxylation (mono-oxygenation) reactions in both the general and the specialized metabolism. However, few enzymes exhibit unusual activities^2^,^3^, including methylenedioxy bridge formation, successive oxidation of a single carbon, rearrangement of carbon skeletons, S-heterocyclization, sterol desaturation and C–C bond cleavage, as well as phenol coupling^4^,^5^,^6^,^7^. Intramolecular C–C and intermolecular C–O phenol coupling reactions were studied at the gene level in benzylisoquinoline alkaloid metabolism^7^,^8^,^8^; however, intramolecular C–O phenol coupling was only established biochemically in plant xanthone biosynthesis^10^.

Xanthones (Fig. 1a) embody a group of natural products found in fungi, lichens and higher plants^11^. Their distribution and substitution patterns are chemotaxonomically significant^12^. To date, more than 1,500 xanthone-based natural products are available in public databases. The major groups involve simple oxygenated, glycosylated and prenylated compounds beside bisxanthones, xanthonolignoids and miscellaneous xanthones^11^,^13^. Plant xanthones are defense compounds against herbivores and microorganisms^14^. In humans, xanthones exhibit an array of pharmacological activities, including anti-Alzheimer properties^11^,^15^.

Two distinct xanthone biosynthetic pathways are known. In fungi and lichens, the xanthone nucleus consists of eight acetyl units yielding an intermediate anthraquinone, which is cleaved and recyclized to give a xanthone^16^. Plants use a mixed biosynthetic pathway, which involves the shikimate and the malonate routes (Fig. 1b). Benzophenone synthase (BPS) catalyses condensation of benzoyl-CoA and three molecules of malonyl-CoA to form 2,4,6-trihydroxybenzophenone (2,4,6-triHB)^17^. Either the subsequent conversion by benzophenone 3′-hydroxylase (B3′H) or the alternative use of 3-hydroxybenzoyl-CoA by BPS yields 2,3′,4,6-tetrahydroxybenzophenone (2,3′,4,6-tetraHB)^17^,^18^. As a central branching event, this intermediate undergoes regioselective oxidative C–O phenol couplings either *ortho* or *para* to the 3′-hydroxy group, yielding 1,3,5- and 1,3,7-trihydroxyxanthones (triHXs), respectively^10^. These intramolecular cyclizations are catalysed by distinct CYPs, referred to as 1,3,5- and 1,3,7-trihydroxyxanthone synthases (1,3,5-TXS and 1,3,7-TXS). The isomeric CYP products serve as precursors for all plant xanthones^10^ and undergo further substitutions. Stepwise prenylations using C_5_ and C_10_ units and concomitant cyclizations yield complex derivatives with bridged polycyclic skeletons and high pharmaceutical potential (Fig. 1a) (refs 11, 19).

Approximately 250 CYP sequences are present in the public transcriptomes of the medicinal plant *Hypericum perforatum* (St John's wort). In combination with a subtracted cDNA library of elicitor-treated *H. calycinum* cell cultures accumulating a derivative of 1,3,7-triHX (ref. 20), cDNAs encoding 1,3,5-TXS and 1,3,7-TXS are isolated. When co-expressed in yeast (*Saccharomyces cerevisiae*) with *H. calycinum* NADPH-CYP reductase (CPR), both enzymes catalyse two consecutive reactions yielding the xanthone scaffold. However, identical hydroxylations are followed by alternative regioselective C–O phenol couplings in the paralogous CYPs. Homology modelling and reciprocal site-directed mutagenesis result in functional conversion of 1,3,5-TXS into 1,3,7-TXS and the alternative cyclizations are rationalized by product docking.

## Results

### Cloning of *CYP81AA1* from *H. calycinum* cell cultures

Yeast-extract-treated *H. calycinum* cell cultures accumulate the 1,3,7-triHX derivative hyperxanthone E (Supplementary Fig. 1), which is undetectable in non-treated cell cultures^20^. Under these conditions, a suppression subtractive hybridization (SSH) cDNA library was constructed, which was enriched in elicitor-inducible transcripts. Subsequent bioinformatic analysis of assembled contigs encoding fragments of candidate CYPs resulted in identification of CYP71, CYP72, CYP81 and CYP706 sequences (Supplementary Table 1). Notably, CYP81 was represented by multiple contigs, two of which, 21 and 59, were encoded by 22 and 33 copies, respectively, indicating high elicitor responsiveness. CYP81 belongs to the CYP71 clan comprising shikimate-modifying enzymes^21^. In contrast, the CYP72 clan represented by CYP72A with a high copy number (Supplementary Table 1) is related to isoprenoid metabolism. When searched against *H. perforatum* transcriptomes, which are publicly available in the ′Medicinal Plant Genomics Resource′ (MPGR) databank (http://medicinalplantgenomics.msu.edu), the contigs 21 and 59 shared 95 and 97.3% identity, respectively, with the predicted amino acid sequence of locus 416 and therefore appeared to be related to the same gene. To test if the non-overlapping contigs 21 and 59 belong to the same sequence, a forward primer from the upstream contig 59 and a reverse primer from the downstream contig 21 were designed. Using 5′ RACE-ready cDNA as a template, a core fragment of 1,075 bp was amplified. The lacking 204 and 248 bp portions towards the start and stop codons, respectively, were amplified using 5′ and 3′ RACE techniques, resulting in a 1,830 bp full-length cDNA, which was re-amplified by proof-reading polymerase. The 1,527 bp open reading frame (ORF) encoded a 508 amino acid protein which was named CYP81AA1 by the CYP nomenclature committee^22^. The enzyme constituted a new CYP81 subfamily.

### Cloning of *NADPH-cytochrome P450 reductase*

The activity of CYPs commonly depends on cooperation with a NADPH-CYP reductase (CPR) as electron-transfer partner^23^. The SSH cDNA library contained a CPR cDNA core fragment, which lacked 1,707 and 244 bp toward the start and stop codons, respectively. The missing 3′ stretch was amplified by 3′ RACE using RNA from elicitor-treated *H. calycinum* cell cultures. For 5′-extension, a degenerate upstream primer led to elongation by 1,362 bp and 5′ RACE then yielded the remaining 345 bp up to the start codon. The 2,599 bp full-length cDNA, which was re-amplified by proof-reading polymerase, contained a 2,139 bp ORF encoding a 712 amino acid protein. This enzyme shared 75, 79.1 and 79.2% identity with the CPR2 enzymes from *Arabidopsis thaliana*^24^, *Gossypium hirsutum*^25^ and *Populus trichocarpa*^26^, respectively, and was therefore designated as HcCPR2. In contrast to animals which have a single CPR^27^, plants contain multiple CPR isoforms which group in two classes distinguishable by their divergent N-termini^26^,^28^. In *Arabidopsis*, cotton and centaury, expression of class II *CPR* is induced by wounding, light and elicitation, whereas class I *CPR* is constitutively regulated^24^,^25^. Consistently, class II CPR transcripts were cloned from the SSH cDNA library of *H. calycinum* and isolated from cell cultures after elicitor treatment. The full-length HcCPR2 cDNA was cloned in MCS2 of the pESC-URA vector and expressed in yeast. Microsomes were isolated and the ability of HcCPR2 to transfer electrons from NADPH to cytochrome c was verified^29^. The *K*_m_ (Michaelis constant) values for NADPH and cytochrome c were 43.9±6.5 and 35.1±5.7 μM, respectively.

### *H. calycinum CYP81AA1* encodes 1,3,7-TXS

**Figure 2:**
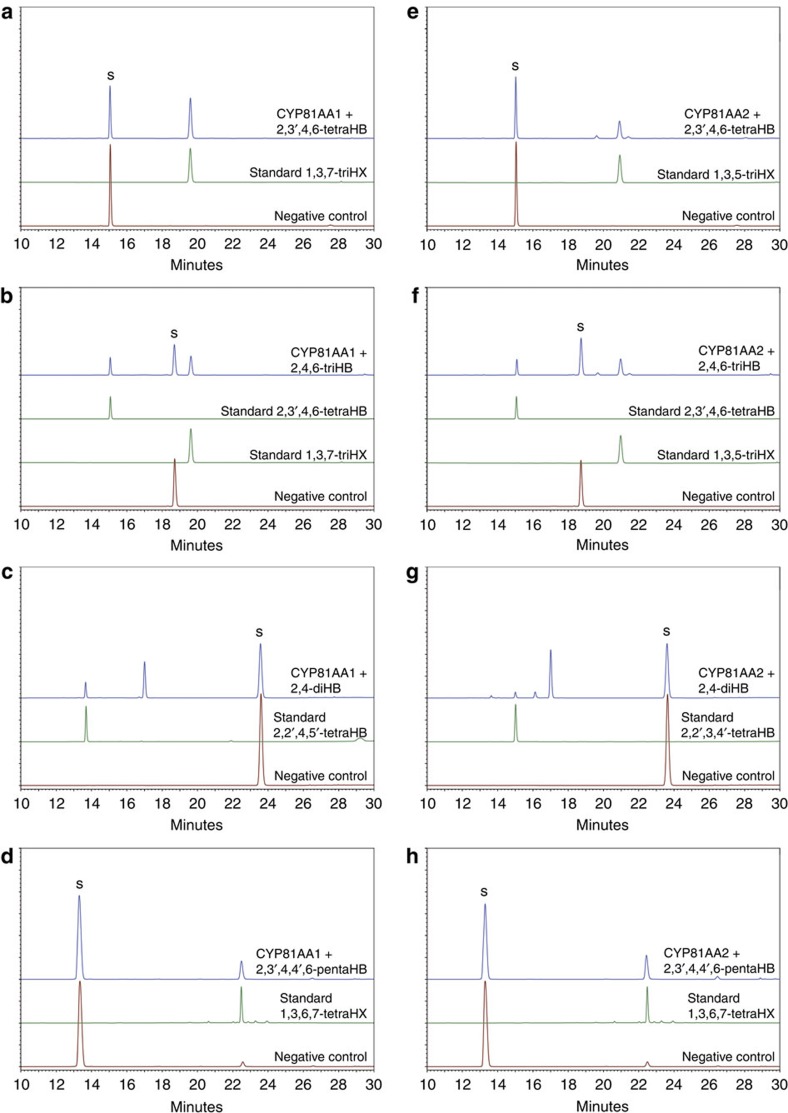
Enzymatic activities of CYP81AA1 and CYP81AA2. Yeast microsomes containing CYP81AA1 (**a–d**) and CYP81AA2 (**e–h**) were incubated with the substrates indicated. The enzyme assays were analysed by HPLC-DAD. Control assays lacked NADPH. diHB, dihydroxybenzophenone; pentaHB, pentahydroxybenzophenone; S, substrate; triHB, trihydroxybenzophenone; tetraHB, tetrahydroxybenzophenone; triHX, trihydroxyxanthone; tetraHX, tetrahydroxyxanthone. Detection wavelength: 260 nm.

**Figure 3:**
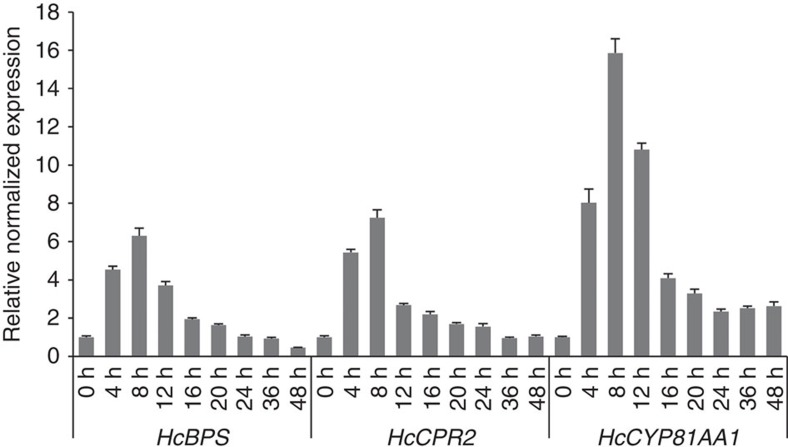
Gene expression analysis. Changes in the transcript levels of *HcBPS*, *HcCPR2* and *HcCYP81AA1* in elicitor-treated *Hypericum calycinum* cell cultures were analysed relative to those in untreated control cultures. Relative normalized expression values were determined by RT-qPCR. Data represent means±s.e.m (*n*=3). RT-qPCR, reverse transcription quantitative real-time PCR.

The HcCYP81AA1 and HcCPR2 coding sequences were ligated into the multicloning sites 1 and 2, respectively, of the pESC-URA vector. After co-expression in yeast, microsomes were isolated and incubated with eleven potential benzophenone and xanthone substrates in the presence of NADPH (Supplementary Table 2, Supplementary Fig. 2 and Supplementary Methods). Products formed were analysed by high-performance liquid chromatography (HPLC). Incubation with 2,3′,4,6-tetraHB resulted in formation of a single product, whose retention time (*R*_t_ 19.6 min) matched that of authentic 1,3,7-triHX (Fig. 2a). In addition, the UV and mass spectra agreed with those of the reference compound (Supplementary Figs 3 and 4). When incubated with 2,4,6-triHB, the microsomal fraction formed two products, whose *R*_t_ values (15.1 and 19.6 min) matched those of authentic 2,3′,4,6-tetraHB and 1,3,7-triHX, respectively (Fig. 2b). The identities were verified by mass spectrometry (MS/MS) and UV spectroscopy (Supplementary Figs 3 and 4). Incubation with 2,4-dihydroxybenzophenone (2,4-diHB) also yielded two products (Fig. 2c). The product with *R*_t_ 13.7 min was identified as 2,2′,4,5′-tetrahydroxybenzophenone (2,2′,4,5′-tetraHB) in comparison with authentic compound (Supplementary Figs 3 and 4). The product with *R*_t_ 17.1 min was tentatively identified as 2,3′,4-trihydroxybenzophenone (2,3′,4-triHB) using MS/MS (Supplementary Fig. 4). Incubation with 2,3′,4,4′,6-pentahydroxybenzophenone (2,3′,4,4′,6-pentaHB, maclurin) yielded a single product, which shared the *R*_t_ value (22.3 min) and the UV and mass spectra with authentic 1,3,6,7-tetrahydroxyxanthone (1,3,6,7-tetraHX, norathyriol) (Fig. 2d and Supplementary Figs 3 and 4). Traces of this compound were also detected in control incubations, confirming its previously reported presence as a contaminant in 2,3′,4,4′,6-pentaHB samples^30^. Control assays without NADPH failed to form enzymatic products from all substrates used. Likewise, microsomes from yeast harbouring the empty plasmid did not produce any of the products. No enzymatic activity was observed with benzophenones and xanthones illustrated in Supplementary Fig. 2. Thus, HcCYP81AA1 was identified as a bifunctional enzyme, which exhibits both B3′H and 1,3,7-TXS activities. To correlate the expression profiles of *HcCYP81AA1*, *HcCPR2* and *HcBPS* with the previously published accumulation pattern of hyperxanthone E in elicitor-treated *H. calycinum* cell cultures^20^, changes in the transcript levels were analysed over 48 h by reverse transcription quantitative real-time PCR (RT-qPCR) (Fig. 3). *Actin* and *Histone H2A* served as reference genes. The transcript levels of all genes started to increase 4 h post elicitation, peaked at 8 h and decreased thereafter. Their increases thus preceded the accumulation of hyperxanthone E, which started 12 h post elicitation^20^.

### *H. perforatum* contains *HcCYP81AA1* homologues

*H. perforatum* synthesizes both 1,3,7- and 1,3,5-triHX derivatives^31^, indicating occurrence of *1,3,7-TXS* and *1,3,5-TXS* genes. The HcCYP81AA1 sequence was used to search for homologues in the publicly available transcriptomes of *H. perforatum* present in the MPGR databank. Four closely related CYP loci (416, 928, 8128 and 51544) were identified and shared 95.3, 69.7, 66.5 and 66.9% amino acid sequence identities with HcCYP81AA1, respectively. The locus 51544 lacked about 430 bp towards the stop codon and all trials to complete this sequence failed, suggesting a pseudogene. Furthermore, expression of this gene was low in all organs studied, as indicated by the ‘fragments per kilobase per million mapped fragments' (FPKM) values in the MPGR database. In contrast, the other three loci existed as full-length sequences in the databank. The FPKM values of the loci 416 and 928 indicated preferential expression in roots and leaves, which is in accordance with the tissue distribution pattern of BPS transcripts (locus 13339) (Supplementary Fig. 5). In contrast, transcripts encoded by locus 8128 were more abundant in flower organs.

### HpCYP81AA1 is 1,3,7-TXS and HpCYP81AA2 is 1,3,5-TXS

Primers with appropriate restriction sites were used to clone the coding sequences of the loci 416, 928 and 8128 into the multicloning site 1 of the pESC-URA:HcCPR2 plasmid to be transferred to yeast for co-expression. Microsomes were incubated with eleven potential substrates in the presence of NADPH. The activity profile of the enzyme encoded by locus 416 resembled that of HcCYP81AA1 (Supplementary Table 2). The deduced amino acid sequence was referred to as HpCYP81AA1, an orthologue of *H. calycinum* 1,3,7-TXS. The two proteins differed in only 24 amino acids.

The enzyme encoded by the ORF of locus 928 was named CYP81AA2, a paralogue of CYP81AA1. The pESC-URA:HpCYP81AA2/HcCPR2 plasmid was constructed for functional co-expression in yeast (Supplementary Table 2 and Supplementary Fig. 2). Incubation of microsomes with 2,3′,4,6-tetraHB led to formation of one major product (79.5±1.1% of total product), which co-eluted (*R*_t_ 20.9 min) with authentic 1,3,5-triHX (Fig. 2e). The UV and mass spectra of this enzymatic product matched those of the reference compound (Supplementary Figs 6 and 7). Two minor products were also detected, one of which (*R*_t_ 19.6 min) was identified as 1,3,7-triHX (11.0±0.2%), while the second (*R*_t_ 21.3 min; 9.5±0.9%, based on the 1,3,7-triHX standard curve) could not be identified. Incubation with 2,4,6-triHB resulted in formation of the same products in addition to 2,3′,4,6-tetraHB (Fig. 2f and Supplementary Figs 6 and 7). In incubations with 2,4-diHB (Fig. 2g), the major product (R_t_ 17.1 min) was 2,3′,4-triHB, as suggested by the mass spectrum (Supplementary Fig. 7). Of two additional minor products, the first one (*R*_t_ 15.1 min) was identified as 2,2′,3,4′-tetrahydroxybenzophenone (2,2′,3,4′-tetraHB) (Supplementary Figs 6 and 7). The other (*R*_t_ 16.2 min) could not be identified; however, xanthone cyclization products of 2,2′,3,4′- and 2,2′,4,5′-tetraHBs are ruled out. Unexpectedly, incubation with 2,3′,4,4′,6-pentaHB resulted in formation of 1,3,6,7-tetraHX rather than 1,3,5,6-tetraHX (Fig. 2h and Supplementary Figs 6 and 7). No enzymatic products were detected in assays that either lacked NADPH or contained microsomes from yeast harbouring the empty plasmid. The compounds depicted in Supplementary Fig. 2 failed to act as substrates. In consequence, HpCYP81AA2 was identified as a bifunctional enzyme, which possesses both B3′H and 1,3,5-TXS activities.

The protein encoded by locus 8128 was named HpCYP81AA3. The only reaction catalysed was formation of 1,3,6,7-tetraHX from 2,3′,4,4′,6-pentaHB (Supplementary Table 3).

### Molecular modelling suggests residues for mutagenesis

**Figure 4:**
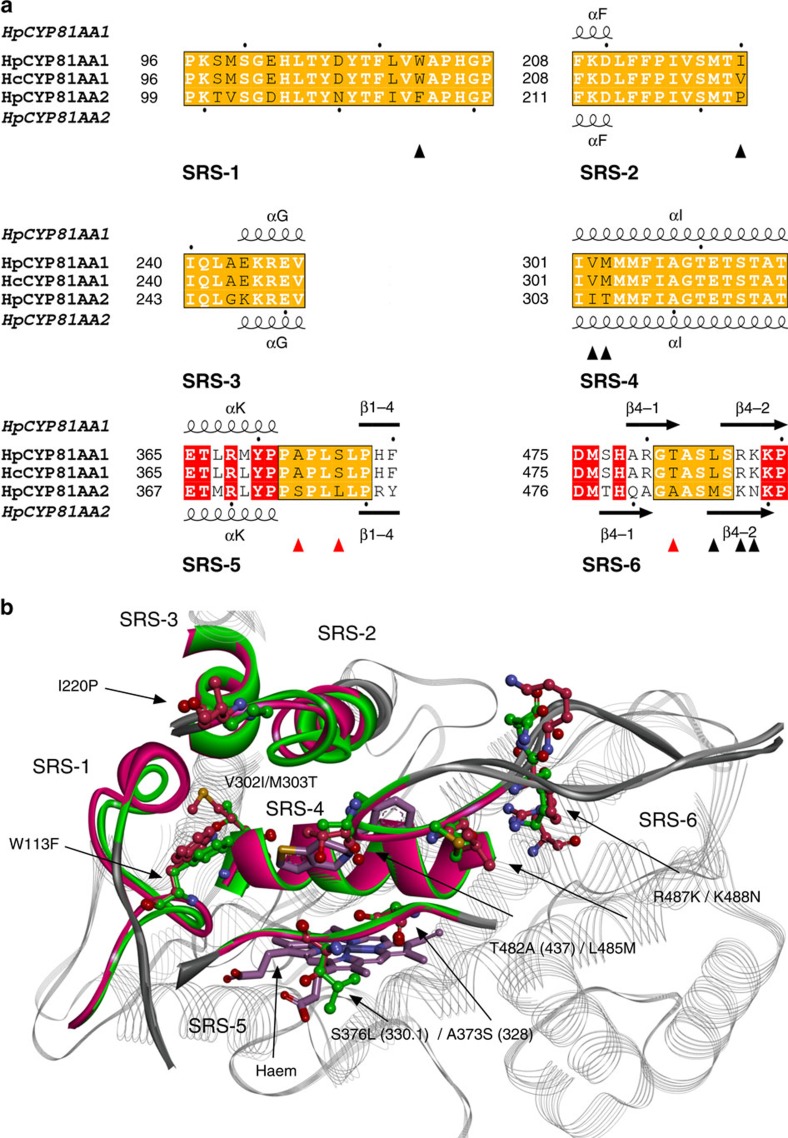
Residues selected as targets for site-directed mutagenesis. (**a**) Alignment of the SRS sequences of HpCYP81AA1, HcCYP81AA1 and HpCYP81AA2. Amino acids expected to fall within SRSs 1–6 are highlighted by orange boxes. Dissimilar residues are written in black letters and conserved residues outside the SRSs are represented in white with a red background. The conserved secondary structures of HpCYP81AA1 and HpCYP81AA2 are indicated at the top and the bottom, respectively. The black triangles point to positions selected for mutation and the red triangles refer to the major regioselectivity-determining residues in HpCYP81AA2. (**b**) Structural overlay of the homology models of HpCYP81AA1 (magenta, dark grey) and HpCYP81AA2 (green, light grey). Coloured parts indicate the location of the six SRSs around the haem-containing active centre with bound inhibitor ticlopidine (shown in stick representation) from the template X-ray structure 3kw4 (mammalian CYP2B4). Divergent residues are shown in ball and stick, labelling being based on CYP81AA1 numbering. Standard positions for major regioselectivity-determining residues are put in brackets.

To gain insight into the regioselectivity-determining elements of CYP81AA1 and CYP81AA2, the amino acid sequences were aligned, secondary structure elements were predicted^32^, residues were numbered according to the standardized numbering scheme for class II CYPs^33^, and the putative six substrate recognition sites (SRSs) were identified^34^,^35^. The predicted secondary structure elements of the two enzymes matched, except for β3-3, β4-1 and β4-2, located in the C-terminal region (Fig. 4a and Supplementary Fig. 8). Therefore, the 55 C-terminal amino acids were reciprocally exchanged between the two enzymes to identify selectivity-determining residues. However, replacement of this portion of CYP81AA2 with that of CYP81AA1 resulted in a complete loss of enzyme activity and the chimeric protein was not subjected to further mutations.

A second round of mutations involved reciprocal site-directed mutagenesis of individual amino acids located in the SRSs. The two CYP81AA1 orthologues differed in only one residue (V/I220, standard position 189) within the six predicted SRSs. In contrast, HpCYP81AA1 and HpCYP81AA2 had a total of 145 divergent residues including 15 within the SRSs plus 7 gaps, which were expected to be responsible for the alternative regioselectivities (Fig. 4a and Supplementary Fig 8). Therefore, changing one or more of these residues towards their counterparts in the other enzyme was likely to affect the nature of the cyclization product. To reduce the number of possible single and multiple reciprocal mutations, homology models of both enzymes were built using two independent methods, YASARA and HHpred^36^. The final HHpred models were based on the structure of the closed conformation of mammalian CYP2B4 in complex with the inhibitor ticlopidine (3kw4) (ref. 37). Upon examination of the superimposed models, 17 residues were located within a 4 Å radius of the bound inhibitor in either protein model. Five of these residues belonging to the SRSs 5 and 6 differed between the two enzymes (Supplementary Table 4 and Fig. 4b). In addition, S375 in CYP81AA1, which corresponds to L378 in CYP81AA2 (standard position 330.1), was selected as a mutagenesis target. This position undergoes a change from hydrophilic to hydrophobic and was previously predicted to affect the selectivity of CYPs^35^.

### A sextuple mutation converts 1,3,5-TXS into 1,3,7-TXS

**Figure 5:**
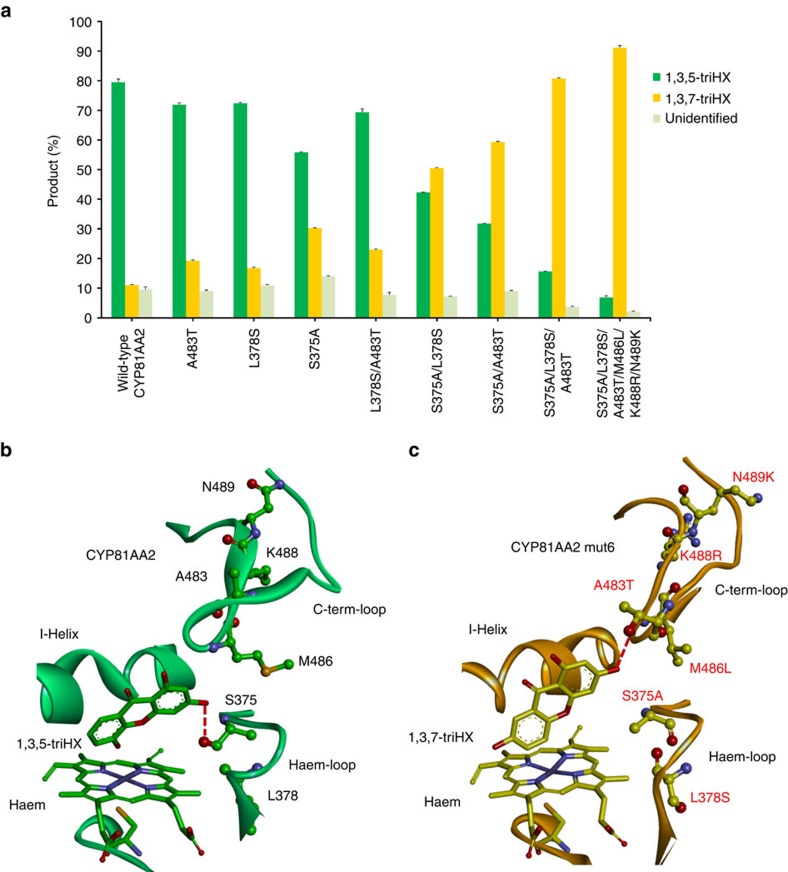
Inversion of the regioselectivity of CYP81AA2. (**a**) Product profiles of wild-type HpCYP81AA2 and mutant enzymes. Data represent means±s.d. (*n*=4). (**b**) Product docking showing the active site of wild-type CYP81AA2 (lime) with bound 1,3,5-triHX (green sticks). The 3-hydroxy group forms a hydrogen bond with S375, orienting it towards the haem loop. (**c**) The active site of the sextuple mutant (mut6) of CYP81AA2 (orange) with bound 1,3,7-triHX (yellow sticks). The 3-hydroxy group forms a hydrogen bond with A483T, orienting it toward the C-term-loop.

Wild-type CYP81AA2 released 79.5% of the total product as 1,3,5-triHX and 11.0% as 1,3,7-triHX. Reciprocal mutation of six individual residues in SRSs 5 and 6 towards their counterparts in CYP81AA1 led to a gradual change in the 1,3,5-triHX to 1,3,7-triHX ratio (Fig. 5a). In the homology model built, the standard positions 437 and 328 were the closest divergent contact residues of the bound inhibitor. Reciprocal single substitutions at the positions 437 in SRS-6 (A483T) and 328 in SRS-5 (S375A) increased the 1,3,7-triHX portion from 11.0 to 19.2 and 30.2%, respectively. Single substitution at the selected standard position 330.1 in SRS-5 (L376S) yielded only 16.7% 1,3,7-triHX. Of the double mutants generated, L378S/A483T formed 22.9% 1,3,7-triHX; however, S375A/L378S and S375A/A483T, which contained the standard position 328, resulted in 50.5 and 59.3% 1,3,7-triHX, respectively. This outcome was increased to 80.7% by combining all three positions in the S375A/L378S/A483T mutant. Finally, the sextuple mutant S375A/L378S/A483T/M486L/K488R/N489K (mut6), which involved all mismatching contact residues, released 91.1% of the total product as 1,3,7-triHX (Fig. 5a). None of the mutants generated could mimic the absolute regiospecificity of CYP81AA1 towards formation of 1,3,7-triHX. No appreciable changes in the level of the unidentified product were observed.

### Changing the 1,3,7-TXS regiospecificity remains a challenge

Wild-type CYP81AA1 showed absolute specificity towards the production of 1,3,7-triHX. Substitution of the 55 C-terminal residues by those of CYP81AA2 failed to cause any change in the regiospecificity. Similarly, reciprocal mutations in SRSs 5 and 6 had only minor effects on the regioselectivity (Supplementary Table 5). For example, the sextuple mutation A373S/S376L/T482A/L485M/R487K/K488N caused only 6.5% of the total product to be released as 1,3,5-triHX. This result resembled the effect of the single mutant A373S, which formed 5.1% 1,3,5-triHX. Replacing alanine in the T482A position of the triple mutant (A373S/S376L/T482A) with larger hydrophobic residues (T482V and T482F) failed to further shift the regioselectivity. Finally, a third set of mutations were created by selecting divergent residues within an increased radius (<15 Å of the inhibitor ticlopidine), such as W113F in SRS-1, I220P in SRS-2, and V302I/M303T in SRS-4. However, none of the additional mutations in SRSs 1 and 2 led to further changes in the regioselectivity. Mutations performed on SRS-4 even resulted in the loss of enzyme activity.

### Docking rationalizes the alternative regioselectivities

Docking of 1,3,5- and 1,3,7-triHX in a catalytic binding orientation, as detailed in Methods, into the homology models of wild-type CYP81AA2 and its sextuple mutant S375A/L378S/A483T/M486L/K488R/N489K (mut6) revealed different modes of product binding, indicating hindrance of internal rotation in the 2,3′,4,6-tetraHB substrate (Fig. 5b). In the wild-type enzyme (1,3,5-TXS), the 3-hydroxy group of the product can form a hydrogen bond with S375, orienting it toward the haem loop. The 5-hydroxy group points toward the haem group. At the substrate level, rotation of the 3′-hydroxyphenyl ring of 2,3′,4,6-tetraHB is hindered by the side chains of the I-helix residues. In the sextuple mutant, the 3-hydroxy group of the product can form a new hydrogen bond with A483T, orienting it towards the C-terminal loop. This hydrogen bond switching can explain the observed cooperative effect of the double mutant S375A/A483T. The mutation introduced in the former H-bonded residue S375A together with the nearby mutation L378S may alter the conformation of the haem loop to cooperatively support the reorientation of the substrate. The 7-hydroxy group of the product is located between the I-helix and the haem group, which again indicates hindrance of free rotation of the 3′-hydroxyphenyl ring of 2,3′,4,6-tetraHB.

## Discussion

The interface between the benzophenone and xanthone biosynthetic pathways is a ring closure reaction, which converts the diphenyl ketone scaffold to a tricyclic ring system. This cyclization step is preceded by 3′-hydroxylation, which favours subsequent substitution at the *ortho* and *para* positions of the benzophenone. Demonstrated herein is that both consecutive reactions are catalysed by bifunctional CYP, two variants of which accomplish the ring closure reaction in a regioselective manner. Hydroxylation by the oxygen rebound mechanism is the classical CYP-catalysed reaction, inserting one atom of molecular oxygen into the substrate and reducing the second to water^38^. The major oxidant intermediate in CYP reactions is the so-called compound I, a highly reactive iron-oxo radical species^38^,^39^. However, benzophenone cyclization was proposed to be an oxidative phenol coupling, reducing both atoms of molecular oxygen to water^2^,^3^. The TXSs catalyse both the hydroxylation and the coupling reactions and thus function as mono-oxygenase and oxidase (Supplementary Fig. 9). The previously proposed coupling mechanism involves two one-electron oxidations^10^. Formation of an intermediate biradical, as offered for phenol couplings in morphine, corytuberine and cyclodipeptide biosyntheses^8^,^40^,^41^, is energetically unfavourable and the underlying mechanisms have recently been revised^3^,^7^,^10^,^42^.

Homology modelling and product docking revealed that CYP81AA1 and CYP81AA2 accommodate distinct conformers of 2,3′,4,6-tetraHB. The 3′-hydroxyphenyl ring lacks free rotation in the active site cavities, resulting in cyclization to 1,3,7- and 1,3,5-triHXs, respectively. At the xanthone level, no hydroxylation by the two CYPs takes place, as indicated by the non-acceptance of 1,3-dihydroxyxanthone and derivatives. Notably, 2,4-diHB as substrate was 3′-hydroxylated by both CYP81AA1 and CYP81AA2; however, the identical product 2,3′,4-triHB lacked cyclization because of the absence of the 6-hydroxy group. Instead, it underwent 2′-hydroxylation, yielding 2,2′,4,5′- and 2,2′,3,4′-tetraHBs, respectively. Subsequent loss of water, as previously proposed for *ortho*-*ortho′*-dihydroxylated benzophenones^43^, was hindered by hydrogen bonding between the 2-hydroxy and the carbonyl groups. However, 2′-hydroxylation and water elimination may be an alternative reaction mechanism for the physiological substrate 2,4,6-triHB (ref. 43). With 2,3′,4,4′,6-pentaHB ring closure took place but lacked regioselectivity and both enzymes formed 1,3,6,7-tetraHX. This conversion was the only reaction catalysed by CYP81AA3, the physiological function of which remains open. None of the CYPs studied here converted monohydroxylated benzophenones, such as 2- and 4-hydroxybenzophenones.

Identification of genes encoding TXSs with alternative regioselectivities offered the chance of exploring their selectivity determinants. Sequence comparison, homology modelling, and reciprocal site-directed mutagenesis demonstrated the impact of individual SRS residues on the selection of the alternative substrate conformers. CYP81AA2 (1,3,5-TXS) was almost completely converted into CYP81AA1 (1,3,7-TXS). Three amino acids in close proximity to the haem group (S375, L378 and A483; equivalent to the standard positions 328, 330.1 and 437, respectively^33^,^44^) were most influential in orienting the coupling reaction *ortho* to the 3′-hydroxy group. S375 and L378 are located within SRS-5, which spans the loop between the EXXR motif and the *β*1-4 strand, whereas A483 belongs to SRS-6. S375 (standard position 328) occupies position 5 behind the EXXR motif, its side chain pointing towards the haem group in the majority of structurally studied CYPs. The position is a mutation hotspot^35^,^44^ and preferentially occupied by a hydrophobic amino acid^35^. However, CYP81AA2 contains a polar serine residue, which may be required for the interaction with the polyhydroxylated substrates. In a number of CYPs, mutations at this standard position 328 resulted in dramatic changes in the regio- and stereoselectivities^35^,^44^,^45^. The point mutation F363I entirely switched the regiospecificity of spearmint limonene 6-hydroxylase to that of peppermint limonene 3-hydroxylase^45^. In CYP81AA2, however, the point mutation S375A caused only a partial change (∼20%) towards 1,3,7-triHX formation.

In addition to standard position 328, a second protruding residue in SRS-5 is expected to occupy either position 8 or 9 after the EXXR motif (corresponding to standard positions 331 and 332, respectively)^35^. Although filling position 8 behind the EXXR motif, the L378 residue was assigned the standard position 330.1, for which surprisingly no mutations were reported. Thus, the involvement of 330.1 provides new combinations of regioselectivity determinants. In the model of the sextuple mutant, 328 and 330.1 change the orientation of the haem loop, thereby affecting the size of the active site.

SRS-6 is formed by the turn in *β*-sheet 4 between *β*4-1 and *β*4-2 towards the C-terminus. A483 (standard position 437) is located at the tip of this turn and its side chain protrudes into the active site (Fig. 4b). Mutations at this position affected the regiospecificities of multiple CYPs^44^, such as CYP94A2, in which the F494L substitution shifted the regioselectivity of fatty acid hydroxylation from ω to ω-1 (ref. 46). In CYP81AA2, L378S and A483T caused less than 8% change each but induced ∼20 and 29% shifts, respectively, in combination with S375A, indicating synergistic interaction in controlling the regioselectivity. The triple mutant S375A/L378S/A483T caused 80.7% of the total product to be released as 1,3,7-triHX. Investigation of further CYP81AA proteins for sequence/structure-function relationships, with an emphasis on SRS-5 and SRS-6, will clarify whether the positions are generally key mediators of regioselectivity. Another 10.4% shift in selectivity was contributed by the triple mutation M486L/K488R/N489K in SRS-6. However, no combination of mutations was capable of achieving the absolute regiospecificity of CYP81AA1. In case of the sextuple mutant (mut6), a portion of 6.9% of the total product was still 1,3,5-triHX (besides 2% unidentified product).

Reciprocal site-directed mutagenesis was also applied to CYP81AA1 (1,3,7-TXS); however, the maximum shift in the regioselectivity achieved was 6.8%, indicating that global changes in the backbone rather than subtle substitutions in the SRSs are needed to convert 1,3,7-TXS to 1,3,5-TXS. Similarly, the I364F modification in the peppermint limonene 3-hydroxylase, which is the reciprocal mutation to the abovementioned substitution, did not achieve the regiospecificity of the spearmint limonene 6-hydroxylase; quite the contrary, it afforded an inactive, although properly folded, enzyme^45^. Crystallization of the membrane anchor-freed TXSs is necessary to gain deeper insight into the structural conditions and the reaction mechanisms underlying the bifunctionality. Notably, none of the enzyme mutants generated exhibited uncoupling of the consecutive hydroxylation and coupling reactions.

Besides intramolecular C–O phenol coupling studied here, two other types of phenol coupling were detected previously in plant benzylisoquinoline alkaloid biosynthesis (Supplementary Fig. 10). CYP80G2 and CYP719B1 catalyse stereoselective intramolecular C–C phenol coupling, which converts (*S*)- and (*R*)-reticuline to (*S*)-corytuberine and salutaridine, respectively^7^,^8^. CYP80A1 accomplishes intermolecular C–O phenol coupling between (*R*)- and (*S*)-*N*-methylcoclaurine to form the bisbenzylisoquinoline alkaloid berbamunine^9^. None of these enzymes exhibited multifunctionality. In contrast to these CYP80 and CYP719 families, which are represented by a limited number of members in a restricted quantity of plant taxa^47^, CYP81 is a large enzyme family consisting of multiple members in all plant genomes sequenced to date^48^. However, only a few members of the CYP81 family were functionally characterized, linking them to specialized metabolism in response to biotic and abiotic stress^49^. CYP81E members catalyse regiospecific hydroxylations on ring B of the isoflavone skeleton in either the 2′- or 3′-positions, yielding products for pathogen defense and insect-induced responses, respectively^50^. CYP81Q members catalyse stepwise formation of two methylenedioxy bridges in the biosynthesis of the lignan (+)-sesamin, which widely occurs in vascular plants^5^. In microorganisms, four CYPs (OxyA-C and E) catalyse intramolecular C–O and C–C phenol couplings in the biosynthesis of glycopeptide antibiotics, such as vancomycin and teicoplanin. However, these soluble bacterial CYPs do not act on the free substrates but the intermediate heptapeptides are covalently bound to a peptidyl carrier protein domain of the non-ribosomal peptide synthase, the X-domain of which recruits the CYPs^51^.

CYPs are probably nature's most versatile enzymes in terms of substrate range and reaction type^38^. This vast repertoire is here extended by characterization of CYP81AA1 and CYP81AA2. Their investigation provides deeper insight into CYP-catalysed oxidative phenol coupling reactions, which cannot be easily rationalized in context of the traditional catalytic cycle of CYPs^42^,^52^. However, more work has to be done to completely understand the selectivity determinants of CYP81AA1 and CYP81AA2, as pointed out by the challenge to convert 1,3,7- into 1,3,5-TXS. As ring closure catalysts, the enzymes may be attractive for engineering approaches, such as selective production of either 1,3,5- or 1,3,7-triHXs.

## Methods

### RNA extraction

*H. calycinum* cell cultures growing in the dark at 25 °C in liquid LS medium with shaking at 120 r.p.m. were treated with 3 g l^−1^ yeast extract and cells were collected 7 h post elicitation. Total RNA was extracted using the RNeasy Plant Mini Kit (Qiagen). Middle-aged leaves of *H. perforatum* growing in the medicinal plants garden of the Institute of Pharmaceutical Biology, Technische Universität Braunschweig, were collected during the flowering period and total RNA was extracted using the GeneJET Plant RNA Purification Mini Kit (Thermo Scientific), following the manufacturer's protocol for polyphenol-rich samples.

### Bioinformatic analysis of *H. calycinum* cDNA library

An SSH library was constructed using control vs yeast-extract-treated *H. calycinum* cell cultures^20^. The library comprised 2,005 clones, which were assembled into 277 contigs using the CAP3 program^53^. The contigs were functionally annotated using Blast2GO (ref. 54). Contigs that were annotated to encode CYPs were individually blasted against the non-redundant protein database of NCBI (NR). For the contigs having E-values lower than 1.0 E–6 with one or more CYP sequences in the database, the subfamily of the closest classified CYP and the number of copies per contig (retrieved from the CAP3 output) were examined (Supplementary Table 1). In addition, a fragment encoding CPR was also identified.

### Extension of the CPR core fragment in 5′ direction

Multiple sequence alignment of the nucleotide sequences of 20 plant CPRs from the NCBI database, whose accession numbers are listed in Supplementary Table 6, was created using ClustalW (Supplementary Fig. 11). An upstream forward degenerate primer for CPRs was designed based on the sequence of the conserved FMN binding domain. The CPR core fragment identified by bioinformatical analysis of the subtracted cDNA library lacked 1,707 bp towards the start codon. Standard PCR using the designed degenerate primer (CPR-dpF-1) and a reverse gene-specific primer (GSP) (CPR-R1; Supplementary Table 7) together with 5′-RACE-ready cDNA as a template led to extension of the core fragment by 1,362 bp in the 5′ direction. 5′-RACE primers were derived based on the sequence of the extended part.

### 3′ and 5′ rapid amplification of cDNA ends (RACE)

The SMART RACE cDNA Amplification Kit (Clontech) was used. First-strand cDNA was synthesized from 5 μg RNA, primed with the adaptor-linked oligo(dT) primer 3′-CDS, as described in the instructions for the synthesis of 3′-RACE-ready cDNA. In case of 5′-RACE, the reverse GSPs used to synthesize the first-strand 5′-RACE-ready cDNA of the respective genes were CYP81-5RACE1 and CPR-5RACE1 (Supplementary Table 7). Touchdown PCR was employed for the subsequent amplification step. The 25 μl reaction mixture contained 1 μl each of the cDNA samples in 1 × reaction buffer Y, 0.4 mM dNTPs, 0.4 μM primers and 1.25 U peqGOLD *Taq*-DNA-Polymerase (Peqlab). The PCR conditions were as follows. Initial denaturation at 94 °C for 2 min; 10 cycles with 94 °C for 30 s, annealing at the *T*_m_ of the respective GSPs for 45 s with Δ*T* −0.5 °C per cycle, 72 °C for 90 s; 30 cycles with 94 °C for 30 s, annealing at the *T*_m_ of the respective GSPs −5 °C for 45 s, 72 °C for 2 min; and final extension at 72 °C for 15 min. The RACE products were run on an agarose gel stained with Midori Green (Nippon Genetics) and the bands at the expected size were excised and purified using the innuPREP DOUBLEpure Kit (Analytic Jena). The purified products were cloned into the pGEM-T Easy vector (Promega) and sequenced. The primers used for the RACE reactions are listed in Supplementary Table 7.

### Construction of the expression plasmids

HcCPR2 was amplified with the primers HcCPR2-*Bam*HI-F and HcCPR2-*Hin*dIII-R (Supplementary Table 7) using Phusion Hot Start II High-Fidelity DNA Polymerase (Thermo Scientific), digested with *Bam*HI/*Hin*dIII and cloned into MCS2 of the pESC-URA yeast expression vector to generate the pESC-URA:HcCPR2 plasmid. The *CYP* genes were subsequently amplified with the respective primers listed in Supplementary Table 7, digested with the appropriate restriction enzymes and cloned into MCS1 of the pESC-URA:HcCPR2 plasmid to generate the pESC-URA:HcCYP81AA1/HcCPR2, pESC-URA:HpCYP81AA1/HcCPR2, pESC-URA:HpCYP81AA2/HcCPR2 and pESC-URA:HpCYP81AA3/HcCPR2 expression plasmids.

### Gene expression analysis by reverse transcription quantitative PCR

Total RNA was extracted from non-treated (control) and yeast-extract-treated *H. calycinum* cell cultures at 4, 8, 12, 16, 20, 24, 36 and 48 h post elicitation using the RNeasy Plant Mini Kit (Qiagen). On-column digestion was applied to each sample using the RNase-Free DNase Set (Qiagen) to get rid of any genomic DNA contamination. The RNA quality was checked on a gel. Concentrations and 260/280 ratios were determined using the SimpliNano Spectrophotometer (GE Lifesciences). For each time point, cDNA was synthesized from 1 μg RNA using iScript Reverse Transcription Supermix for RT-qPCR (Bio-Rad). Subsequent measurements were performed on the CFX Connect Real-Time PCR Detection System (Bio-Rad) using iTaq Universal SYBR Green Supermix (Bio-Rad), following the manufacturer's protocol. The 20 μl reaction contained 1 μl cDNA, 10 μl (2 ×) supermix and 0.5 μM of each primer (Supplementary Table 7). Samples were initially denatured at 95 °C for 30 s, run for 40 cycles at 95 °C for 5 s and 59 °C for 30 s. Data were recorded after the annealing/extension step. The specificity of the amplification product was verified by melt curves and running the amplification products on an agarose gel. Pooled cDNA of all time points were used in a serial dilution to determine the efficiency of amplification for each primer pair. On the basis of the *C*_q_ values obtained during the efficiency tests, 1 μl of 1:50 dilution of the original cDNA of each time point was used as a template for the subsequent expression analysis. *Actin* and *Histone H2A* served as reference genes. The non-treated sample (0 h) served as a calibrator. Amplification efficiency as well as normalized expression relative to the calibrator were determined using the Bio-Rad CFX Manager software (version 3.1, Bio-Rad). The presented data are the means of three technical replicates, error bars representing s.e.m.

### C-terminal exchanges

The reciprocal exchange of the terminal 55 amino acids between HpCYP81AA1 and HpCYP81AA2 was performed using the one-pot fusion PCR protocol as previously described^55^. CYP81AA1 with the C-terminus of CYP81AA2 (HpCYP81AA1-AA2) was generated in a 50 μl PCR reaction, which contained 500 ng of each template plasmid (pESC-URA:HpCYP81AA1/HcCPR2 and pESC-URA:HpCYP81AA2/HcCPR2) in 1 × Phusion HF buffer, 0.2 μM primers (Hp81AA1-*Spe*I-F and Hp81AA2-*Pac*I-R) (Supplementary Table 7), 0.04 μM overlapping primer (AA1-AA2-ex) (Supplementary Table 8), 0.2 mM dNTPs and 1U Phusion Hot Start II High-Fidelity DNA Polymerase (Thermo Scientific). The PCR started with an initial denaturation at 98 °C for 30 s, thereafter 35 cycles at 98 °C for 10 s, 60 °C for 30 s and 72 °C for 90 s, followed by a final extension at 72 °C for 10 min. The PCR product with the expected size (∼1,500 bp) was excised from an agarose gel stained with Midori Green (Nippon Genetics), purified using the innuPREP DOUBLEpure Kit (Analytic Jena), digested with *Spe*I and *Pac*I restriction enzymes (Thermo Scientific), ligated to the pESC-URA:HcCPR2 plasmid previously digested with the same restriction enzymes, transformed into DH5α and sequenced. Similarly, CYP81AA2 with the C-terminus of CYP81AA1 (HpCYP81AA2-AA1) was generated using the same reaction conditions, except for using the primers Hp81AA2-*Spe*I-F, Hp81AA1-*Pac*I-R and AA2-AA1-ex (Supplementary Tables 7 and 8).

### Site-directed mutagenesis

Partially overlapping complementary primers were designed according to the method described previously^56^. The 50 μl PCR reactions were set up with 25–50 ng template plasmid in 1 × Phusion HF buffer, 0.2 mM dNTPs, 0.5 μM primers (Supplementary Table 8) and 1 U Phusion Hot Start II High-Fidelity DNA Polymerase (Thermo Scientific). After initial denaturation at 98 °C for 30 s, samples were run for 25 cycles at 98 °C for 10 s, 70 °C for 30 s and 72 °C for 6:30 min, followed by a final extension at 72 °C for 10 min. *Dpn*I (10 U) was added to the product and incubated at 37 °C for 3 h to digest the template plasmid. An aliquot of 5 μl of the digestion mixture was directly used to transform DH5α. Plasmids were isolated and sequenced to confirm the introduction of the designed mutations. The templates and primers used to generate the individual mutants are listed in Supplementary Table 9.

### CYP expression and preparation of yeast microsomes

The constructed plasmids and the plasmids obtained after mutagenesis were transferred to the *S. cerevisiae* strain INV*Sc*1 using the *S.c.* EasyComp Kit (Invitrogen), following the manufacturer's protocol. The yeast cells were grown and gene expression was performed as previously described^57^. The transformation mixture was spread on solid synthetic dextrose (s.d.) minimal medium (0.67% yeast nitrogen base, 2% D-dextrose, supplemented with amino acids but omitting uracil) for auxotrophic selection and incubated at 30 °C for 48 h. A single colony was inoculated in a 5 ml s.d. tube and incubated at 30 °C with shaking at 250 r.p.m. for 24 h. An aliquot (1 ml) of the resulting culture was transferred to 150 ml YPGE medium (1% yeast extract, 1% peptone, 0.5% glucose and 3% ethanol) and grown for 28–30 h until OD_600_ reached 1.5–1.7. Expression was induced by the addition of 2% galactose and the culture was further incubated for 14–16 h. Cells were harvested by centrifugation, washed twice with 10 and 5 ml of TEK buffer (50 mM Tris-HCl pH 7.4, 1 mM EDTA and 0.1 M KCl), manually broken with 3 g of 0.45 mm glass beads in 3 ml of TES-B buffer (50 mM Tris-HCl pH 7.5, 1 mM EDTA and 600 mM sorbitol) and centrifuged at 3840 *g* for 5 min. The supernatant was collected and the pellet was further washed twice with 3 ml of TES-B buffer each. The combined homogenate was centrifuged for 10 min at 25 000 *g* to pellet the mitochondria and nuclei. The supernatant was centrifuged for 1 h at 100 000 *g* to pellet the microsomal membranes. Microsomes were resuspended in 1 ml of TEG buffer (50 mM Tris-HCl pH 7.4, 1 mM EDTA and 20% glycerol) and stored at −80 °C. All steps for microsomal preparation were performed at 0–4 °C.

### Enzyme assays

Enzymatic activities were determined in a standard 200 μl assay containing 50 μg microsomal protein, 1 mM NADPH and 0.2 mM substrate in 100 mM sodium phosphate buffer (pH 7.0). The reaction was initiated by the addition of NADPH, incubated at 30 °C for 1 h and stopped by the addition of 20 μl 1N HCl. After extraction twice with 250 μl ethyl acetate, the combined organic phase was evaporated to dryness, dissolved in 60 μl methanol, 25 μl of which were analysed by HPLC.

### HPLC analysis

HPLC was carried out on a VWR-Hitachi LaChrom Elite system (pump L-2130, autosampler L-2200, diode array detector L-2455), equipped with a ZORBAX Eclipse Plus column (C18, 4.6 × 100 mm, 3.5 μ; Agilent). The solvents used were 0.1% formic acid in water (A) and acetonitrile (B). The gradients were as follows. Incubations with pentahydroxybenzophenone and tetrahydroxyxanthones: 4 min 5% B, 12 min 20% B, 25 min 27% B and 28 min 100% B; incubations with trihydroxybenzophenone, tetrahydroxybenzophenone and trihydroxyxanthones: 4 min 5% B, 12 min 30% B, 25 min 34% B and 28 min 100% B; and incubations with mono- and dihydroxybenzophenones and dihydroxyxanthones: 4 min 5% B, 10 min 30% B, 22 min 40% B and 26 min 100% B. The flow rate was 1 ml min^−1^ and the detection wavelength was 260 nm.

### LC-MS analysis

Products of 30 enzymatic incubations were purified by HPLC and corresponding peaks were collected as individual fractions. These fractions were directly infused into the mass spectrometer (3200 QTrap mass spectrometer; Applied Biosystems/MDS SCIEX, Darmstadt, Germany), equipped with an electrospray ionization interface (Turbo V), using the integrated syringe pump of the 3200 QTrap instrument (Syringe; 1,000 μl, i.d. 2.3 mm; Hamilton, Nevada, USA) at a flow rate of 10 μl min^−1^. The MS/MS was operated in the positive mode with a source voltage and declustering potential of 5.5 kV and 76 V, respectively. Nitrogen gas was used for nebulization, with the curtain gas, gas 1, and gas 2 settings at 10, 14 and 0, respectively. Parameters were optimized for benzophenones using standard 2,4,6-trihydroxybenzophenone and for xanthones using 1,3,7-trihydroxyxanthone in methanol at 10 μg ml^−1^. The molecular ion peaks [M+H]^+^ of the products were further analysed by MS/MS experiments in the enhanced product ion (EPI) mode of the instrument using nitrogen gas for collision-induced dissociation at the high-level setting. The collision energy was 30–50 V. Data acquisition and processing were performed using the Analyst software (version 1.4.2; Applied Biosystems/MDS SCIEX).

### Secondary structures prediction and standard numbering

The deduced amino acid sequences of Hc/HpCYP81AA1 and HpCYP81AA2 were aligned with ClustalW2 using Gonnet protein weight matrix, a gap opening penalty of 10 and a gap extension penalty of 0.2. The secondary structures of HpCYP81AA1 and HpCYP81AA2 were predicted using the CYP modules prediction tool in the Cytochrome P450 Engineering Database (CYPED)^32^. The standard residues positions were also obtained by blasting the sequences in the CYPED website (https://cyped.biocatnet.de/workbench/numbering)^44^.

### Homology modelling

The HHpred server^36^ was used to identify homologous CYP X-ray crystal structures as three-dimensional templates for comparative modelling utilizing the software Modeler^58^. The identified homologous template structures were all in the low sequence identity range (between 20–25% sequence identity) and an alternative homology modelling attempt using YASARA Structure 14.7.17 was used to identify the most suitable template structures by another independent method^59^,^60^. Structures from two matching CYP-families were identified by sequence search using Psi-BLAST^61^ and Psi-Pred secondary structure prediction employing position-specific patterns^62^. The generated homology models were refined using MD-simulations with the YAMBER^63^ force field and ranked according to knowledge-based structural descriptors as implemented in YASARA. The best scoring structures were obtained using the X-ray crystal structure from eukaryotic CYP2B4^37^ in the closed conformation with the bound inhibitor ticlopidine (PDB: 3kw4) as template. In the alignment, 400 out of 508 target residues (78.7%) are aligned to template residues of HpCYP81AA1. Among these aligned residues, the sequence identity is 30.8% and the sequence similarity is 48.0% ('similar' means that the BLOSUM62 score is>0). A second reference structure showing good scores was human CYP17A1 (steroid 17-α-hydroxylase) in closed conformation with bound substrate mimetic. In the alignment, 378 out of 508 target residues (74.4%) are aligned to template residues of HpCYP81AA1. Among these aligned residues, the sequence identity was 27.8% and the sequence similarity was 47.4% (‘similar' means that the BLOSUM62 score is>0). This model structure based on CYP17A1 showed a different orientation of the C-terminal β-loop (SRS-6) and the adjacent α-helix containing SRS-2 and it was crystallized in the dimeric form. The final three-dimensional-models of HpCYP81AA1 and HpCYP81AA2 were generated from a hybrid model using the HHpred server and the three best matching template structures: 3kw4 (CYP2B4), 3swz (CYP17A1), 3qz1 (steroid 21-hydroxylase) and 2hi4 (CYP1A2). The best scoring model was built on eukaryotic CYP2B4 (ref. 37) in the closed conformation with the bound inhibitor ticlopidine (PDB: 3kw4) as template.

### Product docking

Molecular docking was carried out using AutoDock Vina^64^. Twenty-five and 100 docking runs were performed for rigid protein side chains and flexible side chains in the binding pocket, respectively (Supplementary Data 1). The docking solutions were clustered by applying a RMSD cutoff of 5 Å. The standard settings within the provided YASARA dock run and dock ensemble macros were used. The overlaid structures and the results are depicted in the Supplementary Fig. 12. Product docking identified several binding modes indicating 3′-hydroxylation; however, no catalytic-binding orientations for the subsequent cyclization (cyclized bond <6 Å from catalytic haem iron) were obtained when automated docking with rigid protein backbone was employed. Therefore, mechanism-guided manual building of the product in a reactive pose was used, which allowed for full relaxation of the backbone and the side chains of the substrate binding loops in the pocket, based on the below-described staggered minimization protocol. This procedure consisted of steepest descent and simulated annealing minimization steps using fully flexible AMBER molecular dynamics and including several heating/freezing cycles from 298 to 10 K. To this end, the model structures of HpCYP81AA1 and HpCYP81AA2 were overlaid by MUSTANG^65^ with haem and the ticlopidine inhibitor-bound structure (PDB: 3kw4) using YASARA Structure Ver. 14.7.17. The initial binding modes of the respective products (1,3,5-triHX and 1,3,7-triHX) were introduced into the apo-model structures using catalytic constraints, so that the cyclized bond and the respective carbon that undergoes H-abstraction are in a distance below 6 Å from the catalytic haem iron. On the basis of this initial product bound model, the binding mode was manually built and energy minimized using YASARA^66^. To remove bumps and correct the covalent geometry, first a short steepest descent minimization was performed. After removal of conformational stress, the procedure was continued by simulated annealing (timestep 2 fs, atom velocities scaled down by 0.9 every 10th step) until convergence was reached, that is, the energy improved by less than 0.05 kJ mol^−1^ per atom during 200 steps using the AMBER03 (ref. 67) force field for protein residues and the general AMBER force field GAFF (ref. 68). AM1BCC (ref. 69) calculated partial charges and a force cutoff of 0.786 Å and particle mesh Ewald for exact treatment of long range electrostatics by periodic boundary conditions were employed. The same procedure was applied to the active site mutation S375A/L378S/A483T/M486L/K488R/N489K (mut6).

## Additional information

**Accession codes:** The sequences of the genes cloned in this work were deposited in the NCBI GenBank under the following accession codes: HcCYP81AA1 (KT716863), HpCYP81AA1 (KT716864), HpCYP81AA2 (KT716865), HpCYP81AA3 (KT716866), HcCPR2 (KT777456).

**How to cite this article:** El-Awaad, I. *et al*. Bifunctional CYP81AA proteins catalyse identical hydroxylations but alternative regioselective phenol couplings in plant xanthone biosynthesis. *Nat. Commun.* 7:11472 doi: 10.1038/ncomms11472 (2016).

## Supplementary Material

Supplementary Information Supplementary Figures 1-12, Supplementary Tables 1-9, Supplementary Methods and Supplementary References

Supplementary Data 1 Product docking

## Figures and Tables

**Figure 1:**
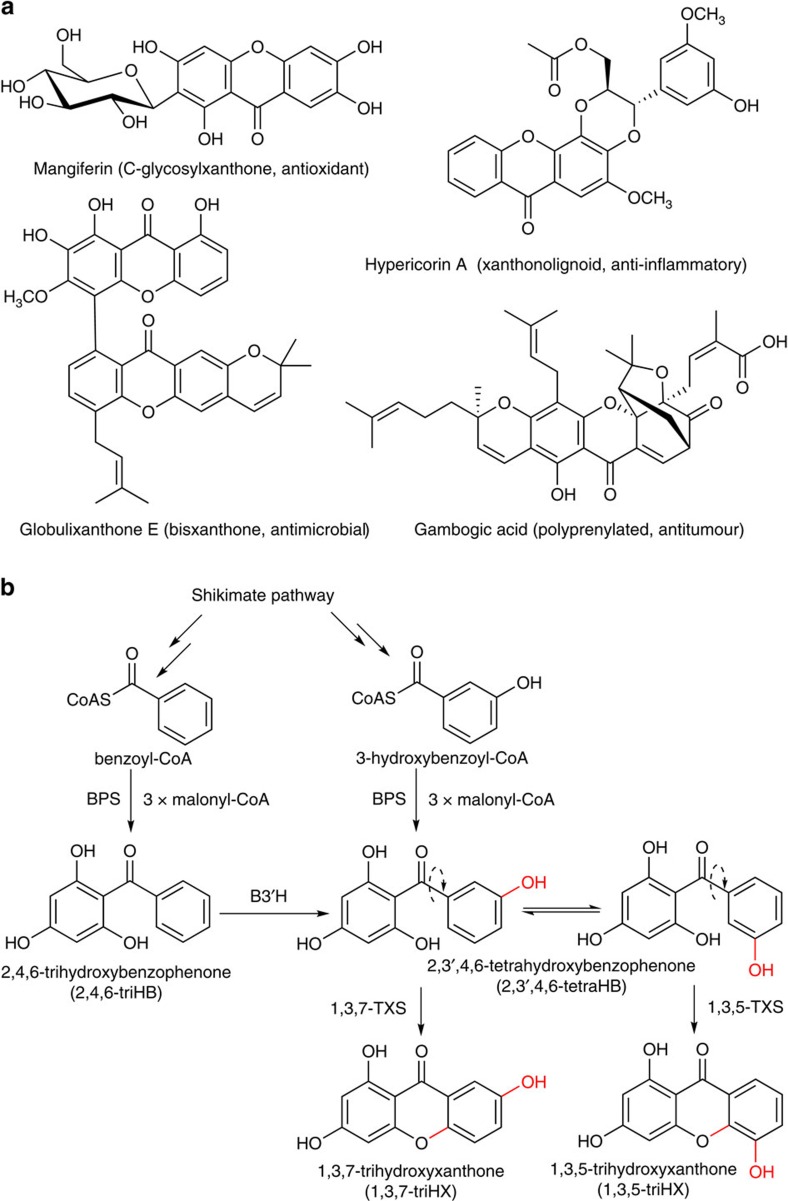
Selected classes and biosynthesis of plant xanthones. (**a**) Examples of xanthone-based compounds and their biological activities. (**b**) Proposed biosynthesis of 1,3,7- and 1,3,5-triHXs. BPS, benzophenone synthase; B3′H, benzophenone 3′-hydroxylase; TXS, trihydroxyxanthone synthase.

## References

[b1] Nelson D. & Werck-Reichhart D. A P450-centric view of plant evolution. Plant J. 66, 194–211 (2011). 21443632 10.1111/j.1365-313X.2011.04529.x

[b2] Mizutani M. & Sato F. Unusual P450 reactions in plant secondary metabolism. Arch. Biochem. Biophys. 507, 194–203 (2011). 20920462 10.1016/j.abb.2010.09.026

[b3] Guengerich F. P. & Munro A. W. Unusual cytochrome P450 enzymes and reactions. J. Biol. Chem. 288, 17065–17073 (2013). 23632016 10.1074/jbc.R113.462275 PMC3682512

[b4] Zhang Y. . Rice cytochrome P450 MAX1 homologs catalyze distinct steps in strigolactone biosynthesis. Nat. Chem. Biol. 10, 1028–1033 (2014). 25344813 10.1038/nchembio.1660

[b5] Ono E. . Formation of two methylenedioxy bridges by a *Sesamum* CYP81Q protein yielding a furofuran lignan, (+)-sesamin. Proc. Natl Acad. Sci. USA 103, 10116–10121 (2006). 16785429 10.1073/pnas.0603865103 PMC1502515

[b6] Klein A. P. & Sattely E. S. Two cytochromes P450 catalyze S-heterocyclizations in cabbage phytoalexin biosynthesis. Nat. Chem. Biol. 11, 837–839 (2015). 26389737 10.1038/nchembio.1914 PMC4731101

[b7] Gesell A. . CYP719B1 is salutaridine synthase, the C–C phenol-coupling enzyme of morphine biosynthesis in opium poppy. J. Biol. Chem. 284, 24432–24442 (2009). 19567876 10.1074/jbc.M109.033373 PMC2782036

[b8] Ikezawa N., Iwasa K. & Sato F. Molecular cloning and characterization of CYP80G2, a cytochrome P450 that catalyzes an intramolecular C–C phenol coupling of (*S*)-reticuline in magnoflorine biosynthesis, from cultured *Coptis japonica* cells. J. Biol. Chem. 283, 8810–8821 (2008). 18230623 10.1074/jbc.M705082200

[b9] Kraus P. F. & Kutchan T. M. Molecular cloning and heterologous expression of a cDNA encoding berbamunine synthase, a C–O phenol-coupling cytochrome P450 from the higher plant *Berberis stolonifera*. Proc. Natl Acad. Sci. USA 92, 2071–2075 (1995). 7892226 10.1073/pnas.92.6.2071 PMC42425

[b10] Peters S., Schmidt W. & Beerhues L. Regioselective oxidative phenol couplings of 2,3′,4,6-tetrahydroxybenzophenone in cell cultures of *Centaurium erythraea* RAFN and *Hypericum androsaemum* L. Planta 204, 64–69 (1997).

[b11] El-Seedi H. R. . Recent insights into the biosynthesis and biological activities of natural xanthones. Curr. Med. Chem. 17, 854–901 (2010). 20156171 10.2174/092986710790712147

[b12] Crockett S. L. & Robson N. K. Taxonomy and chemotaxonomy of the genus *Hypericum*. Med. Aromat. Plant Sci. Biotechnol. 5, 1–13 (2011). 22662019 PMC3364714

[b13] Wezeman T., Bräse S. & Masters K. S. Xanthone dimers: a compound family which is both common and privileged. Nat. Prod. Rep. 32, 6–28 (2015). 25226564 10.1039/c4np00050a

[b14] Tocci N. . Root cultures of *Hypericum perforatum* subsp. *angustifolium* elicited with chitosan and production of xanthone-rich extracts with antifungal activity. Appl. Microbiol. Biotechnol. 91, 977–987 (2011). 21547455 10.1007/s00253-011-3303-6

[b15] Wang Y. . α-Mangostin, a polyphenolic xanthone derivative from mangosteen, attenuates β-amyloid oligomers-induced neurotoxicity by inhibiting amyloid aggregation. Neuropharmacology 62, 871–881 (2012). 21958557 10.1016/j.neuropharm.2011.09.016

[b16] Masters K. S. & Bräse S. Xanthones from fungi, lichens, and bacteria: the natural products and their synthesis. Chem. Rev. 112, 3717–3776 (2012). 22617028 10.1021/cr100446h

[b17] Liu B., Falkenstein-Paul H., Schmidt W. & Beerhues L. Benzophenone synthase and chalcone synthase from *Hypericum androsaemum* cell cultures: cDNA cloning, functional expression, and site-directed mutagenesis of two polyketide synthases. Plant J. 34, 847–855 (2003). 12795704 10.1046/j.1365-313x.2003.01771.x

[b18] Schmidt W. & Beerhues L. Alternative pathways of xanthone biosynthesis in cell cultures of *Hypericum androsaemum* L. FEBS Lett. 420, 143–146 (1997). 9459298 10.1016/s0014-5793(97)01507-x

[b19] Zhang H.-Z. . Discovery, characterization and SAR of gambogic acid as a potent apoptosis inducer by a HTS assay. Bioorg. Med. Chem. 12, 309–317 (2004). 14723951 10.1016/j.bmc.2003.11.013

[b20] Gaid M. M. . Cinnamate:CoA ligase initiates the biosynthesis of a benzoate-derived xanthone phytoalexin in *Hypericum calycinum* cell cultures. Plant Physiol. 160, 1267–1280 (2012). 22992510 10.1104/pp.112.204180 PMC3490583

[b21] Nelson D. R., Schuler M. A., Paquette S. M., Werck-Reichhart D. & Bak S. Comparative genomics of rice and *Arabidopsis*. Analysis of 727 cytochrome P450 genes and pseudogenes from a monocot and a dicot. Plant Physiol. 135, 756–772 (2004). 15208422 10.1104/pp.104.039826 PMC514113

[b22] Nelson D. R. Cytochrome P450 nomenclature, 2004. Methods Mol. Biol. 320, 1–10 (2006). 16719369 10.1385/1-59259-998-2:1

[b23] Werck-Reichhart D. & Feyereisen R. Cytochromes P450: a success story. Genome Biol. 1, REVIEWS3003 (2000). 11178272 10.1186/gb-2000-1-6-reviews3003 PMC138896

[b24] Mizutani M. & Ohta D. Two isoforms of NADPH:cytochrome P450 reductase in *Arabidopsis thaliana*. Gene structure, heterologous expression in insect cells, and differential regulation. Plant physiol. 116, 357–367 (1998). 9449848 10.1104/pp.116.1.357 PMC35176

[b25] Yang C. Q., Lu S., Mao Y. B., Wang L. J. & Chen X. Y. Characterization of two NADPH: cytochrome P450 reductases from cotton (*Gossypium hirsutum*). Phytochemistry 71, 27–35 (2010). 19883924 10.1016/j.phytochem.2009.09.026

[b26] Ro D.-K., Ehlting J. & Douglas C. J. Cloning, functional expression, and subcellular localization of multiple NADPH-cytochrome P450 reductases from hybrid poplar. Plant Physiol. 130, 1837–1851 (2002). 12481067 10.1104/pp.008011 PMC166695

[b27] Porter T. D., Beck T. W. & Kasper C. B. NADPH-cytochrome P-450 oxidoreductase gene organization correlates with structural domains of the protein. Biochemistry 29, 9814–9818 (1990). 2125483 10.1021/bi00494a009

[b28] Urban P., Mignotte C., Kazmaier M., Delorme F. & Pompon D. Cloning, yeast expression, and characterization of the coupling of two distantly related *Arabidopsis thaliana* NADPH-cytochrome P450 reductases with P450 CYP73A5. J. Biol. Chem. 272, 19176–19186 (1997). 9235908 10.1074/jbc.272.31.19176

[b29] Guengerich F. P., Martin M. V., Sohl C. D. & Cheng Q. Measurement of cytochrome P450 and NADPH-cytochrome P450 reductase. Nat. Protoc. 4, 1245–1251 (2009). 19661994 10.1038/nprot.2009.121 PMC3843963

[b30] Jefferson A. & Scheinmann F. Presence of 1,3,6,7-tetrahydroxyxanthone in maclurin from *Chlorophora tinctoria* (L) Gaud. (*Morus tinctoria* L) (Moraceae). Nature 207, 1193–1193 (1965).

[b31] Crockett S. L. . Bioactive xanthones from the roots of *Hypericum perforatum* (common St John's wort). J. Sci. Food Agric. 91, 428–434 (2011). 21218475 10.1002/jsfa.4202 PMC3318991

[b32] Sirim D., Widmann M., Wagner F. & Pleiss J. Prediction and analysis of the modular structure of cytochrome P450 monooxygenases. BMC Struct. Biol. 10, 34 (2010). 20950472 10.1186/1472-6807-10-34 PMC3224734

[b33] Gricman L., Vogel C. & Pleiss J. Conservation analysis of class-specific positions in cytochrome P450 monooxygenases: functional and structural relevance. Proteins 82, 491–504 (2014). 24105833 10.1002/prot.24415

[b34] Gotoh O. Substrate recognition sites in cytochrome P450 family 2 (CYP2) proteins inferred from comparative analyses of amino acid and coding nucleotide sequences. J. Biol. Chem. 267, 83–90 (1992). 1730627

[b35] Seifert A. & Pleiss J. Identification of selectivity-determining residues in cytochrome P450 monooxygenases: a systematic analysis of the substrate recognition site 5. Proteins 74, 1028–1035 (2009). 18814300 10.1002/prot.22242

[b36] Söding J., Biegert A. & Lupas A. N. The HHpred interactive server for protein homology detection and structure prediction. Nucleic Acids Res. 33, W244–W248 (2005). 15980461 10.1093/nar/gki408 PMC1160169

[b37] Gay S. C. . Structures of cytochrome P450 2B4 complexed with the antiplatelet drugs ticlopidine and clopidogrel. Biochemistry 49, 8709–8720 (2010). 20815363 10.1021/bi100914z PMC3005796

[b38] Munro A. W., Girvan H. M., Mason A. E., Dunford A. J. & McLean K. J. What makes a P450 tick? Trends Biochem. Sci. 38, 140–150 (2013). 23356956 10.1016/j.tibs.2012.11.006

[b39] Rittle J. & Green M. T. Cytochrome P450 compound I: capture, characterization, and C–H bond activation kinetics. Science 330, 933–937 (2010). 21071661 10.1126/science.1193478

[b40] Gerardy R. & Zenk M. H. Formation of salutaridine from (*R*)-reticuline by a membrane-bound cytochrome P-450 enzyme from *Papaver somniferum*. Phytochemistry 32, 79–86 (1992).

[b41] Belin P. . Identification and structural basis of the reaction catalyzed by CYP121, an essential cytochrome P450 in *Mycobacterium tuberculosis*. Proc. Natl Acad. Sci. USA 106, 7426–7431 (2009). 19416919 10.1073/pnas.0812191106 PMC2678619

[b42] Grobe N. . Mammalian cytochrome P450 enzymes catalyze the phenol-coupling step in endogenous morphine biosynthesis. J. Biol. Chem. 284, 24425–24431 (2009). 19561069 10.1074/jbc.M109.011320 PMC2782035

[b43] Kitanov G. M. & Nedialkov P. T. Benzophenone *O*-glucoside, a biogenic precursor of 1,3,7-trioxygenated xanthones in *Hypericum annulatum*. Phytochemistry 57, 1237–1243 (2001). 11454351 10.1016/s0031-9422(01)00194-7

[b44] Gricman L., Vogel C. & Pleiss J. Identification of universal selectivity-determining positions in cytochrome P450 monooxygenases by systematic sequence-based literature mining. Proteins 83, 1593–1603 (2015). 26033392 10.1002/prot.24840

[b45] Schalk M. & Croteau R. A single amino acid substitution (F363I) converts the regiochemistry of the spearmint (-)-limonene hydroxylase from a C6- to a C3-hydroxylase. Proc. Natl Acad. Sci. USA 97, 11948–11953 (2000). 11050228 10.1073/pnas.97.22.11948 PMC17275

[b46] Kahn R. A., Bouquin R. L., Pinot F., Benveniste I. & Durst F. A conservative amino acid substitution alters the regiospecificity of CYP94A2, a fatty acid hydroxylase from the plant *Vicia sativa*. Arch. Biochem. Biophys. 391, 180–187 (2001). 11437349 10.1006/abbi.2001.2415

[b47] Bak S. . Cytochromes P450. Arabidopsis Book 9, e0144 (2011). 22303269 10.1199/tab.0144 PMC3268508

[b48] Nelson D. R., Ming R., Alam M. & Schuler M. A. Comparison of cytochrome P450 genes from six plant genomes. Trop. Plant Biol. 1, 216–235 (2008).

[b49] Pfalz M., Vogel H. & Kroymann J. The gene controlling the *indole glucosinolate modifier1* quantitative trait locus alters indole glucosinolate structures and aphid resistance in *Arabidopsis*. Plant Cell 21, 985–999 (2009). 19293369 10.1105/tpc.108.063115 PMC2671713

[b50] Liu C.-J., Huhman D., Sumner L. W. & Dixon R. A. Regiospecific hydroxylation of isoflavones by cytochrome P450 81E enzymes from *Medicago truncatula*. Plant J. 36, 471–484 (2003). 14617078 10.1046/j.1365-313x.2003.01893.x

[b51] Haslinger K., Peschke M., Brieke C., Maximowitsch E. & Cryle M. J. X-domain of peptide synthetases recruits oxygenases crucial for glycopeptide biosynthesis. Nature 521, 105–109 (2015). 25686610 10.1038/nature14141

[b52] Guengerich F. P. Mechanisms of cytochrome P450 substrate oxidation: MiniReview. J. Biochem. Mol. Toxicol. 21, 163–168 (2007). 17936929 10.1002/jbt.20174

[b53] Huang X. & Madan A. CAP3: A DNA sequence assembly program. Genome Res. 9, 868–877 (1999). 10508846 10.1101/gr.9.9.868 PMC310812

[b54] Conesa A. . Blast2GO: a universal tool for annotation, visualization and analysis in functional genomics research. Bioinformatics 21, 3674–3676 (2005). 16081474 10.1093/bioinformatics/bti610

[b55] Büttner M. & Barleben L. One-pot fusion polymerase chain reaction for combinatorial synthesis of DNA from several cassettes. Anal. Biochem. 421, 797–798 (2012). 22230284 10.1016/j.ab.2011.12.027

[b56] Liu H. & Naismith J. H. An efficient one-step site-directed deletion, insertion, single and multiple-site plasmid mutagenesis protocol. BMC Biotechnol. 8, 91 (2008). 19055817 10.1186/1472-6750-8-91 PMC2629768

[b57] Pompon D., Louerat B., Bronine A. & Urban P. Yeast expression of animal and plant P450s in optimized redox environments. Methods Enzymol. 272, 51–64 (1996). 8791762 10.1016/s0076-6879(96)72008-6

[b58] Sali A. & Blundell T. L. Comparative protein modelling by satisfaction of spatial restraints. J. Mol. Biol. 234, 779–815 (1993). 8254673 10.1006/jmbi.1993.1626

[b59] Krieger E., Nabuurs S. B. & Vriend G. Homology modeling. Methods Biochem. Anal. 44, 509–523 (2003). 12647402 10.1002/0471721204.ch25

[b60] Krieger E. & Vriend G. YASARA View - molecular graphics for all devices - from smartphones to workstations. Bioinformatics 30, 2981–2982 (2014). 24996895 10.1093/bioinformatics/btu426 PMC4184264

[b61] Altschul S. F. . Gapped BLAST and PSI-BLAST: a new generation of protein database search programs. Nucleic Acids Res. 25, 3389–3402 (1997). 9254694 10.1093/nar/25.17.3389 PMC146917

[b62] Jones D. T. Protein secondary structure prediction based on position-specific scoring matrices. J. Mol. Biol. 292, 195–202 (1999). 10493868 10.1006/jmbi.1999.3091

[b63] Krieger E. . Improving physical realism, stereochemistry, and side-chain accuracy in homology modeling: Four approaches that performed well in CASP8. Proteins 77 (suppl 9): 114–122 (2009). 19768677 10.1002/prot.22570 PMC2922016

[b64] Trott O. & Olson A. J. AutoDock Vina: improving the speed and accuracy of docking with a new scoring function, efficient optimization, and multithreading. J. Comput. Chem. 31, 455–461 (2010). 19499576 10.1002/jcc.21334 PMC3041641

[b65] Konagurthu A. S., Whisstock J. C., Stuckey P. J. & Lesk A. M. MUSTANG: A multiple structural alignment algorithm. Proteins 64, 559–574 (2006). 16736488 10.1002/prot.20921

[b66] Krieger E., Darden T., Nabuurs S. B., Finkelstein A. & Vriend G. Making optimal use of empirical energy functions: force-field parameterization in crystal space. Proteins 57, 678–683 (2004). 15390263 10.1002/prot.20251

[b67] Duan Y. . A point-charge force field for molecular mechanics simulations of proteins based on condensed-phase quantum mechanical calculations. J. Comput. Chem. 24, 1999–2012 (2003). 14531054 10.1002/jcc.10349

[b68] Wang J., Wolf R. M., Caldwell J. W., Kollman P. A. & Case D. A. Development and testing of a general amber force field. J. Comput. Chem. 25, 1157–1174 (2004). 15116359 10.1002/jcc.20035

[b69] Jakalian A., Jack D. B. & Bayly C. I. Fast, efficient generation of high-quality atomic charges. AM1-BCC model: II. Parameterization and validation. J. Comput. Chem. 23, 1623–1641 (2002). 12395429 10.1002/jcc.10128

