# Supplementary material for: Bifunctional CYP81AA proteins catalyse identical hydroxylations but alternative regioselective phenol couplings in plant xanthone biosynthesis
Source: Nat Commun. 2016 May 5;7:11472. doi: 10.1038/ncomms11472 (PMC4858744; doi:10.1038/ncomms11472)
Supplement: Supplementary Information — Supplementary Figures 1-12, Supplementary Tables 1-9, Supplementary Methods and Supplementary References [file ncomms11472-s1.pdf]

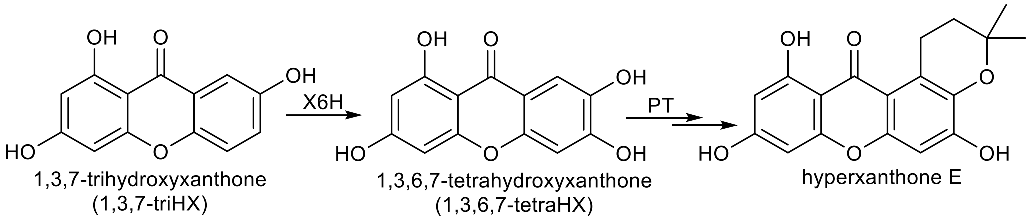

Supplementary Figure 1 | Proposed biosynthesis of hyperxanthone E from 1,3,7-triHX in elicitor-treated *H. calycinum* cell cultures. X6H, xanthone 6-hydroxylase; PT, prenyltransferase.

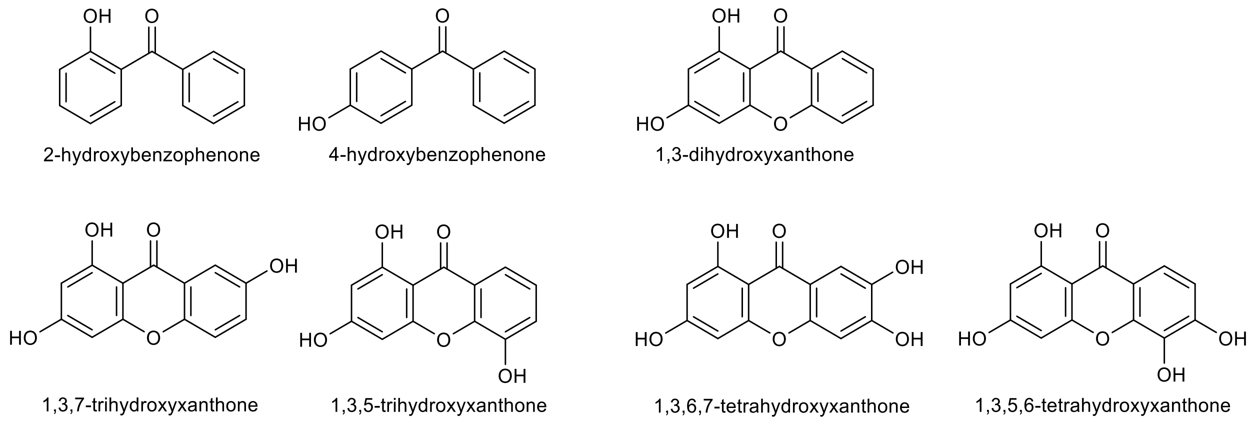

Supplementary Figure 2 | Substrates not accepted by CYP81AA1 and CYP81AA2.

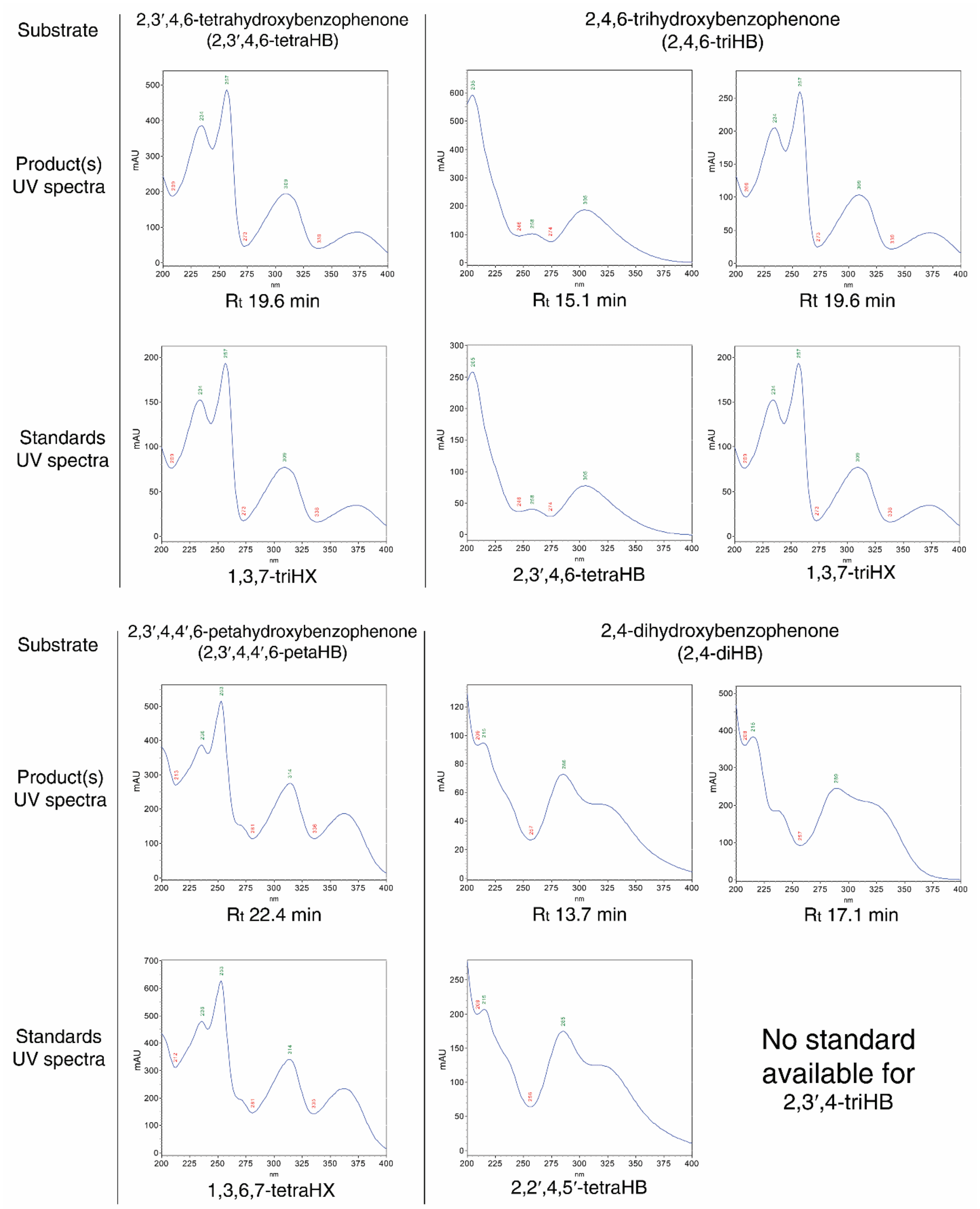

Supplementary Figure 3 | UV spectra of CYP81AA1 products and authentic compounds.

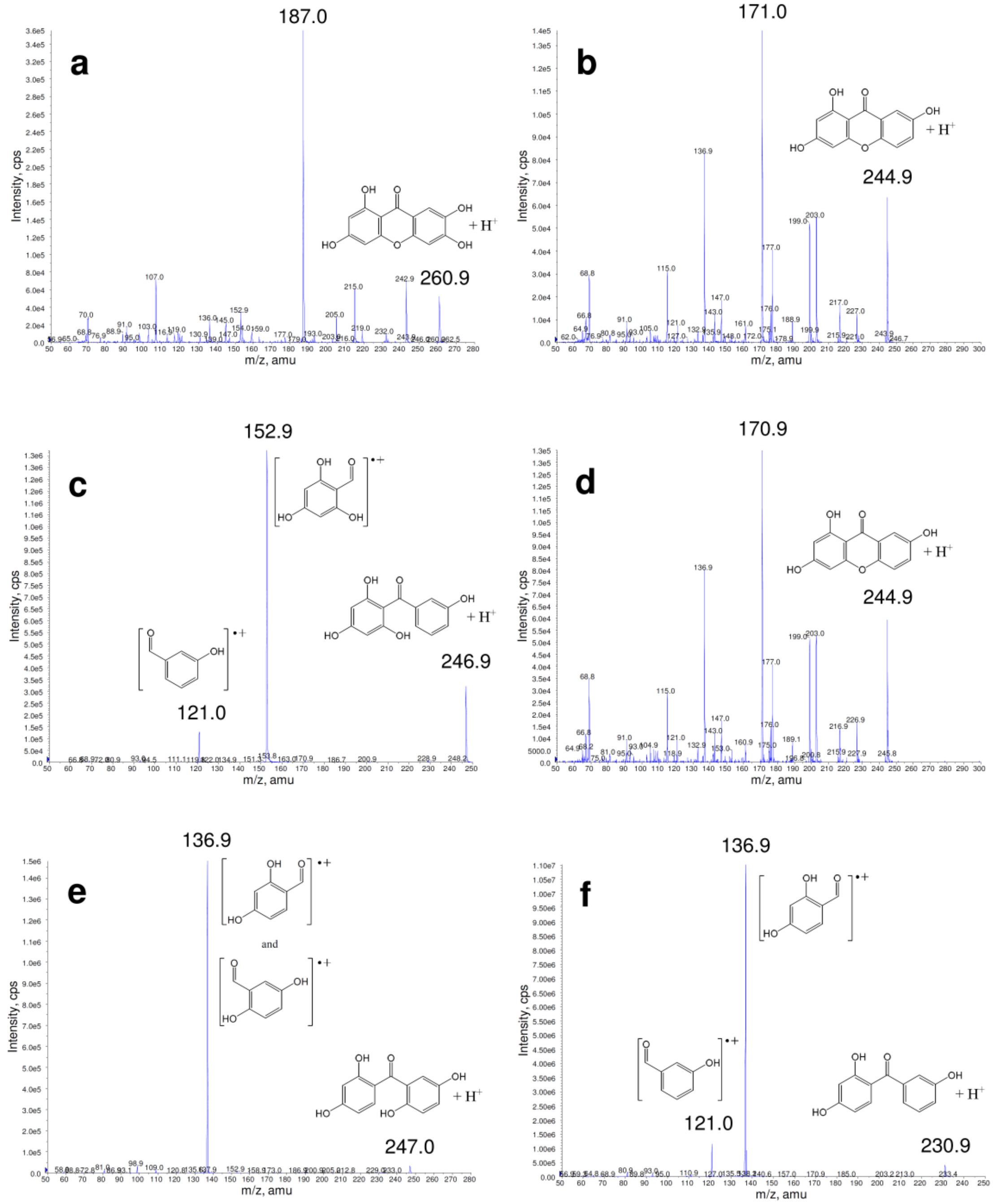

Supplementary Figure 4 | Enhanced product ion (EPI) mass spectra of CYP81AA1 products formed from various substrates. (**a**) 1,3,6,7-tetraHX (R_t = 22.3 min) from 2,3′,4,4′,6-pentaHB. (**b**) 1,3,7-triHX (R_t = 19.6 min) from 2,3′,4,6-tetraHB. (**c**) 2,3′,4,6-tetraHB (R_t = 15.1 min) from 2,4,6-triHB. (**d**) 1,3,7-triHX (R_t = 19.6 min) from 2,4,6-triHB. (**e**) 2,2′,4,5′-tetraHB (R_t = 13.7 min) from 2,4-diHB. (**f**) 2,3′,4-triHB (R_t = 17.1 min) from 2,4-diHB.

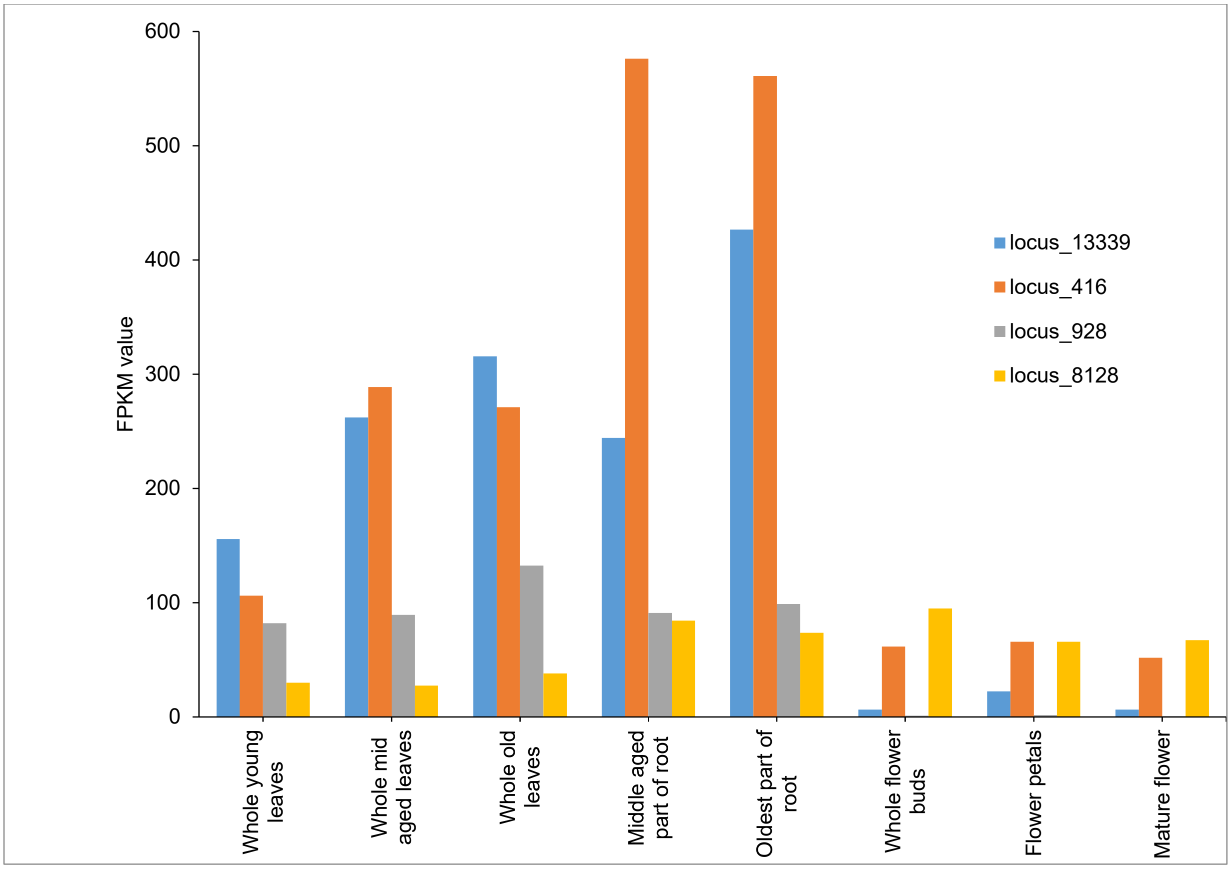

Supplementary Figure 5 | Tissue distribution of transcripts encoded by the loci 13339 (*HpBPS*), 416 (*HpCYP81AA1*), 928 (*HpCYP81AA2*), and 8128 (*HpCYP81AA3*). Data are taken from the MPGR database (http://medicinalplantgenomics.msu.edu). FPKM, fragments per kilobase per million mapped fragments.

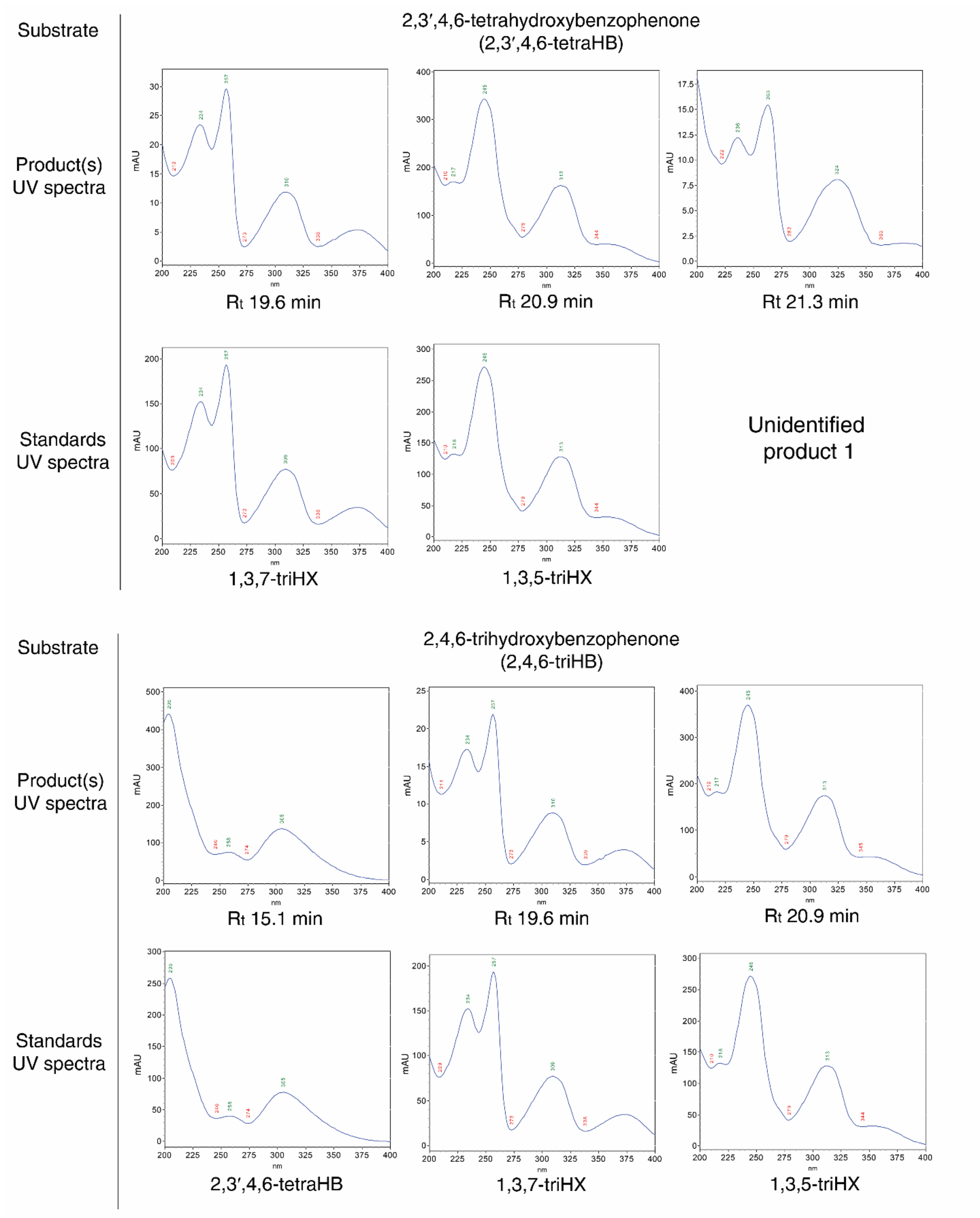

Supplementary Figure 6 | UV spectra of CYP81AA2 products and authentic compounds (I).

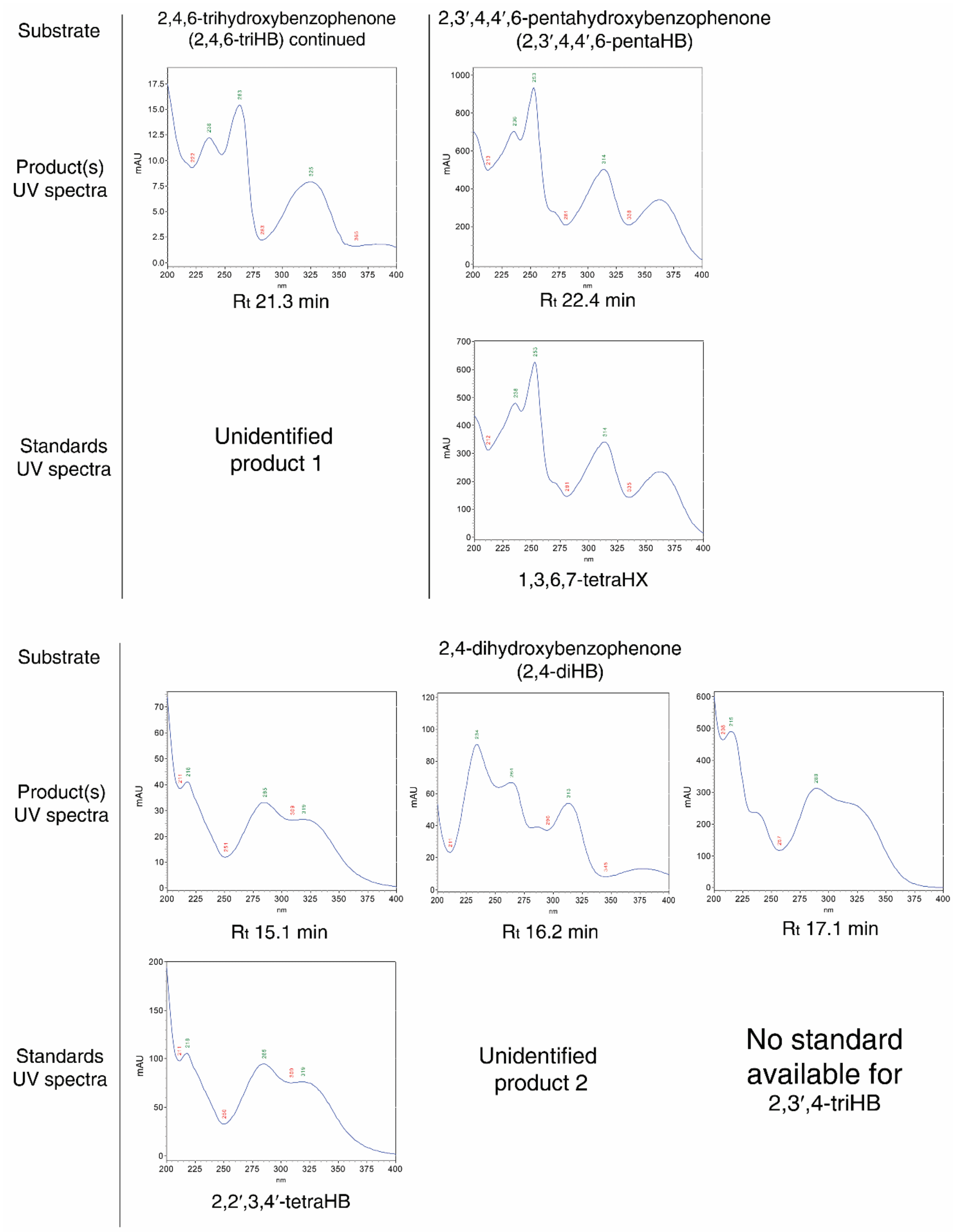

Supplementary Figure 6 | UV spectra of CYP81AA2 products and authentic compounds (II).

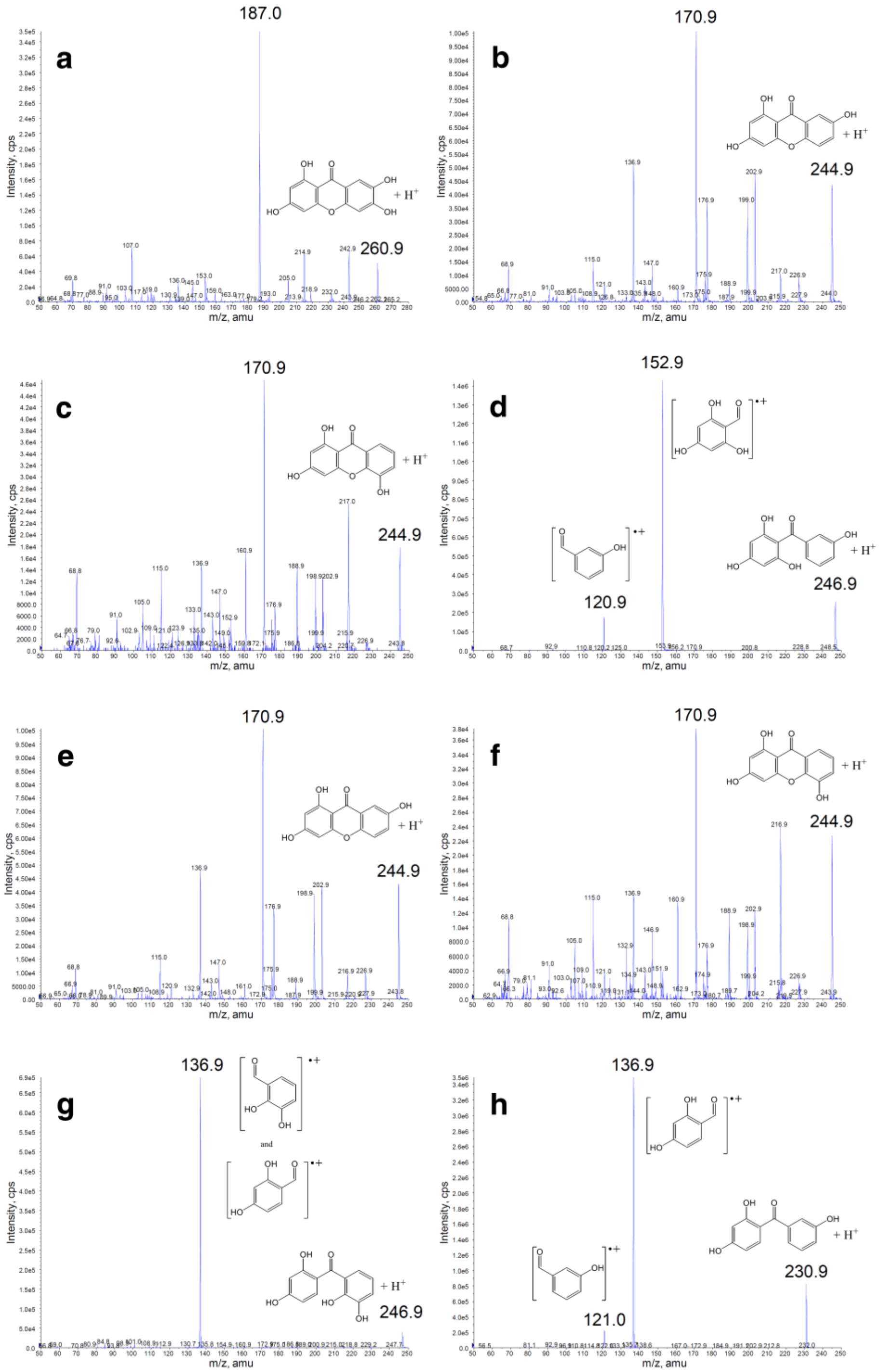

Supplementary Figure 7 | Enhanced product ion (EPI) mass spectra of CYP81AA2 products formed from various substrates. (**a**) 1,3,6,7-tetraHX (R_t = 22.3 min) from 2,3′,4,4′,6-pentaHB. (**b**) 1,3,7-triHX (R_t = 19.6 min) from 2,3′,4,6-tetraHB. (**c**) 1,3,5-triHX (R_t = 20.9 min) from 2,3′,4,6-tetraHB. (**d**) 2,3′,4,6-tetraHB (R_t = 15.1 min) from 2,4,6-triHB. (**e**) 1,3,7-triHX (R_t = 19.6 min) from 2,4,6-triHB. (**f**) 1,3,5-triHX (R_t = 20.9 min) from 2,4,6-triHB. (**g**) 2,2′,3,4′-tetraHB (R_t = 15.1 min) from 2,4-diHB. (**h**) 2,3′,4-triHB (R_t = 17.1 min) from 2,4-diHB.

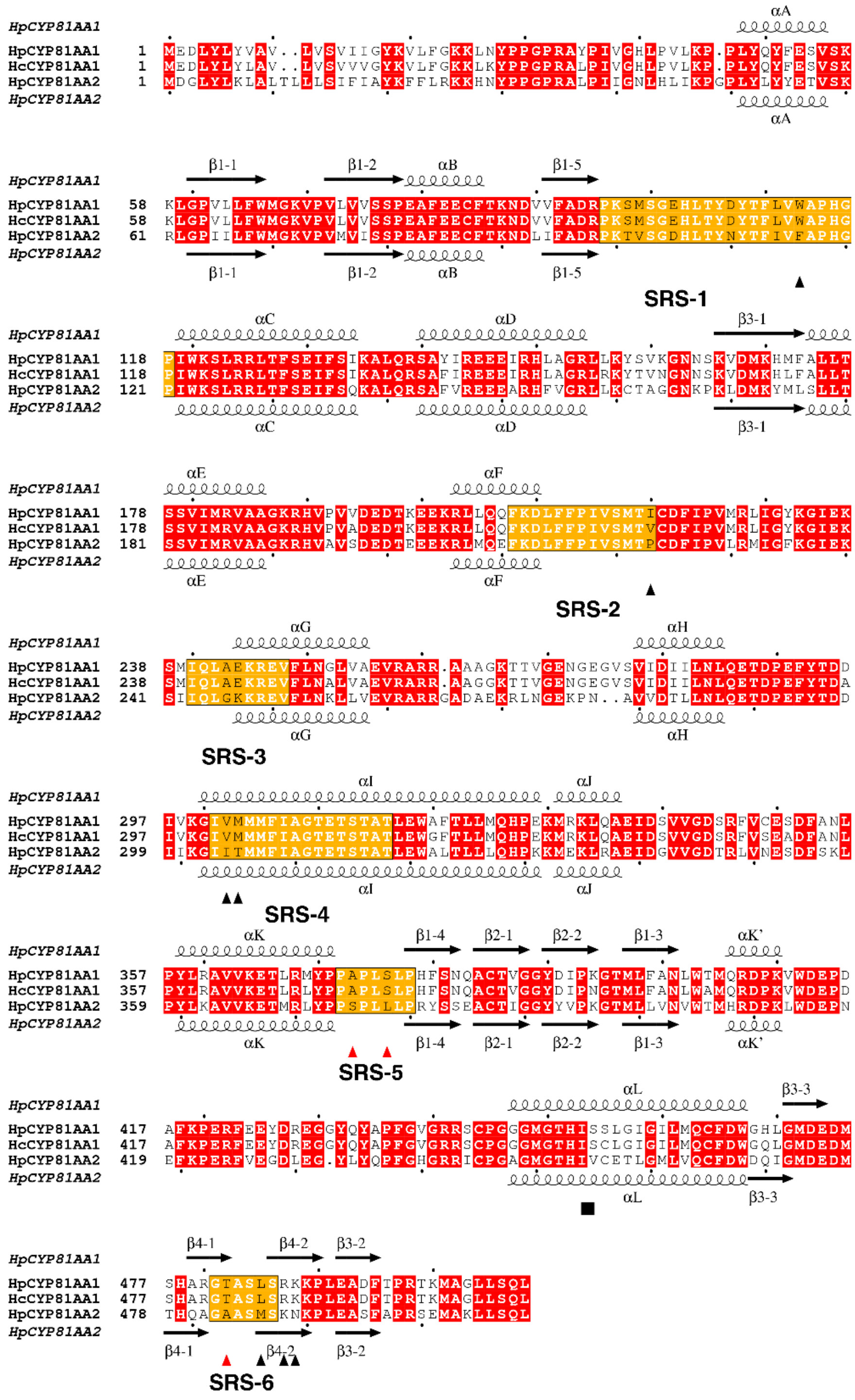

Supplementary Figure 8 | Multiple sequence alignment of HpCYP81AA1, HcCYP81AA1 and HpCYP81AA2. The conserved secondary structures of HpCYP81AA1 and HpCYP81AA2 are indicated on the top and the bottom, respectively. SRSs 1-6 are highlighted in orange. The black triangles point to positions that were mutated and the red triangles refer to the deduced regioselectivity-determining residues in HpCYP81AA2. The black square refers to the position of the C-terminal exchange.

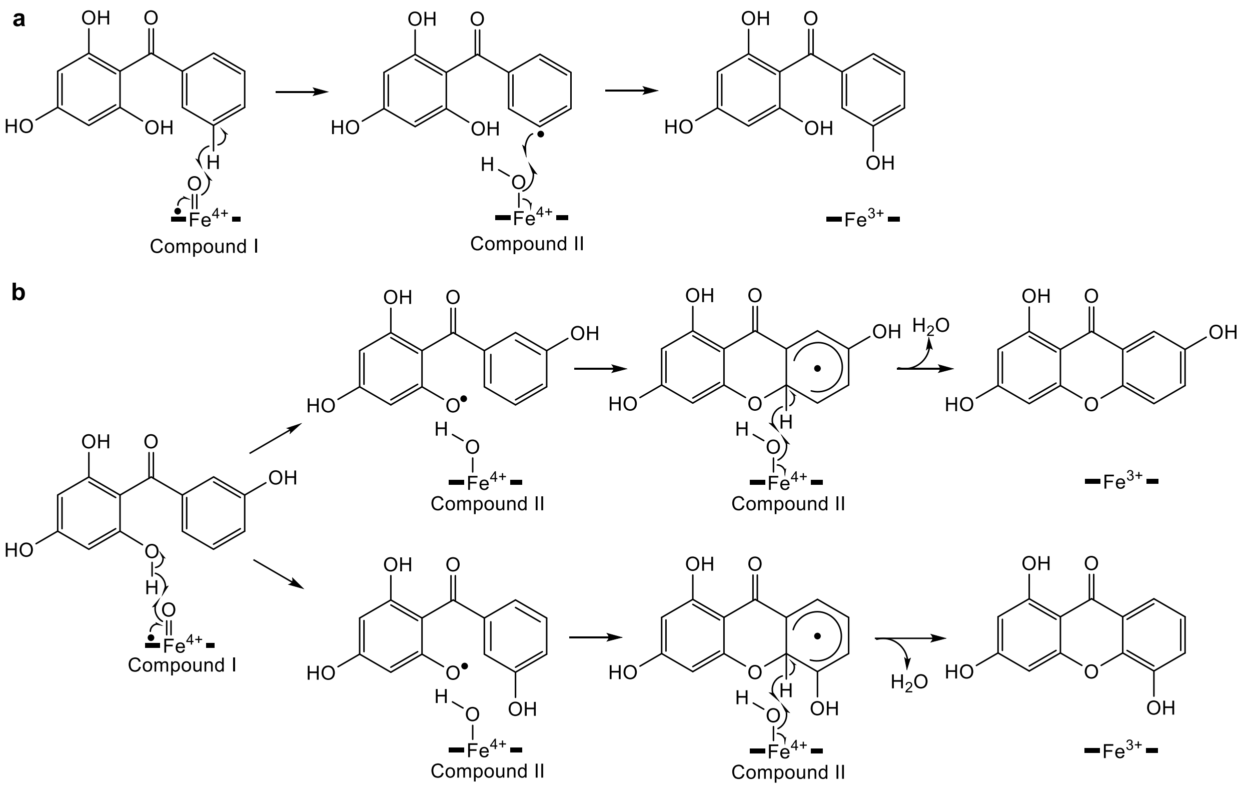

Supplementary Figure 9 | Proposed reaction processes catalyzed by CYP81AA1 and CYP81AA2. (**a**) Hydroxylation reaction at the 3′-position of 2,4,6-triHB catalyzed by both enzymes. (**b**) Regioselective C–O phenol coupling reactions occurring either *para* or *ortho* to the introduced 3′-hydroxy group catalyzed by CYP81AA1 and CYP81AA2, respectively.

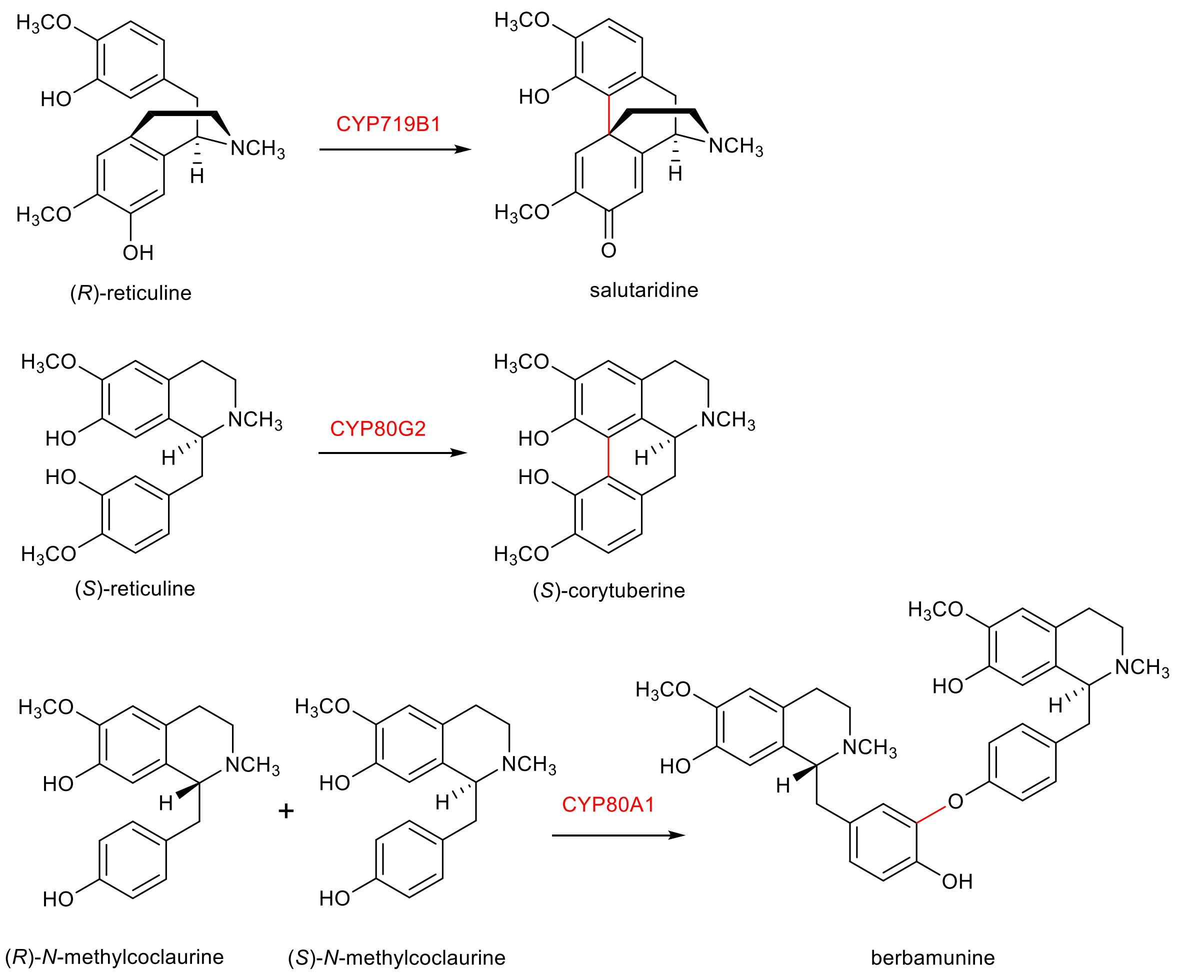

Supplementary Figure 10 | Phenol coupling reactions catalyzed by plant CYPs in isoquinoline alkaloids biosyntheses.

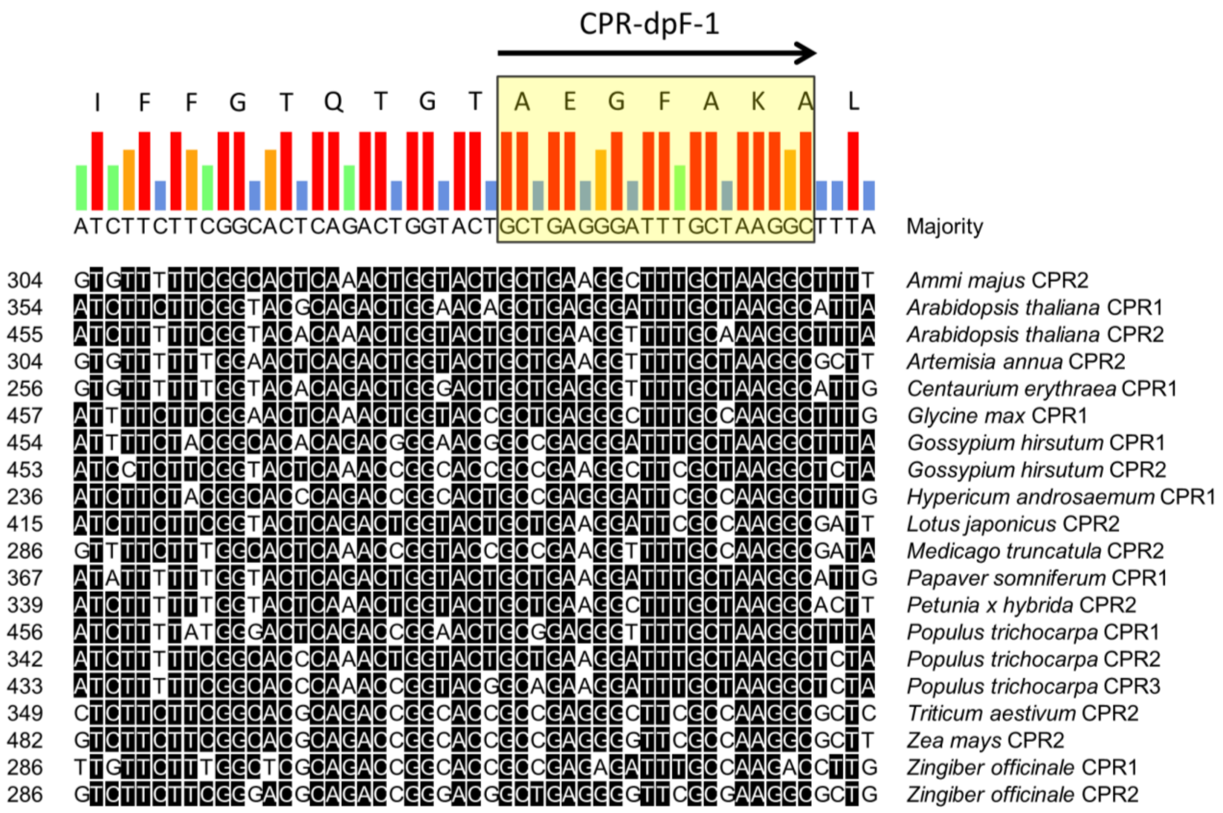

Supplementary Figure 11 | Multiple sequence alignment of the FMN binding domains of 20 plant CPRs for designing the CPR-dpF-1 primer.

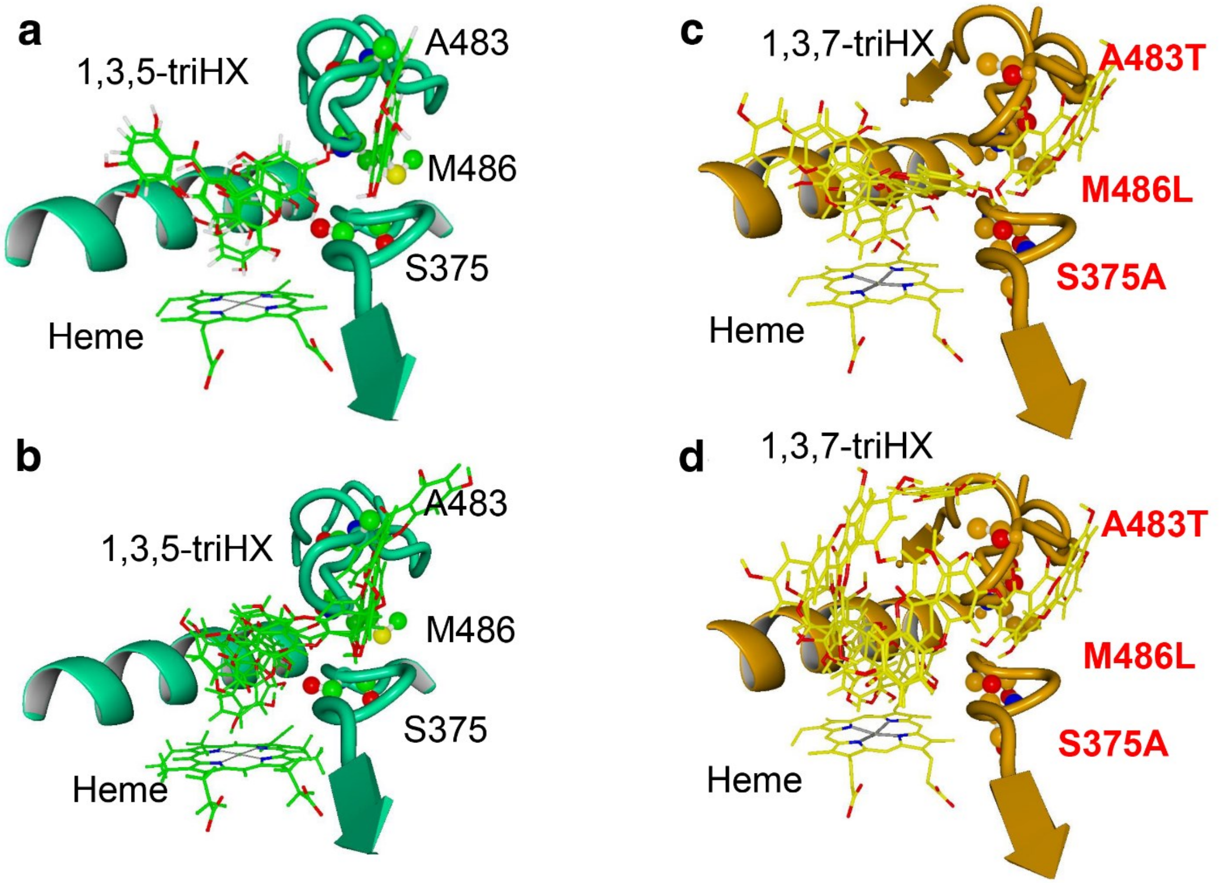

Supplementary Figure 12 | Automated product docking using AUTODOCK VINA. The active sites of wild-type CYP81AA2 (lime) with bound 1,3,5-triHX (green sticks) and the sextuple mutant (mut6) of CYP81AA2 (orange) with bound 1,3,7-triHX (yellow sticks) are illustrated. (**a**) CYP81AA2 1,3,5-triHX docking with rigid receptor, overlay of 7 clusters representing 25 docking results. (**b**) CYP81AA2 1,3,5-triHX docking with flexible receptor sidechains, overlay of 9 clusters representing 100 docking results. (**c**) CYP81AA2(mut6) 1,3,7-triHX docking with rigid receptor, overlay of 8 clusters representing 25 docking results. (**d**) CYP81AA2(mut6) 1,3,7-triHX docking with flexible receptor sidechains, overlay of 12 clusters representing 100 docking results.

Supplementary Table 1 | CYP contigs identified in a *H. calycinum* SSH library.

Contig number	Number of copies	Closest NCBI match	Closest MPGR[a] match (% identity)
21	22	CYP81C	hpa_locus_416 (95.0)
41	7	CYP81C	hpa_locus_416 (91.6)
50	11	CYP81C	hpa_locus_416 (97.2)
56	2	CYP71A	hpa_locus_18081 (98.2)
59	33	CYP81C	hpa_locus_416 (97.3)
62	36	CYP72A	hpa_locus_60 (78.2)
86	3	CYP81D	hpa_locus_12223 (70.8)
103	2	CYP81C	hpa_locus_416 (97.4)
110	3	CYP81C	hpa_locus_416 (97.2)
158	2	CYP706	hpa_locus_19031 (81.9)
181	3	CYP706	hpa_locus_19031 (82.2)
199	3	CYP81D	hpa_locus_13843 (89.7)
219	5	CYP81C	hpa_locus_416 (92.0)

[a] Medicinal Plant Genomics Resource (http://medicinalplantgenomics.msu.edu)

Supplementary Table 2 | Product profiles of CYP81AA1 and CYP81AA2[a].

Substrate	Product(s)		
	CYP81AA1	CYP81AA2	
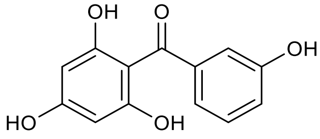<br>2,3′,4,6-tetraHB	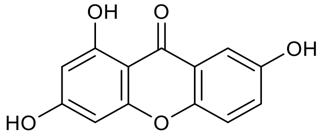<br>1,3,7-triHX	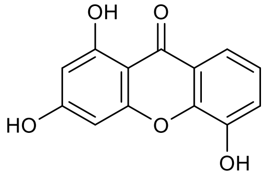<br>1,3,5-triHX	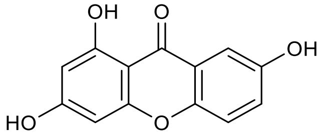<br>1,3,7-triHX (minor)<br>+ unidentified product 1 (minor)
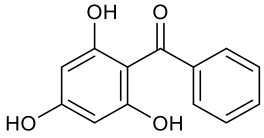<br>2,4,6-triHB	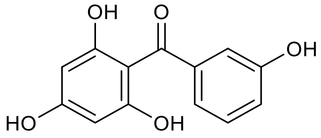<br>2,3′,4,6-tetraHB	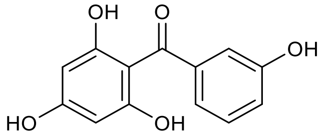<br>2,3′,4,6-tetraHB	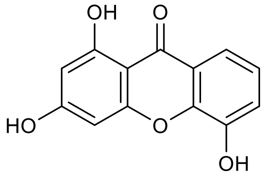<br>1,3,5-triHX<br>+ unidentified product 1 (minor)
	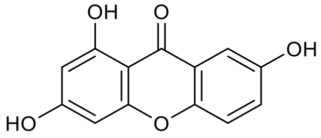<br>1,3,7-triHX	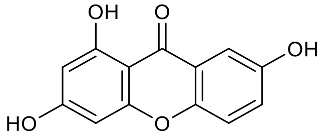<br>1,3,7-triHX (minor)	
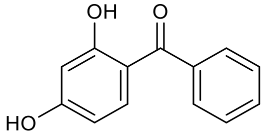<br>2,4-diHB	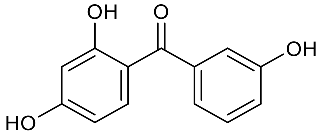<br>2,3′,4-triHB	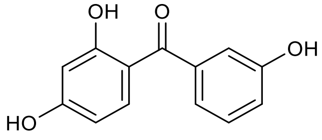<br>2,3′,4-triHB<br>+ unidentified product 2	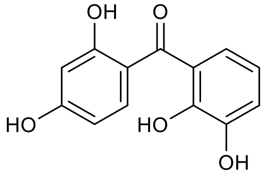<br>2,2′,3,4′-tetraHB
	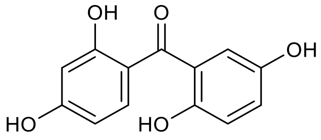<br>2,2′,4,5′-tetraHB		
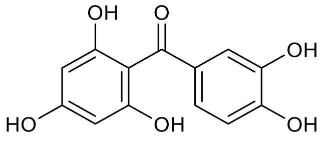<br>2,3′,4,4′,6-pentaHB	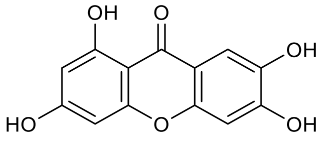<br>1,3,6,7-tetraHX	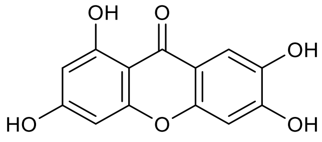<br>1,3,6,7-tetraHX	

[a] Non-accepted substrates are depicted in Supplementary Fig. 1

Supplementary Table 3 | Product profile of CYP81AA3.

Substrate	Product	Substrate	Product
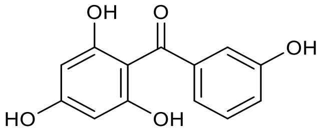<br>2,3′,4,6-tetraHB	n.d.	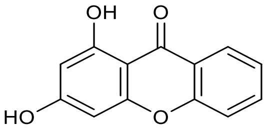<br>1,3-diHX	n.d.
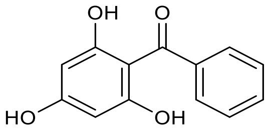<br>2,4,6-triHB	n.d.	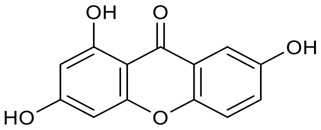<br>1,3,7-triHX	n.d.
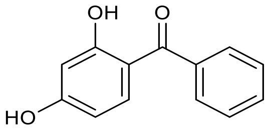<br>2,4-diHB	n.d.	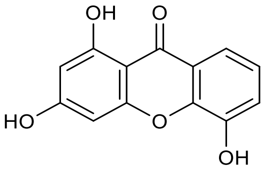<br>1,3,5-triHX	n.d.
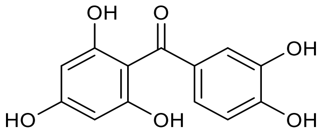<br>2,3′,4,4′,6-pentaHB	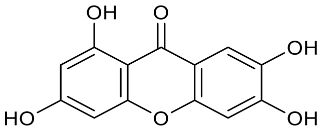<br>1,3,6,7-tetraHX	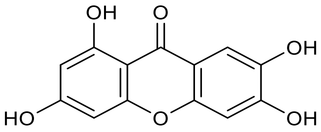<br>1,3,6,7-tetraHX	n.d.
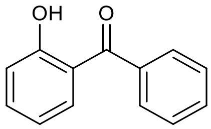<br>2-HB	n.d.	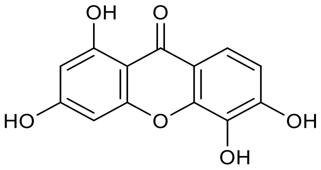<br>1,3,5,6-tetraHX	n.d.
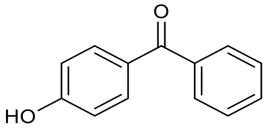<br>4-HB	n.d.		

n.d. = not detected

Supplementary Table 4 | Contact residues located within a 4 Å radius of the bound inhibitor and their standard positions and SRS numbers. Divergent residues are highlighted in red.

CYP81AA1		CYP81AA2		**Standard numbering position**	**SRS number**
Residue	Position	Residue	Position		
PHE	110	PHE	113	85	1
PHE	212	PHE	215	181	2
ILE	215	ILE	218	184	2
VAL	216	VAL	219	185	2
ILE	307	ILE	309	263	4
ALA	308	ALA	310	264	4
GLU	311	GLU	313	267	4
THR	312	THR	314	268	4
ALA	315	ALA	317	271	4
TYR	370	TYR	372	325	5
PRO	372	PRO	374	327	5
ALA	373	SER	375	328	5
LEU	377	LEU	379	331	5
THR	482	MET	486	437/439	6
ALA	483	SER	487	437.3/440	6
SER	484	LYS	488	438/441	6
LEU	485	ASN	489	439/441.1	6

Supplementary Table 5 | Product profiles of CYP81AA1 mutants.

**Enzyme mutant**	**1,3,7-triHX**	**1,3,5-triHX**
	**(% of total product)**	
Wild-type CYP81AA1	100	0
CYP81AA1-AA2	100	0
CYP81AA1 A373S	94.9 ± 1.1	5.1 ± 1.1
CYP81AA1-AA2 A373S	93.2 ± 0.2	6.8 ± 0.2
CYP81AA1 A373S/S376L	95.0 ± 0.4	5.0 ± 0.4
CYP81AA1-AA2 A373S/S376L	96.0 ± 0.8	4.0 ± 0.8
CYP81AA1 A373S/S376L/T482A	95.5 ± 0.3	4.5 ± 0.3
CYP81AA1 A373S/S376L/T482A/L485M/R487K/K488N	93.5 ± 0.6	6.5 ± 0.6
CYP81AA1 A373S/S376L/T482V	94.6 ± 0.5	5.4 ± 0.5
CYP81AA1 A373S/S376L/T482F	95.9 ± 0.3	4.1 ± 0.3
CYP81AA1 W113F/A373S/S376L/T482A	95.2 ± 0.1	4.8 ± 0.1
CYP81AA1 I220P/A373S/S376L/T482A	99.4 ± 0.1	0.6 ± 0.1
CYP81AA1 A373S/S376L/T482A/L485M/R487K/K488N/V302I/M303T	No activity detected	
CYP81AA1 W113F/I220P/A373S/S376L/T482A/L485M/R487K/K488N/ V302I/M303T	No activity detected	

Supplementary Table 6 | Accession numbers of the plant CPR sequences used to design CPR-dpF-1.

**Name**	**Accession No.**	**Name**	**Accession No.**
Ammi majus CPR2	AY532374.1	*Medicago truncatula* CPR2	XM_003610061.1
Arabidopsis thaliana CPR1	NM_118585.3	*Papaver somniferum* CPR1	U67185.1
Arabidopsis thaliana CPR2	NM_179141.2	*Petunia* x *hybrida* CPR2	DQ099545.1
Artemisia annua CPR2	DQ984181.1	*Populus trichocarpa* CPR1	XM_002307300.2
Centaurium erythraea CPR1	AY596976.1	*Populus trichocarpa* CPR2	AF302497.1
Glycine max CPR1	NM_001249813.1	*Populus trichocarpa* CPR3	AF302498.1
Gossypium hirsutum CPR1	FJ719368.1	*Triticum aestivum* CPR2	AJ303373.1
Gossypium hirsutum CPR2	FJ719369.1	*Zea mays* CPR2	EU956822.1
Hypericum androsaemum CPR1	AY520902.1	*Zingiber officinale* CPR1	AB566408.1
Lotus japonicus CPR2	AB433810.1	*Zingiber officinale* CPR2	AB566409.1

Supplementary Table 7 | List of primers used for core fragment amplification, RACE, qRT-PCR and cloning.

Primer name	Sequence 5′→3′
Core fragments amplification	
Contig59-F1	AAGGTCCCCGTCCTCGTTGTC
Contig21-R1	CGTATTCCTCGAACCTCTC
CPR-dpF-1	GCNGARGGNTTYGCHAAGGC
CPR-R1	CTTGGACAATGGTGTGGAGAGTTC
3′ and 5′ RACE	
CYP81-3RACE1	TCCTAATGCAACACCCAGAG
CYP81-3RACE2	CTACCTAAGGGCCGTCGTGA
CYP81-5RACE1	CCGCTTCTCCTCCTTGGTGTCC
CYP81-5RACE2	CGTCGTTCTTGGTGAAGCACTCCTC
CPR-3RACE1	GCTCCATTCAGGGGTTTCC
CPR-3RACE2	GGTTAGCCCTGAAAGAATCC
CPR-5RACE1	ATCCATTTCGGGCCACAATGCCTCTC
CPR-5RACE2	TGCCTGTTCCCTAAGCCAAACACTGC
51544-3RACE1	GACGCCATGCGACTTCATCC
51544-3RACE2	TCCTTAACGGCTTACTTGCCAATA
qRT-PCR	
Hc81AA1-qF	TCTCGATGACCGTATGTGACTT
Hc81AA1-qR	CGTTAAGGAAGACCTCCCTCT
HcCPR2-qF	TGACTATGCTGCGGATGATGAA
HcCPR2-qR	GCCAAGAAGAGGACAACCAAATC
HcBPS-qF	AAGGAAAGAAGAGGGCTAGTGT
HcBPS-qR	ATGTGCTCGCTGTTAGTGTTC
Actin-qF	CGGCAGTGGTTGTGAACAT
Actin-qR	TCTCGCTGGTCGTGATCTG
Histone-H2A-qF	AACATCTACTCTTTGGACGACTTG
Histone-H2A-qR	AATTGCTGGAGGTGGAGTTATTC
Cloning*	
HcCPR2-*Bam*HI-F	GTAATGGATCCGATGGAACCGACGGGGAGC
HcCPR2-*Hin*dIII-R	GTCTGAAGCTTTCACCATACGTCGCGAAGGTAC
Hc81AA1-*Eco*RI-F	ATTGAATTCGGATGGAGGACTTGTACTTGTACC
Hc81AA1-*Pac*I-R	ACGTTAATTAATTAGAGCTGGGAAAGGAGACC
Hp81AA1-*Spe*I-F	TATGACTAGTAATGGAGGACTTGTATCTGTACG
Hp81AA1-*Pac*I-R	ACGTTAATTAATTAGAGCTGGGAGAGGAGG
Hp81AA2-*Eco*RI-F	ATTGAATTCATGGACGGTTTATACTTAAAACTAGCC
Hp81AA2-*Pac*I-R	ACGTTAATTAACTAGAGTTGGGAAAGGAGCTTG
Hp81AA2-*Spe*I-F	ATTACTAGTAATGGACGGTTTATACTTAAAACTAGC
Hp81AA3-*Spe*I-F	TATGACTAGTAATGGAATTGTATTTGTATCTAGCCG
Hp81AA3-*Pac*I-R	ACGTTAATTAACTAAGAGAGTAGAGAACTGAGG

* Introduced restriction site sequences are underlined

Supplementary Table 8 | Primers used for C-terminal exchanges and site-directed mutagenesis.

Primer name	Sequence 5′→3′
Overlapping primers used for C-terminal exchanges	
AA1-AA2-ex	CGGAGGGATGGGGACCCACATTGTCTGCGAGACATTGGGAATG
AA2-AA1-ex	CCGGAATGGGAACCCATATCTCGTCCCTGGGGATTGGAATTCTTA
Primers used for site-directed mutagenesis (mutated positions are highlighted in red)	
AA1-A373S-F	CCGCCGTCCCCACTCTCCCTCCCCCACTTCTCCAACCAAG
AA1-A373S-R	GTGGGGACGGCGGGTACATCCTGAGCGTCTCCTTCACGAC
AA1-ASSL-F	CGCCGTCCCCACTCTTGCTCCCCCACTTCTCCAACCAAG
AA1-T482V-F	AGCCACGCGCGTGGTGTCGCGAGTCTGTCAAGGAAGAAG
AA1-T482F-F	AGCCACGCGCGTGGTTTCGCGAGTCTGTCAAGGAAGAAG
AA1-T482uni-R	ACGCGCGTGGCTCATGTCCTCGTCCATGCCGAGGTGGC
AA1-T482A-F	CGTGGTGCCGCGAGTCTGTCAAGGAAGAAGCCATTGGAG
AA1-T482A-R	TCGCGGCACCACGCGCGTGGCTCATGTCCTCGTCCATGC
AA1-TALMRKKN-F	CGTGGTGCCGCGAGTATGTCAAAGAACAAGCCATTGGAG
AA1-VIMT-F	GAATTATTACGATGATGTTTATTGCCGGGACCGAGACG
AA1-VIMT-R	TCATCGTAATAATTCCCTTGACGATGTCATCCGTG
AA1-W113F-F	TCGTGTTCGCCCCGCACGGCCCCATCTGGAAGAGCCTC
AA1-W113F-R	GGGGCGAACACGAGGAAGGTGTAGTCGTAGGTGAGGTGC
AA1-I220P-F	ATGACCCCATGCGACTTCATCCCGGTGATGAGGCTGATC
AA1-I220P-R	TCGCATGGGGTCATGGAGACGATAGGAAAGAAGAGATCC
AA2-A483T-F	GCCGGTACGGCTAGTATGTCCAAGAACAAGCCATTGG
AA2-A483T-R	TAGCCGTACCGGCTTGGTGGGTCATGTCCTCGTCCATAC
AA2-ATMLKRNK-F	GCCGGTACGGCTAGTCTGTCCAGGAAGAAGCCATTGG
AA2-L378S-F	CGCTCTCGCTACCTCGTTATTCGAGCGAGGCTTGCAC
AA2-L378S-R	GGTAGCGAGAGCGGGGACGGAGGATACAGCCTCATCGTC
AA2-S375A-F	CCTCCGGCCCCGCTCTTGCTACCTCGTTATTCGAGCGAG
AA2-S375A-R	GCGGGGCCGGAGGATACAGCCTCATCGTCTCCTTCACG
AA2-SALS-F	CCTCCGGCCCCGCTCTCGCTACCTCGTTATTCGAGCGAG

Supplementary Table 9 | Templates and primers used to generate mutations.

Template	Primers	Mutant
**Mutants of CYP81AA1**		
Wild-type CYP81AA1	AA1-A373S-F<br>AA1-A373S-R	A373S
AA1-AA2	AA1-A373S-F<br>AA1-A373S-R	AA1-AA2 A373S
A373S	AA1-AS-SL-F<br>AA1-A373S-R	A373S/S376L
AA1-AA2 A373S	AA1-AS-SL-F<br>AA1-A373S-R	AA1-AA2 A373S/S376L
A373S/S376L	AA1-T482A-F<br>AA1-T482A-R	A373S/S376L/T482A
A373S/S376L	AA1-TALMRKKN-F<br>AA1-T482A-R	A373S/S376L/T482A/L485M/R487K/K488N
A373S/S376L	AA1-T482V-F<br>AA1-T482uni-R	A373S/S376L/T482V
A373S/S376L	AA1-T482F-F<br>AA1-T482uni-R	A373S/S376L/T482F
A373S/S376L/T482A	AA1-W113F-F<br>AA1-W113F-R	W113F/A373S/S376L/T482A
CYP81AA1 A373S/S376L/T482A	AA1-I220P-F<br>AA1-I220P-R	I220P/A373S/S376L/T482A
A373S/S376L/T482A/L485M/R487K/K488N	AA1-VIMT-F<br>AA1-VIMT-R	A373S/S376L/T482A/L485M/R487K/K488N/V302I/M303T
A373S/S376L/T482A/L485M/R487K/K488N/V302I/M303T	AA1-W113F-F<br>AA1-W113F-R	W113F/A373S/S376L/T482A/L485M/R487K/K488N/V302I/M303T
W113F/A373S/S376L/T482A/L485M/R487K/K488N/V302I/M303T	AA1-I220P-F<br>AA1-I220P-R	W113F/I220P/A373S/S376L/T482A/L485M/R487K/K488N/V302I/M303T
**Mutants of CYP81AA2**		
Wild-type CYP81AA2	AA2-S375A-F<br>AA2-S375A-R	S375A
Wild-type CYP81AA2	AA2-L378S-F<br>AA2-L378S-R	L378S
Wild-type CYP81AA2	AA2-A483T-F<br>AA2-A483T-R	A483T
S375A	AA2-SALS-F<br>AA2-S375A-F	S375A/L378S
S375A	AA2-A483T-F<br>AA2-A483T-R	S375A/A483T
L378S	AA2-A483T-F<br>AA2-A483T-R	L378S/A483T
S375A/L378S	AA2-A483T-F<br>AA2-A483T-R	S375A/L378S/A483T
S375A/L378S	AA2-ATMLKRNK-F<br>AA2-A483T-R	S375A/L378S/A483T/M486L/K488R/N489K

Supplementary Methods

Synthesis of standards. 1,3,7-Trihydroxyxanthone, 1,3,5-trihydroxyxanthone, and 1,3,5,6-tetrahydroxyxanthone were synthesized by the Grover, Shah and Shah method[1,2]. 2,2′,3,4′-Tetrahydroxybenzophenone and 2,2′,4,5′-tetrahydroxybenzophenone were prepared in the laboratory of Prof. Dr. Qidong You, Pharmaceutical University, Nanjing, China[3]. 2,3′,4,6-Tetrahydroxybenzophenone and 1,3-dihydroxyxanthone were from our laboratory collection[4]. 1,3,6,7-Tetrahydroxyxanthone was isolated from *H. perforatum in vitro* roots as previously reported[5]. All other chemicals were commercially available.

Supplementary References

1. Genoux-Bastide, E. et al. Identification of xanthones as selective killers of cancer cells overexpressing the ABC transporter MRP1. *ChemMedChem* **6**, 1478-84 (2011).
2. Grover, P.K., Shah, G.D. & Shah, R.C. Xanthones. Part IV. A new synthesis of hydroxyxanthones and hydroxybenzophenones. *J. Chem. Soc.*, 3982-3985 (1955).
3. Zhang, X.-J. et al. Microwave-assisted efficient and green synthesis of hydroxyxanthone in water. *Synth. Commun.* **42**, 2952-2958 (2012).
4. Schmidt, W., Peters, S. & Beerhues, L. Xanthone 6-hydroxylase from cell cultures of *Centaurium erythraea* RAFN and *Hypericum androsaemium* L. *Phytochemistry* **53**, 427-431 (2000).
5. Tocci, N. et al. A three-step culture system to increase the xanthone production and antifungal activity of *Hypericum perforatum* subsp. angustifolium *in vitro* roots. *Plant Physiol. Biochem.* **57**, 54-8 (2012).
