# Supplementary material for: Bifunctional CYP81AA proteins catalyse identical hydroxylations but alternative regioselective phenol couplings in plant xanthone biosynthesis
Source: Nat Commun. 2016 May 5;7:11472. doi: 10.1038/ncomms11472 (PMC4858744; doi:10.1038/ncomms11472)
Supplement: Supplementary Data 1 — Product docking [file ncomms11472-s2.pdf]

a)CYP81AA2 rigid receptor, all 25 docking results

```
Global docking result analysis
==============================
25 VINA docking runs of the ligand object 2 to the receptor object 1 yielded the following results,
sorted by binding energy [more positive energies indicate stronger binding, and negative energies mean no binding]

Run |Bind.energy[kcal/mol]|Dissoc. constant [pM]| Contacting receptor residues
----+---------------------+----------------------+---------------------------
001 |         000007.9230 | 00000001557270.2500 | PHE   116 ILE   218 VAL   219 MET   306 ILE   309 ALA   310 THR   314 SER
375 LEU   378 LEU   379 CYS   445 ALA   484 SER   485 HEM A 600
002 |         000007.9230 | 00000001557270.2500 | PHE   116 ILE   218 VAL   219 MET   306 ILE   309 ALA   310 THR   314 SER
375 LEU   378 LEU   379 CYS   445 ALA   484 SER   485 HEM A 600
003 |         000007.7130 | 00000002219709.0000 | VAL   115 PHE   116 ILE   218 VAL   219 MET   306 ALA   310 THR   314 SER
375 LEU   378 LEU   379 CYS   445 ALA   484 SER   485 HEM A 600
004 |         000007.7130 | 00000002219709.0000 | VAL   115 PHE   116 ILE   218 VAL   219 MET   306 ALA   310 THR   314 SER
375 LEU   378 LEU   379 CYS   445 ALA   484 SER   485 HEM A 600
005 |         000007.5570 | 00000002888321.7500 | PHE   116 PHE   215 ILE   218 MET   306 ILE   309 ALA   310 THR   314 PRO
374 SER   375 LEU   379 CYS   445 ALA   484 SER   485 MET   486 HEM A 600
006 |         000007.5570 | 00000002888321.7500 | PHE   116 PHE   215 ILE   218 MET   306 ILE   309 ALA   310 THR   314 PRO
374 SER   375 LEU   379 CYS   445 ALA   484 SER   485 MET   486 HEM A 600
007 |         000007.5200 | 00000003074446.2500 | VAL   115 PHE   116 PHE   215 ILE   218 MET   306 ILE   309 GLU   313 THR
314 ALA   317 PRO   374 SER   375 LEU   379 SER   485 MET   486
008 |         000007.5200 | 00000003074446.2500 | VAL   115 PHE   116 PHE   215 ILE   218 MET   306 ILE   309 GLU   313 THR
314 ALA   317 PRO   374 SER   375 LEU   379 SER   485 MET   486
009 |         000007.3530 | 00000004075487.2500 | VAL   115 PHE   116 PHE   215 ILE   218 MET   306 ALA   310 THR   314 SER
375 LEU   379 CYS   445 ALA   484 SER   485 HEM A 600
010 |         000007.3530 | 00000004075487.2500 | VAL   115 PHE   116 PHE   215 ILE   218 MET   306 ALA   310 THR   314 SER
375 LEU   379 CYS   445 ALA   484 SER   485 HEM A 600
011 |         000007.3310 | 00000004229663.0000 | LEU   107 PHE   113 VAL   115 PHE   116 LEU   214 PHE   215 ILE   218 ARG
249 THR   305 MET   306 ILE   309 THR   314 SER   375 LEU   379 SER   485
012 |         000007.1980 | 00000005294147.5000 | VAL   115 PHE   116 ILE   218 MET   306 ILE   309 ALA   310 THR   314 SER
375 LEU   379 CYS   445 HEM A 600
013 |         000007.1790 | 00000005466674.5000 | LEU   107 PHE   113 VAL   115 PHE   116 LEU   214 PHE   215 ILE   218 ARG
249 MET   306 ILE   309 GLU   313 THR   314 SER   375 LEU   379 SER   485
014 |         000007.0950 | 00000006299354.5000 | VAL   115 PHE   116 PHE   215 ILE   218 MET   306 ILE   309 GLU   313 THR
314 ALA   317 PRO   374 SER   375 LEU   379 SER   485 MET   486
015 |         000007.0760 | 00000006504639.5000 | LEU   51 TYR   52 SER   375 PRO   376 LEU   377 LEU   378 LEU   379 PRO
380 MET   399 LEU   401 GLY   482 ALA   483 ALA   484 MET   486
016 |         000007.0360 | 00000006958948.5000 | LEU   107 THR   108 PHE   113 VAL   115 PHE   116 LEU   214 PHE   215 ILE
218 ARG   249 THR   305 MET   306 ILE   309 ALA   310 GLU   313 THR   314 LEU   379
017 |         000007.0260 | 00000007077399.5000 | VAL   115 PHE   116 PHE   215 ILE   218 MET   306 ALA   310 THR   314 PRO
374 SER   375 LEU   379 CYS   445 ALA   484 SER   485 HEM A 600
018 |         000006.8570 | 00000009413527.0000 | PHE   116 PHE   215 ILE   218 ILE   309 GLU   313 THR   314 SER   375 LEU
378 LEU   379 PRO   380 SER   485
```

```
019 |        000006.8020 | 00000010329229.0000 | VAL   115 PHE   116 ILE   218 VAL   219 MET   306 ILE   309 ALA   310 THR
314 SER   375 LEU   378 LEU   379 PRO   380 SER   485
020 |        000006.6950 | 00000012373710.0000 | VAL   115 PHE   116 PHE   215 ILE   218 MET   306 ILE   309 ALA   310 GLU
313 THR   314 LEU   379 CYS   445 HEM A 600
021 |        000006.6820 | 00000012648210.0000 | ILE   46 LEU   51 VAL   219 SER   375 PRO   376 LEU   377 LEU   378 PRO
380 ALA   483 ALA   484 SER   485 MET   486
022 |        000006.6490 | 00000013372678.0000 | VAL   115 PHE   116 PHE   215 ILE   218 MET   306 ILE   309 THR   314 PRO
374 SER   375 LEU   379 SER   485 HEM A 600
023 |        000006.6480 | 00000013395267.0000 | PHE   116 PHE   215 ILE   218 ILE   309 ALA   310 GLU   313 THR   314 SER
375 LEU   378 LEU   379 PRO   380 SER   485
024 |        000006.5540 | 00000015698361.0000 | VAL   115 PHE   116 PHE   215 ILE   218 VAL   219 MET   306 ILE   309 ALA
310 GLU   313 THR   314 LEU   379 ALA   484 SER   485 HEM A 600
025 |        000006.4300 | 00000019352964.0000 | PHE   116 PHE   215 ILE   218 ILE   309 ALA   310 GLU   313 THR   314 PRO
374 SER   375 LEU   377 LEU   378 LEU   379 PRO   380 ALA   484 SER   485 MET   486

After clustering the 25 runs, the following 7 distinct complex conformations were found:
[They all differ by at least 5.0 A heavy atom RMSD]

Clu |Bind.energy[kcal/mol]|Dissoc. constant [pM]| Contacting receptor residues

----+---------------------+---------------------+----------------------------

001 |        000007.9230 | 00000001557270.2500 | PHE   116 ILE   218 VAL   219 MET   306 ILE   309 ALA   310 THR   314 SER
375 LEU   378 LEU   379 CYS   445 ALA   484 SER   485 HEM A 600

002 |        000007.7130 | 00000002219709.0000 | VAL   115 PHE   116 ILE   218 VAL   219 MET   306 ALA   310 THR   314 SER
375 LEU   378 LEU   379 CYS   445 ALA   484 SER   485 HEM A 600

003 |        000007.5200 | 00000003074446.2500 | VAL   115 PHE   116 PHE   215 ILE   218 MET   306 ILE   309 GLU   313 THR
314 ALA   317 PRO   374 SER   375 LEU   379 SER   485 MET   486

004 |        000007.3310 | 00000004229663.0000 | LEU   107 PHE   113 VAL   115 PHE   116 LEU   214 PHE   215 ILE   218 ARG
249 THR   305 MET   306 ILE   309 THR   314 SER   375 LEU   379 SER   485

005 |        000007.0760 | 00000006504639.5000 | LEU   51 TYR   52 SER   375 PRO   376 LEU   377 LEU   378 LEU   379 PRO
380 MET   399 LEU   401 GLY   482 ALA   483 ALA   484 MET   486

006 |        000007.0360 | 00000006958948.5000 | LEU   107 THR   108 PHE   113 VAL   115 PHE   116 LEU   214 PHE   215 ILE
218 ARG   249 THR   305 MET   306 ILE   309 ALA   310 GLU   313 THR   314 LEU   379

007 |        000006.6820 | 00000012648210.0000 | ILE   46 LEU   51 VAL   219 SER   375 PRO   376 LEU   377 LEU   378 PRO
380 ALA   483 ALA   484 SER   485 MET   486
```

b) CYP81AA2 flexible receptor sidechains, all 100 docking results

```
Ensemble docking result analysis
================================

The ligand was docked 20 times ["Run"] with VINA against each of the 5 receptors ["Rec"] in the ensemble, yielding the
following 100 results, sorted by binding energy:
[More positive energies indicate stronger binding, and negative energies mean no binding]

Rec | Run |Bind.energy[kcal/mol]|Dissoc. constant [pM]| Contacting receptor residues
----+-----+---------------------+---------------------+----------------------------
002 | 001 |         000008.2560 | 00000000887711.3125 | VAL   115 PHE   116 PHE   215 ILE   218 MET   306 ILE   309 ALA   310
THR   314 PRO   374 SER   375 LEU   379 CYS   445 ALA   484 SER   485 MET   486 HEM A 600
005 | 001 |         000008.2050 | 00000000967509.5625 | VAL   115 PHE   116 PHE   215 ILE   218 ILE   309 ALA   310 THR   314
PRO   374 SER   375 LEU   379 CYS   445 ALA   484 SER   485 MET   486 HEM A 600
003 | 001 |         000008.0680 | 00000001219207.3750 | VAL   115 PHE   116 PHE   215 ILE   218 MET   306 ILE   309 ALA   310
THR   314 PRO   374 SER   375 LEU   379 CYS   445 ALA   484 SER   485 MET   486 HEM A 600
004 | 001 |         000007.9640 | 00000001453150.5000 | PHE   215 ILE   218 ILE   309 ALA   310 THR   314 PRO   374 SER   375
CYS   445 ALA   484 SER   485 MET   486 HEM A 600
005 | 002 |         000007.9390 | 00000001515778.8750 | VAL   115 PHE   116 PHE   215 ILE   218 MET   306 ILE   309 GLU   313
THR   314 ALA   317 PRO   374 SER   375 LEU   379 SER   485
001 | 001 |         000007.9190 | 00000001567819.3750 | VAL   115 PHE   116 ILE   218 VAL   219 MET   306 ILE   309 ALA   310
THR   314 SER   375 LEU   378 LEU   379 CYS   445 ALA   484 SER   485 HEM A 600
005 | 003 |         000007.8210 | 00000001849826.5000 | VAL   115 PHE   116 PHE   215 ILE   218 VAL   219 MET   306 ILE   309
ALA   310 THR   314 SER   375 LEU   378 LEU   379 CYS   445 ALA   484 SER   485 HEM A 600
004 | 002 |         000007.8170 | 00000001862357.3750 | VAL   115 PHE   116 ILE   218 MET   306 ALA   310 THR   314 PRO   374
SER   375 LEU   379 CYS   445 HEM A 600
005 | 004 |         000007.7460 | 00000002099455.7500 | VAL   115 PHE   116 PHE   215 ILE   218 MET   306 ALA   310 THR   314
PRO   374 SER   375 LEU   379 ALA   484 SER   485 HEM A 600
001 | 002 |         000007.7200 | 00000002193638.0000 | VAL   115 PHE   116 ILE   218 VAL   219 MET   306 ALA   310 THR   314
PRO   374 SER   375 LEU   378 LEU   379 CYS   445 ALA   484 SER   485 HEM A 600
003 | 002 |         000007.7140 | 00000002215965.7500 | VAL   115 PHE   116 PHE   215 ILE   218 MET   306 ALA   310 THR   314
PRO   374 SER   375 LEU   379 CYS   445 ALA   484 SER   485 HEM A 600
002 | 002 |         000007.6980 | 00000002276623.2500 | VAL   115 PHE   116 PHE   215 ILE   218 MET   306 ALA   310 THR   314
PRO   374 SER   375 LEU   379 CYS   445 ALA   484 SER   485 HEM A 600
004 | 003 |         000007.6310 | 00000002549193.7500 | VAL   115 PHE   116 PHE   215 ILE   218 MET   306 ALA   310 THR   314
PRO   374 SER   375 LEU   379 CYS   445 ALA   484 SER   485 HEM A 600
005 | 005 |         000007.6150 | 00000002618972.7500 | VAL   115 PHE   116 ILE   218 VAL   219 MET   306 ALA   310 SER   375
LEU   378 LEU   379 CYS   445 ALA   484 SER   485 HEM A 600
001 | 003 |         000007.5550 | 00000002898088.0000 | VAL   115 PHE   116 PHE   215 ILE   218 MET   306 ALA   310 THR   314
PRO   374 SER   375 LEU   379 CYS   445 ALA   484 SER   485 MET   486 HEM A 600
004 | 004 |         000007.5550 | 00000002898088.0000 | VAL   115 PHE   116 PHE   215 ILE   218 MET   306 ALA   310 THR   314
PRO   374 SER   375 LEU   379 SER   485 HEM A 600
001 | 004 |         000007.5170 | 00000003090053.0000 | VAL   115 PHE   116 PHE   215 ILE   218 MET   306 ILE   309 GLU   313
THR   314 ALA   317 PRO   374 SER   375 LEU   379 SER   485 MET   486
```

003 | 003 | 000007.5090 | 00000003132059.5000 | VAL   115 PHE   116 ILE   218 VAL   219 MET   306 ALA   310 SER   375
LEU   378 LEU   379 CYS   445 ALA   484 SER   485 HEM A 600
002 | 003 | 000007.5070 | 00000003142650.2500 | VAL   115 PHE   116 ILE   218 VAL   219 MET   306 ALA   310 SER   375
LEU   378 LEU   379 CYS   445 ALA   484 SER   485 HEM A 600
004 | 005 | 000007.4550 | 00000003430935.5000 | VAL   115 PHE   116 PHE   215 ILE   218 MET   306 ILE   309 GLU   313
THR   314 ALA   317 PRO   374 SER   375 LEU   379 SER   485 MET   486
005 | 006 | 000007.3220 | 00000004294403.5000 | VAL   115 PHE   116 PHE   215 ILE   218 MET   306 ILE   309 GLU   313
THR   314 ALA   317 PRO   374 SER   375 LEU   379 SER   485 MET   486
003 | 004 | 000007.2830 | 00000004586593.0000 | PHE   215 ILE   218 MET   306 ILE   309 ALA   310 THR   314 SER   375
LEU   378 LEU   379 CYS   445 ALA   484 SER   485 HEM A 600
002 | 004 | 000007.2450 | 00000004890402.5000 | VAL   115 PHE   116 PHE   215 ILE   218 MET   306 ALA   310 THR   314
PRO   374 SER   375 LEU   379 CYS   445 ALA   484 SER   485 HEM A 600
003 | 005 | 000007.2120 | 00000005170516.0000 | VAL   115 PHE   116 PHE   215 ILE   218 MET   306 ALA   310 THR   314
PRO   374 SER   375 LEU   379 CYS   445 ALA   484 SER   485 HEM A 600
001 | 005 | 000007.1980 | 00000005294147.5000 | LEU   107 VAL   115 PHE   116 ILE   218 MET   306 ILE   309 ALA   310
THR   314 SER   375 LEU   379 CYS   445 HEM A 600
004 | 006 | 000007.1920 | 00000005348033.5000 | VAL   115 PHE   116 ILE   218 MET   306 ALA   310 SER   375 LEU   378
LEU   379 CYS   445 ALA   484 SER   485 HEM A 600
004 | 007 | 000007.1850 | 00000005411593.5000 | VAL   115 PHE   116 ILE   218 VAL   219 MET   306 ALA   310 THR   314
SER   375 LEU   379 ALA   484 SER   485 HEM A 600
002 | 005 | 000007.1480 | 00000005760319.0000 | VAL   115 PHE   116 PHE   215 ILE   218 VAL   219 MET   306 ALA   310
THR   314 PRO   374 SER   375 LEU   379 CYS   445 ALA   484 SER   485 HEM A 600
003 | 006 | 000007.1460 | 00000005779796.5000 | VAL   115 PHE   116 PHE   215 ILE   218 MET   306 ALA   310 THR   314
PRO   374 SER   375 LEU   379 CYS   445 ALA   484 SER   485 HEM A 600
004 | 008 | 000007.1300 | 00000005938007.0000 | VAL   115 PHE   116 PHE   215 ILE   218 MET   306 THR   314 PRO   374
SER   375 LEU   379 ALA   484 SER   485 MET   486 HEM A 600
005 | 007 | 000007.1120 | 00000006121176.0000 | VAL   115 PHE   116 PHE   215 ILE   218 MET   306 ILE   309 ALA   310
THR   314 PRO   374 SER   375 LEU   379 SER   485 HEM A 600
003 | 007 | 000007.1090 | 00000006152249.0000 | VAL   115 PHE   116 PHE   215 ILE   218 MET   306 ILE   309 GLU   313
THR   314 ALA   317 PRO   374 SER   375 LEU   379 SER   485
005 | 008 | 000007.0970 | 00000006278126.0000 | VAL   115 PHE   116 PHE   215 ILE   218 MET   306 ILE   309 GLU   313
THR   314 PRO   374 SER   375 LEU   379 SER   485 HEM A 600
004 | 009 | 000007.0630 | 00000006648939.0000 | VAL   115 PHE   116 PHE   215 ILE   218 MET   306 ALA   310 THR   314
PRO   374 SER   375 LEU   379 CYS   445 ALA   484 SER   485 HEM A 600
004 | 010 | 000007.0520 | 00000006773536.5000 | VAL   115 PHE   116 PHE   215 ILE   218 MET   306 ILE   309 GLU   313
THR   314 PRO   374 SER   375 LEU   379 SER   485 MET   486 HEM A 600
005 | 009 | 000007.0450 | 00000006854038.5000 | VAL   115 PHE   116 PHE   215 ILE   218 MET   306 ILE   309 GLU   313
THR   314 PRO   374 SER   375 LEU   379 SER   485 MET   486
002 | 006 | 000007.0430 | 00000006877214.0000 | LEU   51 TYR   52 VAL   219 SER   375 PRO   376 LEU   377 LEU   378
LEU   379 PRO   380 LEU   401 GLY   482 ALA   483 ALA   484 MET   486
002 | 007 | 000007.0400 | 00000006912125.0000 | VAL   115 PHE   116 PHE   215 ILE   218 MET   306 ILE   309 GLU   313
THR   314 SER   375 LEU   379 SER   485 HEM A 600
005 | 010 | 000007.0320 | 00000007006089.0000 | VAL   115 PHE   116 PHE   215 ILE   218 ALA   310 THR   314 PRO   374
SER   375 LEU   379 CYS   445 ALA   484 SER   485 HEM A 600

003 | 008 | 000006.9770 | 00000007687607.5000 | VAL   115 PHE   116 PHE   215 ILE   218 MET   306 ILE   309 ALA   310
GLU   313 THR   314 LEU   379 SER   485 HEM A 600
005 | 011 | 000006.9750 | 00000007713601.5000 | VAL   115 PHE   116 PHE   215 ILE   218 MET   306 ILE   309 ALA   310
GLU   313 THR   314 LEU   379 CYS   445 SER   485 HEM A 600
004 | 011 | 000006.9350 | 00000008252349.0000 | VAL   115 PHE   116 PHE   215 ILE   218 MET   306 ILE   309 GLU   313
THR   314 ALA   317 PRO   374 SER   375 LEU   379 SER   485 MET   486
005 | 012 | 000006.9270 | 00000008364532.5000 | VAL   115 PHE   116 PHE   215 ILE   218 MET   306 ILE   309 ALA   310
THR   314 PRO   374 SER   375 LEU   379 SER   485 HEM A 600
004 | 012 | 000006.9210 | 00000008449670.0000 | VAL   115 PHE   116 ILE   218 MET   306 ALA   310 THR   314 PRO   374
SER   375 PRO   376 LEU   379 CYS   445 ALA   484 SER   485 MET   486 HEM A 600
004 | 013 | 000006.9050 | 00000008680963.0000 | VAL   115 PHE   116 ILE   218 VAL   219 MET   306 ILE   309 ALA   310
THR   314 SER   375 LEU   378 LEU   379 CYS   445 ALA   484 SER   485 HEM A 600
004 | 014 | 000006.8640 | 00000009302963.0000 | VAL   115 PHE   116 PHE   215 ILE   218 MET   306 ILE   309 ALA   310
GLU   313 THR   314 LEU   379 SER   485 HEM A 600
004 | 015 | 000006.8590 | 00000009381804.0000 | PHE   116 ILE   218 MET   306 ILE   309 ALA   310 THR   314 PRO   374
SER   375 PRO   376 LEU   379 ALA   484 SER   485 MET   486
001 | 006 | 000006.8580 | 00000009397652.0000 | PHE   116 PHE   215 ILE   218 ILE   309 GLU   313 THR   314 SER   375
LEU   378 LEU   379 PRO   380 SER   485
002 | 008 | 000006.8560 | 00000009429428.0000 | PHE   116 ILE   218 MET   306 ILE   309 ALA   310 THR   314 SER   375
LEU   378 LEU   379 CYS   445 HEM A 600
005 | 013 | 000006.8350 | 00000009769640.0000 | PHE   116 PHE   215 ILE   218 ILE   309 ALA   310 GLU   313 THR   314
SER   375 LEU   379 CYS   445 HEM A 600
001 | 007 | 000006.8280 | 00000009885750.0000 | VAL   115 PHE   116 PHE   215 ILE   218 MET   306 ILE   309 GLU   313
THR   314 PRO   374 SER   375 LEU   379 SER   485 MET   486
001 | 008 | 000006.7980 | 00000010399200.0000 | VAL   115 PHE   116 ILE   218 VAL   219 MET   306 ILE   309 ALA   310
THR   314 SER   375 LEU   378 LEU   379 PRO   380 SER   485
005 | 014 | 000006.7230 | 00000011802545.0000 | VAL   115 PHE   116 PHE   215 ILE   218 MET   306 ILE   309 GLU   313
THR   314 PRO   374 SER   375 LEU   379 SER   485 MET   486
004 | 016 | 000006.7200 | 00000011862458.0000 | VAL   115 PHE   116 PHE   215 ILE   218 MET   306 ILE   309 GLU   313
THR   314 PRO   374 SER   375 LEU   379 SER   485 MET   486 HEM A 600
004 | 017 | 000006.7100 | 00000012064374.0000 | PHE   215 ILE   218 ILE   309 ALA   310 GLU   313 THR   314 SER   375
LEU   378 LEU   379 SER   485 HEM A 600
005 | 015 | 000006.6950 | 00000012373710.0000 | PHE   116 PHE   215 ILE   218 ILE   309 GLU   313 THR   314 ALA   317
PRO   374 SER   375 LEU   378 LEU   379 PRO   380 SER   485 MET   486
004 | 018 | 000006.6910 | 00000012457531.0000 | PHE   116 ILE   218 VAL   219 ILE   309 ALA   310 THR   314 PRO   374
SER   375 PRO   376 LEU   377 LEU   378 LEU   379 ALA   484 SER   485 MET   486
001 | 009 | 000006.6860 | 00000012563106.0000 | ILE   46 LEU   51 VAL   219 SER   375 PRO   376 LEU   377 LEU   378
PRO   380 ALA   483 ALA   484 SER   485 MET   486
002 | 009 | 000006.6760 | 00000012776948.0000 | ILE   46 LEU   51 TYR   52 VAL   219 PHE   226 SER   375 PRO   376 LEU
377 LEU   378 PRO   380 ALA   483 ALA   484 SER   485 MET   486
005 | 016 | 000006.6670 | 00000012972516.0000 | PHE   116 PHE   215 ILE   218 VAL   219 ILE   309 ALA   310 GLU   313
THR   314 SER   375 LEU   378 LEU   379 ALA   484
001 | 010 | 000006.6330 | 00000013738728.0000 | PHE   116 PHE   215 ILE   218 ILE   309 GLU   313 THR   314 SER   375
LEU   378 LEU   379 PRO   380 SER   485

005 | 017 | 000006.6260 | 00000013902010.0000 | VAL 115 PHE 116 PHE 215 ILE 218 MET 306 ILE 309 GLU 313
THR 314 ALA 317 PRO 374 SER 375 LEU 379 SER 485
002 | 010 | 000006.6110 | 00000014258464.0000 | PHE 116 PHE 215 ILE 218 ILE 309 ALA 310 GLU 313 THR 314
PRO 374 SER 375 LEU 378 LEU 379 PRO 380 ALA 484 SER 485 MET 486
003 | 009 | 000006.6020 | 00000014476709.0000 | PHE 116 PHE 215 ILE 218 ILE 309 ALA 310 GLU 313 THR 314
PRO 374 SER 375 LEU 378 LEU 379 PRO 380 ALA 484 SER 485 MET 486
004 | 019 | 000006.5660 | 00000015383607.0000 | ILE 46 LEU 51 VAL 219 PHE 226 SER 375 PRO 376 LEU 377
LEU 378 PRO 380 ALA 483 ALA 484 SER 485
005 | 018 | 000006.5490 | 00000015831401.0000 | PHE 116 PHE 215 ILE 218 ILE 309 ALA 310 GLU 313 THR 314
SER 375 LEU 378 LEU 379 HEM A 600
004 | 020 | 000006.5450 | 00000015938645.0000 | LEU 51 PHE 116 ILE 218 VAL 219 SER 375 PRO 376 LEU 377
LEU 378 LEU 379 ALA 484 SER 485
005 | 019 | 000006.5140 | 00000016794794.0000 | PHE 116 PHE 215 ILE 218 ILE 309 ALA 310 GLU 313 THR 314
PRO 374 SER 375 LEU 378 LEU 379 PRO 380 ALA 484 SER 485 MET 486
002 | 011 | 000006.4990 | 00000017225420.0000 | PHE 116 ILE 218 VAL 219 MET 306 ILE 309 ALA 310 THR 314
SER 375 LEU 378 LEU 379 ALA 484
003 | 010 | 000006.4820 | 00000017726826.0000 | ILE 46 LEU 51 VAL 219 PHE 226 SER 375 PRO 376 LEU 377
LEU 378 PRO 380 ALA 483 ALA 484 SER 485 MET 486
005 | 020 | 000006.4770 | 00000017877058.0000 | ILE 46 LEU 51 VAL 219 PHE 226 SER 375 PRO 376 LEU 377
LEU 378 PRO 380 ALA 483 ALA 484 SER 485 MET 486
002 | 012 | 000006.4550 | 00000018553346.0000 | LEU 51 VAL 219 PRO 374 SER 375 PRO 376 LEU 377 LEU 378
LEU 379 PRO 380 LEU 401 ALA 483 ALA 484 SER 485 MET 486
003 | 011 | 000006.4490 | 00000018742190.0000 | PHE 116 PHE 215 ILE 218 MET 306 ILE 309 ALA 310 GLU 313
THR 314 SER 375 LEU 378 LEU 379 PRO 380 SER 485
001 | 011 | 000006.4320 | 00000019287746.0000 | LEU 51 TYR 52 VAL 219 PRO 374 SER 375 PRO 376 LEU 377
LEU 378 LEU 379 PRO 380 LEU 401 ALA 483 ALA 484 SER 485 MET 486
002 | 013 | 000006.4110 | 00000019983644.0000 | PHE 116 PHE 215 ILE 218 VAL 219 ILE 309 ALA 310 GLU 313
THR 314 PRO 374 SER 375 PRO 376 LEU 377 LEU 378 LEU 379 PRO 380 ALA 484 SER 485 MET 486
002 | 014 | 000006.4050 | 00000020187044.0000 | PHE 116 PHE 215 ILE 218 MET 306 ILE 309 ALA 310 GLU 313
THR 314 SER 375 LEU 378 LEU 379 PRO 380 SER 485
003 | 012 | 000006.3990 | 00000020392516.0000 | PHE 116 PHE 215 ILE 218 MET 306 ILE 309 GLU 313 THR 314
PRO 374 SER 375 LEU 378 LEU 379 PRO 380 ALA 484 SER 485
003 | 013 | 000006.3890 | 00000020739626.0000 | VAL 115 PHE 116 PHE 215 ILE 218 MET 306 ILE 309 GLU 313
THR 314 PRO 374 SER 375 LEU 379 SER 485
003 | 014 | 000006.3730 | 00000021307332.0000 | VAL 115 PHE 116 PHE 215 ILE 218 MET 306 ILE 309 GLU 313
THR 314 PRO 374 SER 375 LEU 379 SER 485
001 | 012 | 000006.3700 | 00000021415494.0000 | VAL 115 PHE 116 PHE 215 MET 306 ILE 309 ALA 310 GLU 313
THR 314 PRO 374 SER 375 PRO 376 LEU 377 LEU 378 LEU 379 PRO 380 ALA 484 SER 485 MET 486
003 | 015 | 000006.3550 | 00000021964596.0000 | VAL 115 PHE 116 PHE 215 MET 306 ILE 309 GLU 313 THR 314
ALA 317 PRO 374 SER 375 LEU 379 SER 485 MET 486 SER 487 HEM A 600
003 | 016 | 000006.3440 | 00000022376200.0000 | LEU 51 PHE 116 ILE 218 VAL 219 MET 306 ALA 310 THR 314
PRO 374 SER 375 PRO 376 LEU 377 LEU 378 LEU 379 ALA 484 SER 485 MET 486
003 | 017 | 000006.3130 | 00000023578146.0000 | LEU 51 PHE 116 ILE 218 VAL 219 SER 375 PRO 376 LEU 377
LEU 378 LEU 379 ALA 484 SER 485

```
001 | 013 |         000006.3100 | 00000023697836.0000 | PHE   215 ILE   218 VAL   219 ILE   309 ALA   310 GLU   313 THR   314
SER   375 LEU   378 LEU   379 PRO   380 ALA   484 SER   485
002 | 015 |         000006.2560 | 00000025959194.0000 | LEU   51 TYR   52 PHE   116 VAL   219 SER   375 PRO   376 LEU   377
LEU   378 LEU   379 PRO   380 ALA   483 ALA   484
002 | 016 |         000006.2550 | 00000026003044.0000 | LEU   51 PHE   116 ILE   218 VAL   219 SER   375 PRO   376 LEU   377
LEU   378 LEU   379 ALA   484
001 | 014 |         000006.2230 | 00000027446092.0000 | VAL   115 PHE   116 PHE   215 ILE   218 MET   306 ILE   309 ALA   310
THR   314 PRO   374 SER   375 LEU   377 LEU   378 LEU   379 PRO   380 ALA   484 SER   485 MET   486
002 | 017 |         000006.2170 | 00000027725448.0000 | ILE   46 LEU   51 TYR   52 VAL   219 SER   375 PRO   376 LEU   377 LEU
378 ALA   483 ALA   484 SER   485 MET   486
003 | 018 |         000006.1790 | 00000029561940.0000 | LEU   51 PHE   116 PHE   215 ILE   218 VAL   219 SER   375 PRO   376
LEU   377 LEU   378 LEU   379 PRO   380 ALA   484 SER   485
001 | 015 |         000006.1740 | 00000029812472.0000 | LEU   51 VAL   115 PHE   116 ILE   218 VAL   219 SER   375 PRO   376
LEU   377 LEU   378 LEU   379 ALA   484
001 | 016 |         000006.1690 | 00000030065128.0000 | LEU   51 VAL   115 PHE   116 MET   306 PRO   374 SER   375 PRO   376
LEU   377 LEU   378 LEU   379 PRO   380 ALA   484 SER   485 MET   486
001 | 017 |         000006.1680 | 00000030115914.0000 | VAL   115 PHE   116 PHE   215 MET   306 ILE   309 ALA   310 GLU   313
THR   314 ALA   317 PRO   374 SER   375 LEU   379 SER   485 MET   486 HEM A 600
002 | 018 |         000006.1590 | 00000030576878.0000 | ILE   46 LYS   47 PRO   48 GLY   49 PRO   50 LEU   51 VAL   219 PHE
226 ALA   483 ALA   484
003 | 019 |         000006.1550 | 00000030784010.0000 | ILE   46 LYS   47 PRO   48 GLY   49 PRO   50 LEU   51 VAL   219 ALA
483 ALA   484 SER   485
001 | 018 |         000006.1100 | 00000033213192.0000 | LEU   51 THR   101 PHE   116 ILE   218 VAL   219 PRO   374 SER   375
PRO   376 LEU   377 LEU   378 LEU   379 PRO   380 ALA   484 SER   485 MET   486
002 | 019 |         000006.1100 | 00000033213192.0000 | ILE   46 LEU   51 TYR   52 VAL   219 PRO   374 SER   375 PRO   376 LEU
377 LEU   378 GLY   482 ALA   483 ALA   484 SER   485 MET   486
002 | 020 |         000006.1010 | 00000033721564.0000 | LEU   51 PHE   116 ILE   218 VAL   219 PRO   374 SER   375 PRO   376
LEU   377 LEU   378 LEU   379 PRO   380 ALA   484 SER   485 MET   486
003 | 020 |         000006.0880 | 00000034469648.0000 | PHE   116 PHE   215 ILE   218 MET   306 ILE   309 ALA   310 GLU   313
THR   314 PRO   374 SER   375 PRO   376 LEU   377 LEU   378 LEU   379 PRO   380 ALA   484 SER   485 MET   486
001 | 019 |         000006.0620 | 00000036015968.0000 | VAL   115 PHE   116 LEU   214 PHE   215 ILE   218 MET   306 ILE   309
GLU   313 THR   314 PRO   374 SER   375 LEU   379 SER   485 MET   486
001 | 020 |         000006.0360 | 00000037631656.0000 | VAL   115 PHE   116 ILE   218 VAL   219 MET   306 ILE   309 ALA   310
SER   375 LEU   378 LEU   379 ALA   484

After clustering the 100 runs, the following 9 distinct complex conformations were found:
[They all differ by at least 5.0 A heavy ligand atom RMSD, and have been saved as YOb files with terminal number "Num"]

Num | Rec | Run |Bind.energy[kcal/mol]|Dissoc. constant [pM]| Contacting receptor residues
----+-----+-----+---------------------+---------------------+----------------------------
001 | 002 | 001 |         000008.2560 | 00000000887711.3125 | VAL   115 PHE   116 PHE   215 ILE   218 MET   306 ILE   309 ALA
310 THR   314 PRO   374 SER   375 LEU   379 CYS   445 ALA   484 SER   485 MET   486 HEM A 600
002 | 001 | 002 |         000007.7200 | 00000002193638.0000 | VAL   115 PHE   116 ILE   218 VAL   219 MET   306 ALA   310 THR
314 PRO   374 SER   375 LEU   378 LEU   379 CYS   445 ALA   484 SER   485 HEM A 600
```

003 | 001 | 003 | 000007.5170 | 00000003090053.0000 | VAL 115 PHE 116 PHE 215 ILE 218 MET 306 ILE 309 GLU 313 THR 314 ALA 317 PRO 374 SER 375 LEU 379 SER 485 MET 486
004 | 002 | 003 | 000007.0430 | 00000006877214.0000 | LEU 51 TYR 52 VAL 219 SER 375 PRO 376 LEU 377 LEU 378 LEU 379 PRO 380 LEU 401 GLY 482 ALA 483 ALA 484 MET 486
005 | 005 | 003 | 000006.7230 | 00000011802545.0000 | VAL 115 PHE 116 PHE 215 ILE 218 MET 306 ILE 309 GLU 313 THR 314 PRO 374 SER 375 LEU 379 SER 485 MET 486
006 | 001 | 004 | 000006.6860 | 00000012563106.0000 | ILE 46 LEU 51 VAL 219 SER 375 PRO 376 LEU 377 LEU 378 PRO 380 ALA 483 ALA 484 SER 485 MET 486
007 | 004 | 004 | 000006.5450 | 00000015938645.0000 | LEU 51 PHE 116 ILE 218 VAL 219 SER 375 PRO 376 LEU 377 LEU 378 LEU 379 ALA 484 SER 485
008 | 003 | 005 | 000006.1790 | 00000029561940.0000 | LEU 51 PHE 116 PHE 215 ILE 218 VAL 219 SER 375 PRO 376 LEU 377 LEU 378 LEU 379 PRO 380 ALA 484 SER 485
009 | 002 | 006 | 000006.1590 | 00000030576878.0000 | ILE 46 LYS 47 PRO 48 GLY 49 PRO 50 LEU 51 VAL 219 PHE 226 ALA 483 ALA 484

c)CYP81AA2(mut6) rigid receptor, all 25 docking results

```
Global docking result analysis
==============================

25 VINA docking runs of the ligand object 2 to the receptor object 1 yielded the following results,
sorted by binding energy [more positive energies indicate stronger binding, and negative energies mean no binding]

Run |Bind.energy[kcal/mol]|Dissoc. constant [pM]| Contacting receptor residues
----+---------------------+---------------------+----------------------------
001 |          000008.4470 | 00000000643082.8125 | VAL   115 PHE   116 PHE   215 THR   305 MET   306 ILE   309 ALA   310 THR
314 PRO   374 ALA   375 LEU   379 SER   485 HEM A 600
002 |          000008.4470 | 00000000643082.8125 | VAL   115 PHE   116 PHE   215 THR   305 MET   306 ILE   309 ALA   310 THR
314 PRO   374 ALA   375 LEU   379 SER   485 HEM A 600
003 |          000008.2310 | 00000000925970.1875 | VAL   115 PHE   116 LEU   214 PHE   215 ILE   218 ARG   249 THR   305 MET
306 ILE   309 ALA   310 LEU   379 HEM A 600
004 |          000008.2310 | 00000000925970.1875 | VAL   115 PHE   116 LEU   214 PHE   215 ILE   218 ARG   249 THR   305 MET
306 ILE   309 ALA   310 LEU   379 HEM A 600
005 |          000008.0280 | 00000001304361.5000 | LEU   107 VAL   115 LEU   214 PHE   215 ILE   218 ILE   243 ARG   249 THR
305 MET   306 ILE   309 ALA   310 THR   314 ALA   484
006 |          000008.0280 | 00000001304361.5000 | LEU   107 VAL   115 LEU   214 PHE   215 ILE   218 ILE   243 ARG   249 THR
305 MET   306 ILE   309 ALA   310 THR   314 ALA   484
007 |          000008.0100 | 00000001344597.0000 | LEU   107 VAL   115 LEU   214 PHE   215 ILE   218 ILE   243 ARG   249 THR
305 MET   306 ILE   309 ALA   310 LEU   379 HEM A 600
008 |          000008.0100 | 00000001344597.0000 | LEU   107 VAL   115 LEU   214 PHE   215 ILE   218 ILE   243 ARG   249 THR
305 MET   306 ILE   309 ALA   310 LEU   379 HEM A 600
009 |          000008.0030 | 00000001360577.2500 | LEU   107 THR   108 PHE   113 VAL   115 PHE   116 LEU   214 PHE   215 ILE
218 ILE   243 ARG   249 THR   305 MET   306 ILE   309 ALA   310 THR   314
010 |          000008.0030 | 00000001360577.2500 | LEU   107 THR   108 PHE   113 VAL   115 PHE   116 LEU   214 PHE   215 ILE
218 ILE   243 ARG   249 THR   305 MET   306 ILE   309 ALA   310 THR   314
011 |          000007.9940 | 00000001381402.7500 | VAL   115 PHE   116 LEU   214 PHE   215 ILE   218 ILE   243 ARG   249 THR
305 MET   306 ILE   309 ALA   310 LEU   379 ALA   484 HEM A 600
012 |          000007.7560 | 00000002064318.1250 | LEU   107 THR   108 PHE   113 VAL   115 PHE   116 LEU   214 PHE   215 ILE
218 ARG   249 GLY   302 THR   305 MET   306 ILE   309 ALA   310 LEU   379
013 |          000007.6370 | 00000002523508.5000 | LEU   107 PHE   113 VAL   115 PHE   116 PHE   215 ILE   218 THR   305 MET
306 ILE   309 ALA   310 LEU   379 ALA   484 HEM A 600
014 |          000007.6230 | 00000002583847.7500 | GLY    49 PRO    50 LEU    51 TYR    52 VAL   219 PRO   376 LEU   377 SER   378
LEU   379 PRO   380 LEU   401 ALA   481 GLY   482 THR   483 ALA   484
015 |          000007.5370 | 00000002987485.0000 | VAL   115 LEU   214 PHE   215 ILE   218 ILE   243 ARG   249 THR   305 MET
306 ILE   309 ALA   310 LEU   379 ALA   484 HEM A 600
016 |          000007.4010 | 00000003758331.2500 | VAL   115 PHE   116 LEU   214 PHE   215 ILE   218 THR   305 MET   306 ILE
309 ALA   310 ALA   375 LEU   379 ALA   484 SER   485 HEM A 600
017 |          000007.3400 | 00000004165898.2500 | PHE   113 VAL   115 PHE   116 ILE   218 THR   305 MET   306 ILE   309 ALA
310 THR   314 ALA   375 LEU   379 ALA   484 HEM A 600
```

```
018 |         000007.2920 | 00000004517447.5000 | GLY   49 PRO   50 LEU   51 TYR   52 VAL   219 ALA   375 PRO   376 LEU   377
SER   378 LEU   379 ALA   481 GLY   482 THR   483 ALA   484
019 |         000007.2890 | 00000004540379.5000 | LEU   107 PHE   113 VAL   115 PHE   116 PHE   215 ILE   218 THR   305 MET
306 ILE   309 ALA   310 THR   314 ALA   484 SER   485
020 |         000007.2020 | 00000005258525.5000 | LEU   107 PHE   113 VAL   115 PHE   116 LEU   214 PHE   215 ILE   218 ILE
243 GLY   246 ARG   249 THR   305 MET   306 ILE   309 ALA   310 LEU   379 ALA   484
021 |         000007.1530 | 00000005711911.5000 | PHE   116 PHE   215 ILE   218 ILE   309 ALA   310 GLU   313 THR   314 ALA
375 SER   378 LEU   379 PRO   380 ALA   484 SER   485
022 |         000007.0800 | 00000006460873.0000 | PHE   116 PHE   215 ILE   218 ILE   309 ALA   310 THR   314 ALA   375 LEU
379 ALA   484 HEM A 600
023 |         000007.0090 | 00000007283412.0000 | PHE   116 ILE   218 ILE   309 ALA   310 THR   314 ALA   375 SER   378 LEU
379 ALA   484 SER   485
024 |         000006.9850 | 00000007584502.5000 | VAL   115 PHE   116 PHE   215 ILE   218 THR   305 MET   306 ILE   309 ALA
310 THR   314 ALA   375 ALA   484 SER   485
025 |         000006.8740 | 00000009147264.0000 | VAL   115 PHE   116 ILE   218 MET   306 ILE   309 ALA   310 THR   314 ALA
375 SER   378 LEU   379 PRO   380 ALA   484

After clustering the 25 runs, the following 7 distinct complex conformations were found:
[They all differ by at least 5.0 A heavy atom RMSD]

Clu |Bind.energy[kcal/mol]|Dissoc. constant [pM]| Contacting receptor residues
----+---------------------+---------------------+----------------------------
001 |         000008.4470 | 00000000643082.8125 | VAL   115 PHE   116 PHE   215 THR   305 MET   306 ILE   309 ALA   310 THR
314 PRO   374 ALA   375 LEU   379 SER   485 HEM A 600
002 |         000008.2310 | 00000000925970.1875 | VAL   115 PHE   116 LEU   214 PHE   215 ILE   218 ARG   249 THR   305 MET
306 ILE   309 ALA   310 LEU   379 HEM A 600
003 |         000007.7560 | 00000002064318.1250 | LEU   107 THR   108 PHE   113 VAL   115 PHE   116 LEU   214 PHE   215 ILE
218 ARG   249 GLY   302 THR   305 MET   306 ILE   309 ALA   310 LEU   379
004 |         000007.6230 | 00000002583847.7500 | GLY   49 PRO   50 LEU   51 TYR   52 VAL   219 PRO   376 LEU   377 SER   378
LEU   379 PRO   380 LEU   401 ALA   481 GLY   482 THR   483 ALA   484
005 |         000007.2920 | 00000004517447.5000 | GLY   49 PRO   50 LEU   51 TYR   52 VAL   219 ALA   375 PRO   376 LEU   377
SER   378 LEU   379 ALA   481 GLY   482 THR   483 ALA   484
006 |         000007.1530 | 00000005711911.5000 | PHE   116 PHE   215 ILE   218 ILE   309 ALA   310 GLU   313 THR   314 ALA
375 SER   378 LEU   379 PRO   380 ALA   484 SER   485
007 |         000006.8740 | 00000009147264.0000 | VAL   115 PHE   116 ILE   218 MET   306 ILE   309 ALA   310 THR   314 ALA
375 SER   378 LEU   379 PRO   380 ALA   484
```

d) CYP81AA2(mut6) flexible receptor sidechains, all 100 docking results

Ensemble docking result analysis
================================

The ligand was docked 20 times ["Run"] with VINA against each of the 5 receptors ["Rec"] in the ensemble, yielding the following 100 results, sorted by binding energy:
[More positive energies indicate stronger binding, and negative energies mean no binding]

```
Rec | Run |Bind.energy[kcal/mol]|Dissoc. constant [pM]| Contacting receptor residues
----+-----+---------------------+---------------------+----------------------------
001 | 001 |         000008.4390 | 00000000651824.9375 | VAL   115 PHE   116 PHE   215 THR   305 MET   306 ILE   309 ALA   310
THR   314 PRO   374 ALA   375 LEU   379 SER   485 HEM A 600
001 | 002 |         000008.2390 | 00000000913551.2500 | VAL   115 PHE   116 LEU   214 PHE   215 ILE   218 ARG   249 THR   305
MET   306 ILE   309 ALA   310 LEU   379 HEM A 600
001 | 003 |         000008.0320 | 00000001295585.1250 | LEU   107 VAL   115 LEU   214 PHE   215 ILE   218 ILE   243 ARG   249
THR   305 MET   306 ILE   309 ALA   310 THR   314 ALA   484
001 | 004 |         000008.0020 | 00000001362875.6250 | LEU   107 VAL   115 LEU   214 PHE   215 ILE   218 ILE   243 ARG   249
THR   305 MET   306 ILE   309 ALA   310 LEU   379 HEM A 600
001 | 005 |         000008.0020 | 00000001362875.6250 | LEU   107 VAL   115 PHE   116 LEU   214 PHE   215 ILE   218 ILE   243
ARG   249 THR   305 MET   306 ILE   309 ALA   310 LEU   379 ALA   484 HEM A 600
001 | 006 |         000007.9960 | 00000001376747.5000 | LEU   107 THR   108 PHE   113 VAL   115 PHE   116 LEU   214 PHE   215
ILE   218 ILE   243 ARG   249 THR   305 MET   306 ILE   309 ALA   310 THR   314
001 | 007 |         000007.9070 | 00000001599897.5000 | VAL   115 PHE   116 PHE   215 ILE   218 THR   305 MET   306 ILE   309
ALA   310 ALA   375 LEU   379 ALA   484 HEM A 600
001 | 008 |         000007.8600 | 00000001731982.8750 | LEU   107 PHE   113 VAL   115 PHE   116 LEU   214 PHE   215 ILE   218
ILE   243 ARG   249 THR   305 MET   306 ILE   309 ALA   310
004 | 001 |         000007.8340 | 00000001809680.2500 | ARG   98 PRO   99 LYS   100 THR   101 SER   103 GLY   104 PHE   116
ILE   218 VAL   219 LEU   379 PRO   380
001 | 009 |         000007.7610 | 00000002046970.3750 | PHE   113 VAL   115 PHE   116 PHE   215 ILE   218 THR   305 MET   306
ILE   309 ALA   310 THR   314 SER   485 HEM A 600
002 | 001 |         000007.7520 | 00000002078302.0000 | THR   101 SER   103 GLY   104 LEU   107 PHE   113 PHE   116 LEU   214
PRO   217 ILE   218 VAL   219 PRO   223 ILE   243 ARG   249 THR   305 MET   306 ILE   309
003 | 001 |         000007.7520 | 00000002078302.0000 | THR   101 SER   103 GLY   104 LEU   107 PHE   113 PHE   116 LEU   214
PRO   217 ILE   218 VAL   219 PRO   223 ILE   243 ARG   249 THR   305 MET   306 ILE   309
005 | 001 |         000007.7280 | 00000002164217.5000 | VAL   115 PHE   215 ILE   218 MET   306 ALA   310 THR   314 ALA   375
LEU   379 CYS   445 PRO   446 ALA   484 SER   485 HEM A 600
001 | 010 |         000007.7140 | 00000002215965.7500 | VAL   115 PHE   116 PHE   215 MET   306 ALA   310 THR   314 PRO   374
ALA   375 LEU   379 ALA   484 SER   485 HEM A 600
001 | 011 |         000007.7010 | 00000002265125.0000 | LEU   107 THR   108 PHE   113 VAL   115 PHE   116 LEU   214 PHE   215
ILE   218 ARG   249 GLY   302 THR   305 MET   306 ILE   309 ALA   310 LEU   379
001 | 012 |         000007.6870 | 00000002319286.0000 | LEU   107 VAL   115 PHE   116 PHE   215 ILE   218 THR   305 MET   306
ILE   309 ALA   310 LEU   379 ALA   484 HEM A 600
001 | 013 |         000007.6450 | 00000002489663.7500 | LEU   107 PHE   113 VAL   115 LEU   214 PHE   215 ILE   218 ILE   243
ARG   249 THR   305 MET   306 ILE   309 ALA   310 LEU   379 ALA   484
```

001 | 014 | 000007.6280 | 00000002562134.2500 | GLY 49 PRO 50 LEU 51 TYR 52 VAL 219 PRO 376 LEU 377 SER
378 LEU 379 PRO 380 LEU 401 ALA 481 GLY 482 THR 483 ALA 484
005 | 002 | 000007.5590 | 00000002878588.2500 | THR 101 SER 103 GLY 104 LEU 107 PHE 113 VAL 115 PHE 116
LEU 214 PRO 217 ILE 218 PRO 223 ILE 242 ILE 243 MET 306 ILE 309
002 | 002 | 000007.5430 | 00000002957383.7500 | THR 101 SER 103 GLY 104 LEU 107 PHE 113 PHE 116 LEU 214
PRO 217 ILE 218 PRO 223 ILE 243 ARG 249 THR 305 ILE 309
003 | 002 | 000007.5430 | 00000002957383.7500 | THR 101 SER 103 GLY 104 LEU 107 PHE 113 PHE 116 LEU 214
PRO 217 ILE 218 PRO 223 ILE 243 ARG 249 THR 305 ILE 309
005 | 003 | 000007.5400 | 00000002972396.2500 | VAL 115 PHE 215 ILE 218 MET 306 ALA 310 THR 314 PRO 374
ALA 375 LEU 379 ALA 484 SER 485 HEM A 600
004 | 002 | 000007.5020 | 00000003169283.5000 | VAL 115 PHE 116 PHE 215 ILE 218 MET 306 ALA 310 THR 314
PRO 374 ALA 375 LEU 379 ALA 484 SER 485 HEM A 600
004 | 003 | 000007.4920 | 00000003223229.2500 | ARG 98 PRO 99 LYS 100 THR 101 GLY 104 THR 108 PHE 116
ILE 218 VAL 219 PRO 380
002 | 003 | 000007.4880 | 00000003245063.7500 | LYS 173 LYS 212 PHE 215 PHE 216 VAL 219 GLN 480 GLY 482
THR 483 SER 487
003 | 003 | 000007.4880 | 00000003245063.7500 | LYS 173 LYS 212 PHE 215 PHE 216 VAL 219 GLN 480 GLY 482
THR 483 SER 487
005 | 004 | 000007.4870 | 00000003250545.5000 | LYS 173 LYS 212 PHE 215 PHE 216 ILE 218 VAL 219 GLN 480
GLY 482 THR 483 ALA 484 SER 487
005 | 005 | 000007.4820 | 00000003278093.0000 | THR 101 SER 103 GLY 104 LEU 107 PHE 113 PHE 116 LEU 214
PRO 217 ILE 218 PRO 223 ILE 243 ARG 249 THR 305 MET 306 ILE 309
004 | 004 | 000007.4770 | 00000003305874.2500 | LYS 173 LYS 212 PHE 215 PHE 216 ILE 218 VAL 219 GLN 480
GLY 482 THR 483 SER 487
002 | 004 | 000007.4380 | 00000003530804.7500 | THR 101 SER 103 GLY 104 LEU 107 PHE 116 LEU 214 PRO 217
ILE 218 PRO 223 ILE 242 ILE 243 GLY 246 ARG 249 ILE 309
003 | 004 | 000007.4380 | 00000003530804.7500 | THR 101 SER 103 GLY 104 LEU 107 PHE 116 LEU 214 PRO 217
ILE 218 PRO 223 ILE 242 ILE 243 GLY 246 ARG 249 ILE 309
002 | 005 | 000007.3910 | 00000003822303.5000 | VAL 115 PHE 116 PHE 215 ILE 218 MET 306 ALA 310 THR 314
PRO 374 ALA 375 LEU 379 ALA 484 SER 485 HEM A 600
003 | 005 | 000007.3910 | 00000003822303.5000 | VAL 115 PHE 116 PHE 215 ILE 218 MET 306 ALA 310 THR 314
PRO 374 ALA 375 LEU 379 ALA 484 SER 485 HEM A 600
002 | 006 | 000007.3300 | 00000004236808.0000 | PHE 116 PHE 215 ILE 218 MET 306 ILE 309 ALA 310 THR 314
ALA 375 LEU 379 CYS 445 ALA 484 HEM A 600
003 | 006 | 000007.3300 | 00000004236808.0000 | PHE 116 PHE 215 ILE 218 MET 306 ILE 309 ALA 310 THR 314
ALA 375 LEU 379 CYS 445 ALA 484 HEM A 600
002 | 007 | 000007.3280 | 00000004251134.0000 | VAL 115 PHE 116 PHE 215 ILE 218 MET 306 ALA 310 THR 314
PRO 374 ALA 375 LEU 379 ALA 484 SER 485 HEM A 600
003 | 007 | 000007.3280 | 00000004251134.0000 | VAL 115 PHE 116 PHE 215 ILE 218 MET 306 ALA 310 THR 314
PRO 374 ALA 375 LEU 379 ALA 484 SER 485 HEM A 600
001 | 015 | 000007.3250 | 00000004272714.0000 | LEU 107 PHE 113 VAL 115 PHE 116 PHE 215 ILE 218 MET 306
ILE 309 ALA 310 THR 314 ALA 484 SER 485
004 | 005 | 000007.3240 | 00000004279931.5000 | GLY 49 PRO 50 LEU 51 TYR 52 VAL 219 ALA 375 PRO 376 LEU
377 SER 378 LEU 379 ALA 481 GLY 482 THR 483 ALA 484

002 | 008 | 000007.3220 | 00000004294403.5000 | PRO 48 GLY 49 PRO 50 LEU 51 TYR 52 VAL 219 ALA 375 PRO 376 LEU 377 SER 378 LEU 379 ALA 481 GLY 482 THR 483 ALA 484
003 | 008 | 000007.3220 | 00000004294403.5000 | PRO 48 GLY 49 PRO 50 LEU 51 TYR 52 VAL 219 ALA 375 PRO 376 LEU 377 SER 378 LEU 379 ALA 481 GLY 482 THR 483 ALA 484
005 | 006 | 000007.3170 | 00000004330797.5000 | PRO 48 GLY 49 PRO 50 LEU 51 TYR 52 VAL 219 ALA 375 PRO 376 LEU 377 SER 378 LEU 379 ALA 481 GLY 482 THR 483 ALA 484
001 | 016 | 000007.3140 | 00000004352782.0000 | LEU 107 THR 108 PHE 113 VAL 115 PHE 116 PHE 215 ILE 218 ARG 249 GLY 302 THR 305 MET 306 ILE 309 ALA 310 ALA 484
001 | 017 | 000007.3000 | 00000004456860.5000 | VAL 115 PHE 116 ILE 218 ILE 309 ALA 310 THR 314 ALA 375 LEU 379 ALA 484 HEM A 600
001 | 018 | 000007.3000 | 00000004456860.5000 | PRO 48 GLY 49 PRO 50 LEU 51 TYR 52 VAL 219 ALA 375 PRO 376 LEU 377 SER 378 LEU 379 ALA 481 GLY 482 THR 483 ALA 484 SER 485
002 | 009 | 000007.2530 | 00000004824813.0000 | GLY 49 PRO 50 LEU 51 TYR 52 ILE 218 VAL 219 PRO 376 LEU 377 SER 378 LEU 379 PRO 380 ALA 481 GLY 482 THR 483 ALA 484
003 | 009 | 000007.2530 | 00000004824813.0000 | GLY 49 PRO 50 LEU 51 TYR 52 ILE 218 VAL 219 PRO 376 LEU 377 SER 378 LEU 379 PRO 380 ALA 481 GLY 482 THR 483 ALA 484
002 | 010 | 000007.2480 | 00000004865702.5000 | LEU 107 PHE 116 PHE 215 ILE 218 MET 306 ILE 309 ALA 310 GLU 313 THR 314 ALA 375 LEU 379 ALA 484 SER 485
003 | 010 | 000007.2480 | 00000004865702.5000 | LEU 107 PHE 116 PHE 215 ILE 218 MET 306 ILE 309 ALA 310 GLU 313 THR 314 ALA 375 LEU 379 ALA 484 SER 485
005 | 007 | 000007.2390 | 00000004940178.5000 | GLY 49 PRO 50 LEU 51 TYR 52 VAL 219 PRO 376 LEU 377 SER 378 LEU 379 PRO 380 ALA 481 GLY 482 THR 483 ALA 484
004 | 006 | 000007.2140 | 00000005153092.0000 | THR 101 SER 103 GLY 104 LEU 107 PHE 113 PHE 116 LEU 214 PRO 217 ILE 218 PRO 223 ILE 243 ARG 249 THR 305 MET 306 ILE 309
005 | 008 | 000007.2040 | 00000005240805.0000 | LYS 173 LYS 212 PHE 215 PHE 216 ILE 218 VAL 219 GLN 480 GLY 482 THR 483 ALA 484 SER 487 PRO 491
002 | 011 | 000007.2020 | 00000005258525.5000 | LYS 173 LYS 212 PHE 215 PHE 216 VAL 219 GLN 480 GLY 482 THR 483 SER 487 PRO 491
003 | 011 | 000007.2020 | 00000005258525.5000 | LYS 173 LYS 212 PHE 215 PHE 216 VAL 219 GLN 480 GLY 482 THR 483 SER 487 PRO 491
004 | 007 | 000007.2010 | 00000005267408.5000 | VAL 115 PHE 116 PHE 215 ILE 218 MET 306 ALA 310 THR 314 ALA 375 LEU 379 ALA 484 SER 485 HEM A 600
004 | 008 | 000007.1960 | 00000005312049.0000 | LYS 173 LYS 212 PHE 215 PHE 216 ILE 218 VAL 219 GLN 480 GLY 482 THR 483 SER 487 PRO 491
004 | 009 | 000007.0910 | 00000006342027.0000 | PHE 116 PHE 215 ILE 218 MET 306 ILE 309 ALA 310 THR 314 ALA 375 LEU 379 CYS 445 ALA 484 HEM A 600
005 | 009 | 000007.0900 | 00000006352740.0000 | THR 101 SER 103 LEU 107 PHE 113 VAL 115 PHE 116 LEU 214 PRO 217 ILE 218 VAL 219 PRO 223 ILE 243 ARG 249 THR 305 MET 306 ILE 309
004 | 010 | 000007.0800 | 00000006460873.0000 | PHE 116 PHE 215 ILE 218 MET 306 ILE 309 ALA 310 GLU 313 THR 314 ALA 375 LEU 379 ALA 484 SER 485
001 | 019 | 000007.0770 | 00000006493670.0000 | LEU 107 PHE 113 VAL 115 PHE 116 LEU 214 PHE 215 ILE 218 ILE 243 ARG 249 MET 306 ILE 309 ALA 310
004 | 011 | 000007.0370 | 00000006947213.0000 | THR 101 SER 103 LEU 107 PHE 116 LEU 214 PRO 217 ILE 218 PRO 223 ILE 242 ILE 243 ARG 249 MET 306 ILE 309

005 | 010 | 000007.0090 | 00000007283412.0000 | THR 101 SER 103 GLY 104 LEU 107 PHE 113 PHE 116 LEU 214
PRO 217 ILE 218 PRO 223 ILE 243 ARG 249 THR 305 ILE 309
004 | 012 | 000006.9740 | 00000007726632.0000 | THR 101 SER 103 GLY 104 LEU 107 PHE 116 LEU 214 PRO 217
ILE 218 PRO 223 ILE 242 ILE 243 ARG 249
005 | 011 | 000006.9730 | 00000007739684.0000 | ARG 98 LEU 107 PHE 113 VAL 115 PHE 116 LEU 214 ILE 218
ILE 243 GLY 246 ARG 249 THR 305 MET 306 ILE 309
002 | 012 | 000006.9670 | 00000007818461.0000 | LYS 173 PHE 215 PHE 216 ILE 218 GLU 313 GLN 480 GLY 482
THR 483 ALA 484 SER 485 SER 487 PRO 491
003 | 012 | 000006.9670 | 00000007818461.0000 | LYS 173 PHE 215 PHE 216 ILE 218 GLU 313 GLN 480 GLY 482
THR 483 ALA 484 SER 485 SER 487 PRO 491
005 | 012 | 000006.9620 | 00000007884721.0000 | LYS 173 PHE 215 PHE 216 GLU 313 GLN 480 GLY 482 THR 483
ALA 484 SER 485 SER 487 PRO 491
005 | 013 | 000006.9550 | 00000007978429.5000 | THR 101 GLY 104 LEU 107 PHE 113 VAL 115 PHE 116 LEU 214
ILE 218 ILE 243 GLY 246 ARG 249 MET 306 ILE 309 LEU 379 PRO 380
004 | 013 | 000006.9460 | 00000008100549.5000 | LEU 107 THR 108 PHE 113 VAL 115 PHE 116 LEU 214 PHE 215
ILE 218 ARG 249 THR 305 MET 306 ILE 309 ALA 310 LEU 379
005 | 014 | 000006.9270 | 00000008364532.5000 | LYS 100 THR 101 SER 103 GLY 104 LEU 107 PHE 116 LEU 214
ILE 218 ILE 243 GLY 246 ARG 249 MET 306 ILE 309 LEU 379
005 | 015 | 000006.8140 | 00000010122127.0000 | THR 101 SER 103 GLY 104 LEU 107 THR 108 PHE 113 PHE 116
LEU 214 ILE 218 ILE 243 GLY 246 ARG 249 THR 305 MET 306 ILE 309
001 | 020 | 000006.8080 | 00000010225153.0000 | LEU 107 VAL 115 PHE 116 LEU 214 ILE 218 ILE 243 ARG 249
THR 305 MET 306 ILE 309 ALA 310 THR 314 LEU 379 ALA 484
004 | 014 | 000006.7970 | 00000010416767.0000 | LEU 107 PHE 116 LEU 214 PHE 215 ILE 218 ILE 243 ARG 249
THR 305 MET 306 ILE 309 ALA 310 THR 314 LEU 379 ALA 484 SER 485
002 | 013 | 000006.7900 | 00000010540568.0000 | PRO 48 GLY 49 PRO 50 LEU 51 TYR 52 VAL 219 ALA 375 PRO
376 LEU 377 SER 378 LEU 379 PRO 380 GLY 482 THR 483 ALA 484
003 | 013 | 000006.7900 | 00000010540568.0000 | PRO 48 GLY 49 PRO 50 LEU 51 TYR 52 VAL 219 ALA 375 PRO
376 LEU 377 SER 378 LEU 379 PRO 380 GLY 482 THR 483 ALA 484
005 | 016 | 000006.7710 | 00000010884067.0000 | ARG 98 GLY 104 LEU 107 PHE 113 VAL 115 PHE 116 LEU 214
ILE 218 ILE 243 ARG 249 THR 305 MET 306 ILE 309 LEU 379 PRO 380
004 | 015 | 000006.7610 | 00000011069329.0000 | LYS 173 PHE 211 LYS 212 PHE 215 PHE 216 VAL 219 GLU 313
GLN 480 GLY 482 THR 483 SER 487 ARG 488
002 | 014 | 000006.7530 | 00000011219806.0000 | THR 101 SER 103 GLY 104 LEU 107 THR 108 PHE 113 PHE 116
ILE 218 GLY 302 THR 305 MET 306 ILE 309
003 | 014 | 000006.7530 | 00000011219806.0000 | THR 101 SER 103 GLY 104 LEU 107 THR 108 PHE 113 PHE 116
ILE 218 GLY 302 THR 305 MET 306 ILE 309
002 | 015 | 000006.7370 | 00000011526926.0000 | THR 101 SER 103 GLY 104 LEU 107 THR 108 PHE 113 PHE 116
LEU 214 PRO 217 ILE 218 PRO 223 ILE 242 ILE 243 THR 305 ILE 309
003 | 015 | 000006.7370 | 00000011526926.0000 | THR 101 SER 103 GLY 104 LEU 107 THR 108 PHE 113 PHE 116
LEU 214 PRO 217 ILE 218 PRO 223 ILE 242 ILE 243 THR 305 ILE 309
005 | 017 | 000006.7040 | 00000012187169.0000 | ARG 98 PRO 99 LYS 100 THR 101 SER 103 GLY 104 LEU 107
PHE 113 VAL 115 PHE 116 ILE 243 LEU 379 PRO 380
002 | 016 | 000006.6750 | 00000012798531.0000 | VAL 115 PHE 116 PHE 215 ILE 218 MET 306 THR 314 PRO 374
ALA 375 LEU 379 ALA 484 SER 485 LEU 486 HEM A 600

```
003 | 016 |        000006.6750 | 00000012798531.0000 | VAL   115 PHE   116 PHE   215 ILE   218 MET   306 THR   314 PRO   374
ALA   375 LEU   379 ALA   484 SER   485 LEU   486 HEM A 600
002 | 017 |        000006.6610 | 00000013104554.0000 | LEU   107 PHE   116 LEU   214 PHE   215 ILE   218 ILE   243 ARG   249
MET   306 ILE   309 ALA   310 LEU   379 ALA   484
003 | 017 |        000006.6610 | 00000013104554.0000 | LEU   107 PHE   116 LEU   214 PHE   215 ILE   218 ILE   243 ARG   249
MET   306 ILE   309 ALA   310 LEU   379 ALA   484
004 | 016 |        000006.6540 | 00000013260299.0000 | VAL   115 PHE   116 PHE   215 ILE   218 MET   306 THR   314 PRO   374
ALA   375 LEU   379 ALA   484 SER   485 LEU   486 HEM A 600
002 | 018 |        000006.6450 | 00000013463266.0000 | LYS   173 LYS   212 PHE   215 PHE   216 GLN   480 SER   487 ARG   488
LYS   489 LYS   490 PRO   491 LEU   492
003 | 018 |        000006.6450 | 00000013463266.0000 | LYS   173 LYS   212 PHE   215 PHE   216 GLN   480 SER   487 ARG   488
LYS   489 LYS   490 PRO   491 LEU   492
005 | 018 |        000006.6350 | 00000013692430.0000 | ARG   98 PRO   99 LEU   107 PHE   113 VAL   115 PHE   116 PRO   217
ILE   218 ILE   243 LEU   379 PRO   380
004 | 017 |        000006.6250 | 00000013925494.0000 | LYS   173 LYS   212 PHE   215 PHE   216 GLN   480 THR   483 SER   487
ARG   488 LYS   489 LYS   490 PRO   491 LEU   492
005 | 019 |        000006.6140 | 00000014186450.0000 | THR   101 SER   103 GLY   104 LEU   107 PHE   113 VAL   115 PHE   116
LEU   214 ILE   243 ARG   249 THR   305 MET   306 ILE   309
004 | 018 |        000006.5990 | 00000014550197.0000 | THR   101 SER   103 LEU   107 PHE   116 PRO   217 ILE   218 PRO   223
ILE   242 ILE   243 LEU   379 PRO   380
005 | 020 |        000006.5790 | 00000015049742.0000 | THR   101 SER   103 GLY   104 LEU   107 PHE   113 LEU   214 PRO   217
ILE   218 PRO   223 ILE   243 GLY   246 ARG   249 MET   306 ILE   309
004 | 019 |        000006.5100 | 00000016908564.0000 | LEU   107 PHE   116 PHE   215 ILE   218 THR   305 MET   306 ILE   309
ALA   310 THR   314 LEU   379 ALA   484
002 | 019 |        000006.4890 | 00000017518622.0000 | PHE   116 PHE   215 ILE   218 MET   306 ILE   309 ALA   310 THR   314
ALA   375 LEU   379 ALA   484 HEM A 600
003 | 019 |        000006.4890 | 00000017518622.0000 | PHE   116 PHE   215 ILE   218 MET   306 ILE   309 ALA   310 THR   314
ALA   375 LEU   379 ALA   484 HEM A 600
004 | 020 |        000006.4790 | 00000017816814.0000 | VAL   115 PHE   116 PHE   215 ILE   218 MET   306 ILE   309 ALA   310
GLU   313 THR   314 PRO   374 ALA   375 SER   378 LEU   379 SER   485 HEM A 600
002 | 020 |        000006.4690 | 00000018120080.0000 | PHE   116 PHE   215 ILE   218 THR   305 MET   306 ILE   309 ALA   310
THR   314 PRO   374 ALA   375 LEU   379 ALA   484 SER   485
003 | 020 |        000006.4690 | 00000018120080.0000 | PHE   116 PHE   215 ILE   218 THR   305 MET   306 ILE   309 ALA   310
THR   314 PRO   374 ALA   375 LEU   379 ALA   484 SER   485
```

After clustering the 100 runs, the following 12 distinct complex conformations were found:
[They all differ by at least 5.0 A heavy ligand atom RMSD, and have been saved as YOb files with terminal number "Num"]

```
Num | Rec | Run |Bind.energy[kcal/mol]|Dissoc. constant [pM]| Contacting receptor residues
----+-----+-----+---------------------+---------------------+----------------------------
001 | 001 | 001 |        000008.4390 | 00000000651824.9375 | VAL   115 PHE   116 PHE   215 THR   305 MET   306 ILE   309 ALA
310 THR   314 PRO   374 ALA   375 LEU   379 SER   485 HEM A 600
002 | 001 | 002 |        000008.2390 | 00000000913551.2500 | VAL   115 PHE   116 LEU   214 PHE   215 ILE   218 ARG   249 THR
305 MET   306 ILE   309 ALA   310 LEU   379 HEM A 600
```

```
003 | 001 | 003 |        000007.8600 | 00000001731982.8750 | LEU   107 PHE   113 VAL   115 PHE   116 LEU   214 PHE   215 ILE
218 ILE   243 ARG   249 THR   305 MET   306 ILE   309 ALA   310
004 | 004 | 001 |        000007.8340 | 00000001809680.2500 | ARG   98 PRO   99 LYS   100 THR   101 SER   103 GLY   104 PHE
116 ILE   218 VAL   219 LEU   379 PRO   380
005 | 002 | 001 |        000007.7520 | 00000002078302.0000 | THR   101 SER   103 GLY   104 LEU   107 PHE   113 PHE   116 LEU
214 PRO   217 ILE   218 VAL   219 PRO   223 ILE   243 ARG   249 THR   305 MET   306 ILE   309
006 | 001 | 004 |        000007.7140 | 00000002215965.7500 | VAL   115 PHE   116 PHE   215 MET   306 ALA   310 THR   314 PRO
374 ALA   375 LEU   379 ALA   484 SER   485 HEM A 600
007 | 001 | 005 |        000007.6280 | 00000002562134.2500 | GLY   49 PRO   50 LEU   51 TYR   52 VAL   219 PRO   376 LEU
377 SER   378 LEU   379 PRO   380 LEU   401 ALA   481 GLY   482 THR   483 ALA   484
008 | 005 | 002 |        000007.5590 | 00000002878588.2500 | THR   101 SER   103 GLY   104 LEU   107 PHE   113 VAL   115 PHE
116 LEU   214 PRO   217 ILE   218 PRO   223 ILE   242 ILE   243 MET   306 ILE   309
009 | 004 | 003 |        000007.4920 | 00000003223229.2500 | ARG   98 PRO   99 LYS   100 THR   101 GLY   104 THR   108 PHE
116 ILE   218 VAL   219 PRO   380
010 | 002 | 003 |        000007.4880 | 00000003245063.7500 | LYS   173 LYS   212 PHE   215 PHE   216 VAL   219 GLN   480 GLY
482 THR   483 SER   487
011 | 004 | 005 |        000007.3240 | 00000004279931.5000 | GLY   49 PRO   50 LEU   51 TYR   52 VAL   219 ALA   375 PRO
376 LEU   377 SER   378 LEU   379 ALA   481 GLY   482 THR   483 ALA   484
012 | 005 | 007 |        000007.2040 | 00000005240805.0000 | LYS   173 LYS   212 PHE   215 PHE   216 ILE   218 VAL   219 GLN
480 GLY   482 THR   483 ALA   484 SER   487 PRO   491
```
